# Reported outcomes in studies of intermittent claudication - first step toward a core outcome set: systematic review

**DOI:** 10.1093/bjsopen/zrae126

**Published:** 2024-11-01

**Authors:** Akam Shwan, Segun Lamidi, Calvin Chan, Elizabeth Daniels, Charlie Song-Smith, Lydia Hanna, Viknesh Sounderajah, John S M Houghton, Rob D Sayers

**Affiliations:** Department of Cardiovascular Sciences, University of Leicester, Leicester, UK; Leicester Vascular Institute, University Hospitals of Leicester NHS Trust, Leicester, UK; National Institute for Health Research Leicester Biomedical Research Centre – The Glenfield Hospital, Leicester, UK; Department of Cardiovascular Sciences, University of Leicester, Leicester, UK; Department of Surgery and Cancer, Imperial College London, London, UK; Department of Surgery and Cancer, Imperial College London, London, UK; University College London Medical School, London, UK; Department of Surgery and Cancer, Imperial College London, London, UK; Department of Vascular Surgery, Imperial College Healthcare NHS Trust, London, UK; Department of Surgery and Cancer, Imperial College London, London, UK; Department of Cardiovascular Sciences, University of Leicester, Leicester, UK; Leicester Vascular Institute, University Hospitals of Leicester NHS Trust, Leicester, UK; National Institute for Health Research Leicester Biomedical Research Centre – The Glenfield Hospital, Leicester, UK; Department of Cardiovascular Sciences, University of Leicester, Leicester, UK; Leicester Vascular Institute, University Hospitals of Leicester NHS Trust, Leicester, UK; National Institute for Health Research Leicester Biomedical Research Centre – The Glenfield Hospital, Leicester, UK

## Abstract

**Introduction:**

This review aimed to compile an exhaustive list of all outcome measures and identify different characteristics of the outcomes reported in studies of intermittent claudication as the first step in developing a core outcome set for intermittent claudication.

**Method:**

Medline and Embase were searched for all studies including individuals with intermittent claudication and reporting ≥1 outcome from January 2015 to August 2024. Abstract, full text screening and data extraction were performed by two investigators independently. All reported outcome measures were extracted verbatim and categorized by Dodd’s domains (Core Outcome Measures in Effectiveness Trials registration: COMIC Study, 1590; https://www.comet-initiative.org/Studies/Details/1590).

**Results:**

4985 studies were screened and 408 were included. A total of 541 unique outcomes across 25 Dodd’s domains were identified. Ankle–brachial pressure index was the most frequently reported outcome. Among the 541 unique outcomes, 386 outcomes were only reported once. Only 38.9% of the studies exclusively included patients with intermittent claudication. Patient-reported outcomes were reported in 36.2% of studies. There were wide variations in the definition of commonly used outcome measures (for example, major adverse limb event and primary patency) across different studies.

**Conclusion:**

There is substantial heterogeneity in reported outcomes in studies of intermittent claudication. Most reported outcomes are clinical/physiology oriented rather than patient centred. Development of a core outcome set for intermittent claudication is vital to improve and standardize reporting in future research.

## Teaser

This review shows the substantial heterogeneity, lack of use of PROMs, and wide variations of reported outcomes in research of people with intermittent claudication and demonstrates the need for a core outcome set for research in intermittent claudication.

## Introduction

Peripheral artery disease (PAD) is a common atherosclerotic condition affecting the arteries supplying the lower limbs. PAD covers a spectrum of presentations ranging from asymptomatic disease through to intermittent claudication, and, terminally, chronic limb-threatening ischaemia (CLTI)^1–3^. PAD is associated with a significantly increased risk of overall cardiovascular morbidity and mortality, and the annual incidence of PAD continues to rise globally^4,5^. As of the early 2010s, PAD is firmly established as the third leading cause of atherosclerotic cardiovascular morbidity, following coronary artery disease and stroke^6–8^.

There has been a sharp increase in the number of clinical trials focused upon improving the outcomes of intermittent claudication, the most prevalent subgroup of symptomatic PAD^3,9^. However, despite this, there remains no consensus on which outcomes should be measured within research studies of this group of patients^10^. This lack of standardization limits both the objective comparison between studies and the generation of pooled effect estimates of intervention efficacy in meta-analysis^10,11^. There have been various attempts to organize and group reported outcomes in PAD; for example, the International Consortium of Vascular Registries Consensus Recommendations for Peripheral Revascularisation Registry Data Collection^12^. However, these recommendations are still huge in number with wide variations in the suggested outcomes, and their use is limited to specific scenarios.

Historically, choice of outcome measures in clinical trials has varied widely and led to significant research waste^13^. Outcomes reported in trials are not always those that patients regard as most important or relevant^14^. Inconsistencies and wide variations in outcomes have caused major issues in utilization of healthcare research^13^. These issues can be tackled and reduced with development of a standardized, agreed, minimum set of reported outcome measures in trials of a specific condition, which is known as a core outcome set (COS). The aim of a COS is to have a standard of measures to be reported in every clinical trial and study pertaining to a particular condition, treatment or intervention^15^. These outcomes are regarded as the minimum that should be reported by relevant trials. There has been an increase in developing COS in various medical fields, but to date, the only COS involving patients with PAD was developed by Ambler *et al.* for patients undergoing major lower limb amputation for complications of PAD^15,16^.

This systematic review aimed to generate a list of all the outcomes that have been reported in studies involving patients with intermittent claudication as a first step toward development of a COS for research involving patients with intermittent claudication.

## Methods

This systematic review was conducted according to the PRISMA Statement and Core Outcome Measures in Effectiveness Trials (COMET) Handbook^15,17^ (COMET Registration: COMIC Study, 1590; https://www.comet-initiative.org/Studies/Details/1590).

### Search strategy

The initial database search of both MEDLINE and EMBASE was conducted through Ovid from January 2015 to September 2020. The search was rerun in February 2023 and again in August 2024 to include as many studies as possible. Medical Subject Headings (MeSH) or EMBASE Subject Headings (Emtree) as well as keywords with proximity and wild-card operators for the two concepts of the review—the disease (for example, Intermittent Claudication/, peripheral arterial disease, leg adj3 occlus*) and the intervention (for example, exercise therapy/, walking)—were used (*Table S1*, *S2*).

### Study selection

Titles and abstracts were screened by two reviewers independently (AS plus ED, SL or CC). Full texts were then screened by two independent reviewers (AS plus ED, SL, CC or CS). Disagreements were resolved by discussion. All clinical studies reporting at least one outcome involving patients with symptomatic intermittent claudication were included. Studies including a mix of cohorts of patients with intermittent claudication with asymptomatic and/or patients with CLTI were included to ensure the breadth of the review and minimize the risk of missing important outcome measures. As the primary objective of this review was to compile an exhaustive list of study outcomes, not to assess intervention effectiveness, all study types were included consisting of both observational studies and randomized clinical trials (RCTs). Grey literature and non-English language studies without an available translation were not included. Reference lists of included studies and relevant systematic reviews identified in the search strategy were hand searched for additional records.

### Data collection and extraction

All data were extracted by two independent authors (AS plus ED, SL, CC or CS). Disagreements were resolved through discussion. Data extraction was performed onto a standardized proforma. Outcomes and their definitions were extracted verbatim from the source manuscripts, as recommended in the COMET Handbook^15^. If individual components of a particular quality-of-life (QoL) tool were used as an independent outcome (for example, the ambulatory ability component of the 36-Item Short Form Survey (SF-36)), they were extracted as separate outcome measures. Outcomes that measured the same clinical, physiological or QoL parameter but with different descriptions were grouped together (for example, for ankle–brachial pressure index (ABPI) ‘Change in ABPI’ and ‘Improvement in ABPI’ were grouped together with ‘ABPI’). All the extracted outcomes were categorized as per the taxonomy for outcomes in medical research developed by Dodd *et al*.^18^. The reported outcomes were grouped per Dodd’s taxonomy based on their uniqueness; for example, all-cause mortality at 1 year or 2 years would still capture all-cause mortality, whereas all-cause mortality, cardiovascular death and non-cardiovascular death each are unique and capture different outcomes. Assessment of study quality or risk of bias was not necessary as this review considered only the outcomes themselves rather than the effect size.

### Analysis of results

Results were tabulated and a narrative synthesis of results was undertaken for all studies and outcome measures. Sensitivity analysis was performed including only studies that exclusively recruited patients with intermittent claudication.

## Results

### Study selection and characteristics

**Fig. 1:**
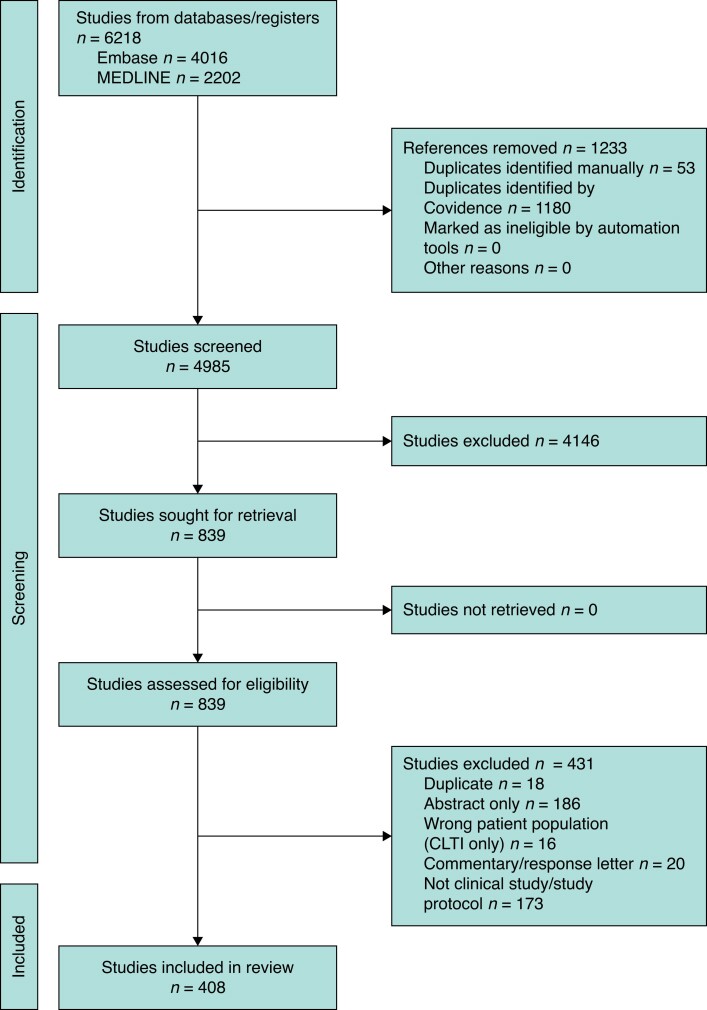
Study selection flow diagram CLTI, chronic limb-threatening ischaemia.

**Fig. 2:**
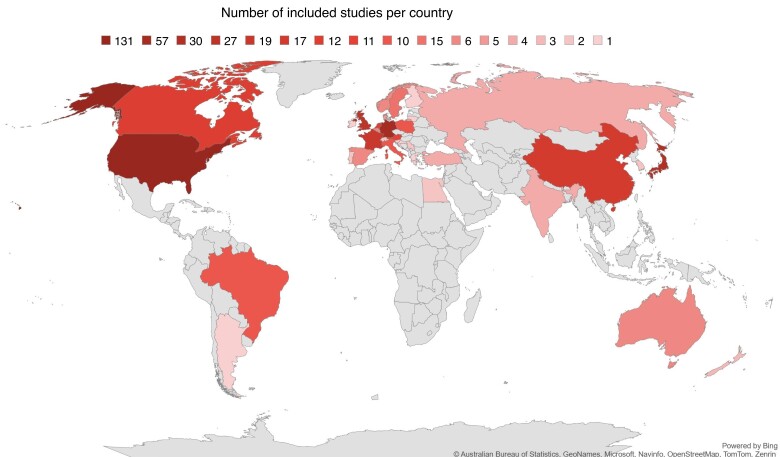
Number of included studies per country

Of the total of 4 985 titles and abstracts screened, 839 full-text articles were assessed and 408 studies were included (*Fig. 1*). These studies included a total of 386 777 patients (range 5–50 168) across 35 countries (*Fig. 2*).

In total, 159 studies (38.9%) included only individuals with intermittent claudication and 227 studies (55.6%) included participants with both claudication and CLTI. A further 12 studies (2.9%) included both people with intermittent claudication and asymptomatic PAD, and ten studies (2.4%) included a combination of participants with asymptomatic PAD, claudication and CLTI.

More than half of the studies (*n* = 243, 59.5%) were RCTs, followed by 59 (14.4%) prospective cohort studies, 55 (13.4%) retrospective cohort studies and 51 (12.5%) single-arm trials.

### Reported outcome measures

A total of 2 386 outcomes were reported across the included studies, with a median of six separate outcome measures (range 1–16) reported by each study.

There were 541 unique outcome measures reported in the included studies (*Table S3*). Of these, 386 (71.3%) were reported only once across all the included studies, 56 (10.3%) twice and 21 (3.8%) three times only. Outcome measures that were reported fewer than ten times across all included studies comprised 92.2% (*n* = 499) of the total outcome measures.

ABPI was the most commonly reported outcome measure, being reported in 146 studies, followed by primary patency (103 studies), and 6-min walk test (88 studies). There were 55 patient-reported outcome measures (PROMs; 10.1% of all outcome measures) reported across the included studies, with almost two-thirds of included studies (63.8%) not reporting any PROMs (*Table 1*). The most commonly reported PROM was the walking impairment questionnaire (WIQ; 81 studies). This was the only PROM in the top 10 most frequently reported outcome measures (*Table 2*).

**Table 1:** Patient-reported outcome measures reported throughout the studies

Outcome measure	Frequency	Dodd’s domain
Walking impairment questionnaire	81	25. Physical functioning
SF-36	52	30. Global quality of life
EQ-5D-5L	35	30. Global quality of life
EQ-5D-3L	27	30. Global quality of life
VascuQoL-6	20	25. Physical functioning
PADQOL	6	30. Global quality of life
Peripheral artery questionnaire	6	30. Global quality of life
SF-12	6	30. Global quality of life
VascuQol-25	6	30. Global quality of life
Hospital anxiety and depression scale	5	28. Emotional functioning
Borg’s rating of perceived exertion scale	3	31. Perceived health status
Pain rating score	2	9. General outcome
Claudication scale (CLAU-S)	2	24. Vascular outcomes
Intermittent claudication questionnaire	2	25. Physical functioning
San Diego claudication questionnaire	2	25. Physical functioning
SARC-F	2	25. Physical functioning
Walking estimated-limitation calculated by history	2	25. Physical functioning
HLS-EU-Q16	2	27. Role functioning
Theory planned behaviour questionnaire	2	27. Role functioning
SF-12 V2	2	30. Global quality of life
WHOQOL-Bref	2	30. Global quality of life
10 cm visual analogue scale of pain	1	9. General outcome
Brief pain inventory score	1	9. General outcome
Participant’s opinions of shoes	1	9. General outcome
Wong–Baker faces pain scale	1	9. General outcome
Claudication pain on visual analogue scale	1	24. Vascular outcomes
Claudication symptom rating scale	1	24. Vascular outcomes
Barthel questionnaire	1	25. Physical functioning
Brief international physical activity questionnaire	1	25. Physical functioning
Chalder fatigue scale	1	25. Physical functioning
Edinburgh claudication questionnaire	1	25. Physical functioning
International physical activity questionnaire	1	25. Physical functioning
McGill pain questionnaire	1	25. Physical functioning
Modified walking impairment questionnaire	1	25. Physical functioning
Nottingham extended activities of daily living	1	25. Physical functioning
Patient-specific functional scale	1	25. Physical functioning
PROMIS physical function	1	25. Physical functioning
WHO/Rose questionnaire	1	25. Physical functioning
AUDIT-C questionnaire	1	26. Social functioning
Fagerström test for nicotine dependence	1	26. Social functioning
Barriers specific questionnaire	1	27. Role functioning
General self-efficacy scale	1	27. Role functioning
PAM-13 questionnaire	1	27. Role functioning
Feeling scale (Analog)	1	28. Emotional functioning
GAD-7 questionnaire	1	28. Emotional functioning
PHQ-9 questionnaire	1	28. Emotional functioning
5 point patient safety questionnaire	1	30. Global quality of life
AUSVIQUOL	1	30. Global quality of life
Treatment self-regulation questionnaire	1	30. Global quality of life
Validated 10-item questionnaire	1	30. Global quality of life
Brief illness perception questionnaire	1	31. Perceived health status
Disease-specific self-rated health and symptoms	1	31. Perceived health status
Revised illness perception questionnaire	1	31. Perceived health status
Subjective pain sensation score	1	31. Perceived health status
Satisfaction (using 0–100 analogue scale)	1	32. Delivery of care

AUDIT-C, Alcohol Use Disorders Identification Test Consumption; AUSVIQUOL, Australian Vascular Quality of Life Index; EQ-5D-3L, EuroQoL Three Options, Five Dimensions Quality of Life Questionnaire with Visual Analogue Scale; EQ-5D-5L, EuroQoL Five Options Five Dimensions Quality of Life Questionnaire with Visual Analogue Scale; GAD-7, 7-Question General Anxiety Disorder Questionnaire; HLS-EU-Q16, European Health Literacy Questionnaire; PADQOL, Peripheral Arterial Disease Quality of Life questionnaire; PAM-13, 13-Question Patient Activation Measure; PHQ-9, 9-Question Patient Health Questionnaire; PROMIS Physical Function, Physical Function component of Patient Reported Outcomes Measurement Information System; SARC-F, Sarcopenia screening questionnaire; SF-12, 12-item Short Form survey; SF-36, 36-item Short Form survey; VascQoL-6, 6-Question Vascular Quality of Life Questionnaire; VascQol-25, 25-Question Vascular Quality of Life Questionnaire.

**Table 2:** Top 10 most frequently reported outcomes across all the included studies

Reported outcome	Frequency (*n*)
Ankle–brachial pressure index	146
Primary patency	103
6-minute walk test	88
Rutherford–Becker classification	86
Walking impairment questionnaire	81
Target lesion revascularization	79
Intermittent claudication distance	73
Maximal walking distance	71
Clinically driven target lesion revascularization	62
Major adverse events	58

Classifying the unique outcomes per Dodd’s taxonomy, 63.9% (*n* = 318) of the outcomes were clinical/physiological compared to 22.9% (*n* = 124) Life Impact outcomes. Death, resource use and adverse events/effects comprised 5.7% (*n* = 31), 3.8% (*n* = 21) and 3.5% (*n* = 19) respectively (*Table 3*).

**Table 3:** Frequency of reported outcomes per studies classified per Dodd’s taxonomy

Dodd’s core area	Dodd’s domain*		Number of unique outcomes per domain	Number of studies reporting each domain
Death	1.	Mortality/survival	31	117
Physiological or clinical	2.	Blood and lymphatic system	1	1
	3.	Cardiac	21	36
	9.	General outcomes	41	40
	10.	Hepatobiliary	1	1
	11.	Immune system	5	7
	12.	Infection and infestation	6	9
	14.	Metabolism and nutrition	7	9
	15.	Musculoskeletal and soft tissue	13	7
	17.	Nervous system	8	20
	19.	Renal and urinary	5	4
	22.	Respiratory, thoracic and mediastinal	4	3
	23.	Skin and subcutaneous tissue	7	6
	24.	Vascular	227	297
Life impact	25.	Physical functioning	66	197
	26.	Social functioning	2	1
	27.	Role functioning	2	4
	28.	Emotional functioning/well-being	3	5
	30.	Global quality of life	18	109
	31.	Perceived health status	8	12
	32.	Delivery of care	25	60
Resource use	34.	Economic	9	10
	35.	Hospital	5	8
	36.	Need for further intervention	7	7
Adverse events	38.	Adverse events/effects	19	74

^*^Dodd’s taxonomy classifies all measurable outcomes into 38 different domains across five distinct core areas (the numbers preceding each domain correspond to the original numbering in Dodd’s taxonomy and hence are not in perfect order). All reported outcomes in this study fall into 25 of the total 38 domains^18^.

Wide variations in definitions of commonly used composite outcomes were noted across the studies. Major adverse limb events (MALEs) had 14 different definitions across the 46 studies that reported it as an outcome (*Table 4*); similarly for major adverse events with 28 (*Table S4*) and primary patency with 18 distinct definitions (*Table S5*).

**Table 4:** Variations in definition of MALE

Definition of MALE	Number of studies
Composite of acute limb ischaemia and amputation	12
Freedom from target limb major amputation and clinically driven target vessel revascularization	11
Freedom from target limb major amputation and clinically driven target lesion revascularization	5
MALE including ALI, major amputation of a vascular aetiology, and unplanned limb revascularization	3
Composite end point (MALE) of acute limb ischaemia, urgent peripheral revascularization, and major vascular amputation	2
MALE surgical bypass of the target limb, target limb amputation above the ankle, and/or embolization or vessel thrombosis requiring thrombolysis in the target limb	1
Time to the onset and incidence of peripheral vascular events (incidence of amputation, revascularization, or bypass surgery)	1
30-day MALE (defined as major amputation and/or major reintervention requiring surgical bypass of the index limb or thrombolysis/thrombectomy)	1
Limb ischaemic adverse events	1
MALE, defined as a major amputation or any reintervention, including surgical or endovascular reintervention	1
MALE (composite of limb-related death, target lesion revascularization, major amputation, major bleeding, and definite or probable stent thrombosis)	1
MALE (above-ankle amputation of the index limb, major graft reintervention with placement of a new graft or an interposition graft, open or percutaneous graft thrombectomy, pharmacologic thrombolysis, or graft infection)	1
MALE (composite of acute limb ischaemia or major amputation of vascular aetiology)	1
MALE (major amputation or any intervention/revascularization)	1
No given definition in the study (protocol of the study not published/not available)	1

MALE, major adverse limb event.

### Sensitivity analysis

Only 38.9% (*n* = 159) of the studies included patients exclusively with intermittent claudication. Intermittent Claudication Distance was the most reported outcome in this subgroup of studies, being reported in 64 of the total 159 studies of patients with intermittent claudication. The next most frequently reported outcomes were maximal walking distance (*n* = 62) and ABPI (*n* = 56). Four PROMs (SF-36, WIQ, VascQol-6 and EQ-5D-5L) were in the top 10 most frequently reported outcome measures (*Table 5*).

**Table 5:** Top 10 most frequently reported outcomes across studies enrolling intermittent claudication patients only

Reported outcome	Frequency (*n*)
Intermittent claudication distance	64
Maximal walking distance	62
Ankle–brachial pressure index	56
6-minute walk test	52
SF-36	38
Walking impairment questionnaire	30
Claudication onset time	24
VascuQoL-6	18
Maximal walking time	17
EQ-5D-5L	11

EQ-5D-5L, EuroQoL Group Five Dimensional 5-Option Quality of Life Questionnaire; SF-36, 36-item Short Form survey; VascQoL-6, King’s College Hospital’s 6-Question Vascular Quality of Life Questionnaire.

Similar variations in reported outcomes were observed when comparing RCTs to observational studies, with ABPI, primary patency and Rutherford Classification being the top three most frequently reported outcomes in descending order. The use of PROMs was slightly more frequent in RCTs (11.9%) compared to observational studies (9.4%).

## Discussion

This systematic review identified 541 unique outcomes reported across 408 studies enrolling patients with intermittent claudication. Most of the outcomes have been reported only once or twice, limiting comparison of outcomes between studies and interventions.

The vast number and heterogeneity of outcomes among the studies included in this review is striking and limits the generalizability of individual study findings. Further methodological problems identified in this review include absence of separate reporting of outcomes of participants with intermittent claudication, asymptomatic PAD and CLTI in studies including a mixed cohort of patients. Additionally, this review has identified a lack of PROMs usage in the majority of included studies as well as wide variations in definitions of the commonly used composite outcome measures (for example, MALE). In combination, these issues in outcome choice, definition and reporting limit the comparability of studies and interventions, and in many cases mean pooling of results in meta-analysis is not possible.

Enrolling patients with intermittent claudication and CLTI together can be helpful in increasing the sample size and therefore the power of a study, and over half of the studies included in this review recruited a mixed cohort of patients with claudication and CLTI. However, both presentation and prognosis of these two groups of patients are very different^9^. Intervention for claudication is primarily performed to improve quality of life and walking distance, whereas intervention for CLTI is usually for limb salvage^9,19^. As such, the two groups are likely to have very different prioritization of outcomes. At the very least, studies including patients with both intermittent claudication and CLTI should report outcomes separately for each subgroup as well as aggregate results for the whole sample. Again, lack of separate reporting of studies with mixed cohorts of patients is a barrier to comparison of studies and interventions and pooling in meta-analysis. It also represents inappropriateness of the research as the outcomes reported are unlikely to have equal relevance to both patient groups.

Despite the increasing acknowledgement of the importance and call for greater utilization of PROMs in research, only a third of the studies included in this review reported one or more PROM^14^. The majority of the reported outcomes were at limb-level (for example, ABPI) or are oriented toward the technical success of surgical or endovascular interventions (for example, primary patency). For results of research to be meaningful to patients and to aid shared decision-making, PROMs are essential to demonstrate efficacy of interventions^20^. An improvement in ABPI does not necessarily translate to improvement in symptoms reported by patients, hence using a PROM can be more insightful than using a physiological measure alone^21,22^. On top of the lack of use of PROMs, despite the fact that most commonly used questionnaires across the studies are validated (*Table 1*), to our knowledge there are no available data directly comparing their reliability and validity and no recommendation on adopting a specific questionnaire over another has been made.

Variations in definitions of commonly used composite outcomes is yet another source of heterogeneity identified in this review. There were 14 different definitions of MALE (*Table 4*), 28 distinct definitions of major adverse events and 18 definitions of primary patency (*Table S4*, *S5*), each with a set of specific outcomes that are quite different to each other, or in some instances no definition was given at all. Adherence to available reporting standards may be a cause for these variations. Zywicka *et al.* reported extremely poor adherence to reporting standards in RCTs investigating endovascular interventions^23^. This could be inherently true in non-RCT studies too, but lack of consensus on definitions of commonly used outcomes is a major issue plaguing current literature, again leading to inability to accurately compare the efficacy of the reported interventions. Agreed definitions of the commonly used outcomes following a development of COS in studies of PAD is also crucial and would further improve outcome reporting and comparability of results.

Research waste is a well-recognized problem in health research. It is defined as avoidable inappropriate conduct and dissemination of research^24^. It is estimated that research waste cost the USA US$85 billion in 2009 alone^13,25^. The numerous issues in research in patients with intermittent claudication highlighted in this review can directly and indirectly contribute to all four areas leading to research waste, highlighted by Chalmers and Glasziou in the form of ‘the choice of research questions; the quality of research design and methods; the adequacy of publication practices; and the quality of reports of research’^26^. The numerous issues with the reported outcomes highlight the necessity of a COS for research in patients with intermittent claudication. The process for development of a COS is well defined and seeks to involve all key stakeholders: patients (and carers), healthcare professionals, researchers, policy-makers and industry representatives. Development and adoption of a COS for intermittent claudication would significantly improve the quality, relevance and comparability of studies and significantly reduce research waste. The long list of all the reported outcomes identified in this review will be further supplemented with interviews with patients and carers as well as focus groups with healthcare professionals to identify any potential outcome measures not identified in this review. The final list will then be narrowed down in a three-phase Delphi Consensus and a final stakeholder meeting to agree on the COS for intermittent claudication (COMET registration: COMIC Study, 1590).

The main strength of this systematic review is its breadth, including 408 studies enrolling >300 000 participants and identifying 541 unique outcome measures. There are, however, several limitations. Inclusion of studies enrolling mixed cohorts of participants with PAD may have skewed outcome measure priority given the varying relevance of different outcome measures among individuals with different stages of PAD. Therefore, subgroup analysis of studies exclusively including participants with intermittent claudication was also performed, which had comparable results. Restricting the search strategy to studies published from 2015 onwards may have led to a small number of outcome measures not being identified that are reported in the studies prior to the included study timeline. However, re-running the searches twice in February 2023 and August 2024 for studies published since the original search was performed in September 2020 identified only 52 additional outcomes measures, with 37 of the 52 outcomes being reported once and another 9 only twice. It is therefore unlikely that extending the search prior to 2015 would have identified a meaningful number of relevant new outcome measures. No assessment of study quality was required given the nature of the review, however the number (over 500) and breadth of outcome measures (25 Dodd’s domains) identified meant assessment of quality of individual outcome measures could not be performed and was outside the scope of this review. Therefore, no recommendations can be made on which outcome measures should be used in studies of patients with intermittent claudication at this stage. Future research assessing quality of individual outcomes measures (for example, reliability, validity) of varying types of outcome measures (for example, PROMs) is required as part of implementation of a COS, once developed. Also, consensus and standardization of specific outcome measures (for example, MALE, primary patency) are needed to further improve research and ultimately patient care among individuals with PAD.

Although there is a substantial amount of high-quality research evaluating the efficacy of management of symptomatic intermittent claudication in patients with PAD, current work is limited by the quantity and heterogeneity of reported outcome measures, which limits comparison and pooling of available data. Development of COS for intermittent claudication research is essential for capturing the most important issues facing patients, carers, healthcare professionals as well as policy-makers in future research in patients with intermittent claudication.

## Supplementary Material

zrae126_Supplementary_Data

## Data Availability

All the study data can be made available on request. Please contact the corresponding author.

## References

[1] Aday AW, Matsushita K. Epidemiology of peripheral artery disease and polyvascular disease. Circ Res 2021;128:1818–1832 34110907 10.1161/CIRCRESAHA.121.318535 PMC8202714

[2] Criqui MH, Matsushita K, Aboyans V, Hess CN, Hicks CW, Kwan TW et al Lower extremity peripheral artery disease: contemporary epidemiology, management gaps, and future directions: a scientific statement from the American Heart Association. Circulation 2021;144:e171–e191 34315230 10.1161/CIR.0000000000001005 PMC9847212

[3] Nordanstig J, Behrendt CA, Baumgartner I, Belch J, Back M, Fitridge R et al Editor's choice—European Society for Vascular Surgery (ESVS) 2024 clinical practice guidelines on the management of asymptomatic lower limb peripheral arterial disease and intermittent claudication. Eur J Vasc Endovasc Surg 2024;67:9–96 37949800 10.1016/j.ejvs.2023.08.067

[4] Song P, Rudan D, Zhu Y, Fowkes FJI, Rahimi K, Fowkes FGR et al Global, regional, and national prevalence and risk factors for peripheral artery disease in 2015: an updated systematic review and analysis. Lancet Glob Health 2019;7:e1020–e1030 31303293 10.1016/S2214-109X(19)30255-4

[5] National Institute for Health and Care Excellence . Peripheral arterial disease: diagnosis and management (CG147). London 2012 (updated 2020) 32073808

[6] Fowkes FG, Rudan D, Rudan I, Aboyans V, Denenberg JO, McDermott MM et al Comparison of global estimates of prevalence and risk factors for peripheral artery disease in 2000 and 2010: a systematic review and analysis. Lancet 2013;382:1329–1340 23915883 10.1016/S0140-6736(13)61249-0

[7] Nehler MR, Duval S, Diao L, Annex BH, Hiatt WR, Rogers K et al Epidemiology of peripheral arterial disease and critical limb ischemia in an insured national population. J Vasc Surg 2014;60:686–95.e2 24820900 10.1016/j.jvs.2014.03.290

[8] Vascular Services Quality Improvement Programme. 2022 Annual Report. 2022. https://www.vsqip.org.uk/reports-publications/2022-annual-report/ (accessed 20 March 2023)

[9] Kengne AP, Echouffo-Tcheugui JB. Differential burden of peripheral artery disease. Lancet Glob Health 2019;7:e980–e981 31303302 10.1016/S2214-109X(19)30293-1

[10] Lane R, Harwood A, Watson L, Leng GC. Exercise for intermittent claudication. Cochrane Database Syst Rev 2017;12:CD000990 29278423 10.1002/14651858.CD000990.pub4 PMC6486315

[11] Treat-Jacobson D, McDermott MM, Bronas UG, Campia U, Collins TC, Criqui MH et al Optimal exercise programs for patients with peripheral artery disease: a scientific statement from the American Heart Association. Circulation 2019;139:e10–e33 30586765 10.1161/CIR.0000000000000623

[12] Behrendt CA, Bertges D, Eldrup N, Beck AW, Mani K, Venermo M et al International Consortium of Vascular Registries consensus recommendations for peripheral revascularisation registry data collection. Eur J Vasc Endovasc Surg 2018;56:217–237 29776646 10.1016/j.ejvs.2018.04.006

[13] Grainger MJ, Bolam FC, Stewart GB, Nilsen EB. Evidence synthesis for tackling research waste. Nat Ecol Evol 2020;4:495–497 32203478 10.1038/s41559-020-1141-6

[14] Mercieca-Bebber R, King MT, Calvert MJ, Stockler MR, Friedlander M. The importance of patient-reported outcomes in clinical trials and strategies for future optimization. Patient Relat Outcome Meas 2018;9:353–367 30464666 10.2147/PROM.S156279 PMC6219423

[15] Williamson PR, Altman DG, Bagley H, Barnes KL, Blazeby JM, Brookes ST et al The COMET handbook: version 1.0. Trials 2017;18:280 28681707 10.1186/s13063-017-1978-4 PMC5499094

[16] Ambler GK, Brookes-Howell L, Jones JAR, Verma N, Bosanquet DC, Thomas-Jones E et al Development of core outcome sets for people undergoing major lower limb amputation for complications of peripheral vascular disease. Eur J Vasc Endovasc Surg 2020;60:730–738 32798206 10.1016/j.ejvs.2020.06.021

[17] Moher D, Liberati A, Tetzlaff J, Altman DG, Group P. Preferred reporting items for systematic reviews and meta-analyses: the PRISMA statement. PLoS Med 2009;6:e1000097 19621072 10.1371/journal.pmed.1000097 PMC2707599

[18] Dodd S, Clarke M, Becker L, Mavergames C, Fish R, Williamson PR. A taxonomy has been developed for outcomes in medical research to help improve knowledge discovery. J Clin Epidemiol 2018;96:84–92 29288712 10.1016/j.jclinepi.2017.12.020 PMC5854263

[19] Roijers JP, van den Houten MM, Hopmans NJ, Vriens P, Willigendael EM, Lodder P et al A comparison of quality of life in elderly patients with intermittent claudication and chronic limb-threatening ischemia. Ann Vasc Surg 2020;69:285–291 32502674 10.1016/j.avsg.2020.05.048

[20] Rymer JA, Narcisse D, Cosiano M, Tanaka J, McDermott MM, Treat-Jacobson DJ et al Patient-reported outcome measures in symptomatic, non-limb-threatening peripheral artery disease: a state-of-the-art review. Circ Cardiovasc Interv 2022;15:e011320 34937395 10.1161/CIRCINTERVENTIONS.121.011320

[21] Hammad TA, Smolderen KG, Spertus JA, Jones PG, Shishehbor MH. Associations of exercise ankle–brachial index, pain-free walking distance and maximum walking distance with the peripheral artery questionnaire: finding from the PORTRAIT PAD registry. Vasc Med 2019;24:32–40 29992849 10.1177/1358863X18785026

[22] Long J, Modrall JG, Parker BJ, Swann A, Welborn MB 3rd, Anthony T. Correlation between ankle–brachial index, symptoms, and health-related quality of life in patients with peripheral vascular disease. J Vasc Surg 2004;39:723–727 15071432 10.1016/j.jvs.2003.12.006

[23] Zywicka EM, McNally E, Elliott L, Twine CP, Mouton R, Hinchliffe RJ. Exploring the reporting standards of randomised controlled trials involving endovascular interventions for peripheral arterial disease: a systematic review. Eur J Vasc Endovasc Surg 2024;67:155–164 37678660 10.1016/j.ejvs.2023.08.066

[24] Rutherford C, Boehnke JR. Introduction to the special section “Reducing research waste in (health-related) quality of life research”. Qual Life Res 2022;31:2881–2887 35907111 10.1007/s11136-022-03194-z

[25] Chalmers I, Bracken MB, Djulbegovic B, Garattini S, Grant J, Gulmezoglu AM et al How to increase value and reduce waste when research priorities are set. Lancet 2014;383:156–165 24411644 10.1016/S0140-6736(13)62229-1

[26] Chalmers I, Glasziou P. Avoidable waste in the production and reporting of research evidence. Lancet 2009;374:86–89 19525005 10.1016/S0140-6736(09)60329-9

