# Supplementary material for: Reported outcomes in studies of intermittent claudication - first step toward a core outcome set: systematic review
Source: BJS Open. 2024 Nov 1;8(6):zrae126. doi: 10.1093/bjsopen/zrae126 (PMC11529788; doi:10.1093/bjsopen/zrae126)
Supplement: zrae126_Supplementary_Data [file zrae126_supplementary_data.zip › Supplementary_material.docx]

**Title:** Comprehensive Systematic Review of Reported Outcomes in Studies of Intermittent Claudication: First Step Toward a Core Outcome Set

**Authors:** Akam Shwan^1,4,6 (corresponding author)^, Segun Lamidi^1^, Calvin Chan^2^, Elizabeth Daniels^2^, Charlie Song-Smith^3^, Lydia Hanna^2,5^, Viknesh Sounderajah^2^, John SM Houghton^1,4,6^, Rob D Sayers^1,4,6^.

**Author affiliations:** ^1^Department of Cardiovascular Sciences, University of Leicester, ^2^Department of Surgery and Cancer, Imperial College London, ^3^University College London Medical School, ^4^Leicester Vascular Institute, University Hospitals of Leicester NHS Trust, ^5^Department of Vascular Surgery, Imperial College Healthcare NHS Trust, ^6^National Institute for Health Research Leicester Biomedical Research Centre – The Glenfield Hospital, Leicester.

**Correspondence to:**

Akam Shwan

Department of Cardiovascular Sciences

College of Life Sciences, University of Leicester

OC6, On-Call Suite, Glenfield Hospital, Groby Road

Leicester, UK

LE3 9QP

t: +44(0)1162523867

e: [akam.shwan@nhs.net](mailto:jsmh2@leicester.ac.uk), as1610@le.ac.uk
x: @aoashwan

**Supplementary Materials - Index**

**Supplementary Methods**

Table 1. [Search strategy conducted in Medline via Ovid 2](#_Toc149120965)

Table 2. [Search strategy conducted in Embase via Ovid 3](#_Toc149120966)

**Supplementary Tables**

Table 3. [List of unique outcomes reported throughout the studies 4](#_Toc149120967)

Table 4. [Variations in definitions of Major Adverse Events 16](#_Toc149120968)

Table 5. [Variations in definitions of Primary Patency 17](#_Toc149120969)

Table 6. [Complete list of included studies 18](#_Toc149120970)

[**References** 36](#_Toc149120971)

# **Table 1. Search strategy conducted in Medline via Ovid**

| **Item number** | **Search Item** |
| --- | --- |
| 1 | Intermittent Claudication/ |
| 2 | claudic*.mp. |
| 3 | exp Peripheral Vascular Diseases/ |
| 4 | Arterial Occlusive Diseases/ |
| 5 | Peripheral Arterial Disease/ |
| 6 | 1 or 2 or 3 or 4 or 5 |
| 7 | randomized controlled trial.pt. |
| 8 | controlled clinical trial.pt. |
| 9 | clinical trial.pt. |
| 10 | comparative study.pt. |
| 11 | randomized.ab. |
| 12 | placebo.ab. |
| 13 | clinical trials as topic.sh. |
| 14 | randomly.ab. |
| 15 | trial.ti. |
| 16 | 7 or 8 or 9 or 11 or 12 or 13 or 14 or 15 |
| 17 | exp animals/ not humans.sh. |
| 18 | 16 not 17 |
| 19 | 6 and 18 |
| 20 | limit 19 to english language |

# **Table 2. Search strategy conducted in Embase via Ovid**

| **Item number** | **Search Item** |
| --- | --- |
| 1 | intermittent claudication/ |
| 2 | "claudic*".af. |
| 3 | peripheral vascular disease/ |
| 4 | peripheral occlusive artery disease/ |
| 5 | 1 or 2 or 3 or 4 |
| 6 | Randomized controlled trial/ |
| 7 | Controlled clinical study/ |
| 8 | random$.ti,ab. |
| 9 | randomization/ |
| 10 | placebo.ti,ab. |
| 11 | trial.ti. |
| 12 | or/6-11 |
| 13 | (random$ adj sampl$ adj7 ("cross section$" or questionnaire$1 or survey$ or database$1)).ti,ab. not (comparative study/ or controlled study/ or randomi?ed controlled.ti,ab. or randomly assigned.ti,ab.) |
| 14 | Cross-sectional study/ not (randomized controlled trial/ or controlled clinical study/ or controlled study/ or randomi?ed controlled.ti,ab. or control group$1.ti,ab.) |
| 15 | (((case adj control$) and random$) not randomi?ed controlled).ti,ab. |
| 16 | (Systematic review not (trial or study)).ti. |
| 17 | (nonrandom$ not random$).ti,ab. |
| 18 | "Random field$".ti,ab. |
| 19 | (review.ab. and review.pt.) not trial.ti. |
| 20 | "we searched".ab. and (review.ti. or review.pt.) |
| 21 | "update review".ab. |
| 22 | (databases adj4 searched).ab. |
| 23 | (rat or rats or mouse or mice or swine or porcine or murine or sheep or lambs or pigs or piglets or rabbit or rabbits or cat or cats or dog or dogs or cattle or bovine or monkey or monkeys or trout or marmoset$1).ti. and animal experiment/ |
| 24 | Animal experiment/ not (human experiment/ or human/) |
| 25 | or/13-24 |
| 26 | 12 not 25 |
| 27 | 5 and 26 |
| 28 | limit 27 to (english language and yr="2015 -Current") |

# **Table 3. List of all unique outcomes reported throughout the studies (n=541)**

| **Outcome** | **Frequency** |
| --- | --- |
| Ankle-Brachial Pressure Index | 146 |
| Primary Patency | 109 |
| 6-Minute Walk Test | 88 |
| Rutherford-Becker Classification | 86 |
| Walking Impairment Questionnaire | 81 |
| Target Lesion Revascularisation, TLR | 79 |
| Intermittent Claudication Distance (Also; Pain-free Walking Distance, Initial Claudication Distance, Claudication Onset Distance, Functional Claudication Distance and Asymptomatic Walking Distance) | 73 |
| Maximal Walking Distance (Also; Absolute Walking Distance) | 71 |
| Clinically Driven Target Lesion Revascularisation, CD-TLR | 62 |
| Major Adverse Events | 58 |
| All-cause Mortality | 53 |
| SF-36 | 52 |
| Major Adverse Limb Events | 46 |
| Major Amputation | 40 |
| EQ-5D-5L | 35 |
| Amputation | 31 |
| Technical Success | 30 |
| Claudication Onset Time (Also; Pain-free Walking Time) | 28 |
| Procedural Success | 27 |
| EQ-5D-3L | 27 |
| Major Adverse Cardiovascular Events, MACE | 24 |
| Death | 23 |
| Maximal Walking Time (Also; Peak Walking Time) | 21 |
| VascuQoL-6 | 20 |
| Restenosis | 20 |
| Binary Restenosis | 19 |
| Secondary Patency | 18 |
| Target Vessel Revascularisation, TVR | 18 |
| Myocardial Infarction | 16 |
| Cardiovascular Death | 15 |
| Stroke | 15 |
| Device Success | 14 |
| Limb Salvage | 14 |
| Primary Sustained Clinical Improvement | 14 |
| Late Lumen Loss | 13 |
| Target Limb Amputation | 13 |
| Mortality | 12 |
| Survival | 12 |
| Stent Fracture | 12 |
| Acute Limb Ischaemia | 11 |
| Clinically Driven Target Vessel Revascularisation, CD-TVR | 11 |
| Primary Assisted Patency | 11 |
| Major Bleeding | 10 |
| Adverse Events | 9 |
| Amputation-free Survival | 9 |
| Flow Mediated Arterial Dilatation | 9 |
| Walking Distance | 8 |
| Bleeding | 8 |
| Blood Pressure | 8 |
| Heart Rate | 8 |
| Clinical Success | 7 |
| Target Extremity Revascularisation, TER | 7 |
| Quality Adjusted Life Years | 9 |
| Peripheral Artery Questionnaire | 6 |
| SF-12 | 6 |
| TcPO2 | 6 |
| VascuQol-25 | 6 |
| C-Reactive Protein | 6 |
| In stent Restenosis | 6 |
| Peak VO2 | 6 |
| Toe-Brachial Pressure Index | 6 |
| Hospital Stay | 5 |
| Hospital Anxiety and Depression Scale | 5 |
| Incremental Cost-effectiveness Ratio | 5 |
| Perioperative All-cause Mortality | 5 |
| Stenosis | 5 |
| Distal Embolisation | 4 |
| Minor Amputation | 4 |
| Physical Activity | 4 |
| Reintervention | 4 |
| Unplanned Index Limb Revascularisation | 4 |
| Bail Out Stenting | 4 |
| Brachial Artery Flow-mediated Dilatation | 4 |
| Device and Procedure Related Mortality | 4 |
| Fontaine Classification | 4 |
| Haemodynamic Improvement | 4 |
| PADQOL | 4 |
| Serious Adverse Events | 4 |
| Target Lesion Restenosis | 4 |
| Feasibility | 4 |
| Clinical Improvement | 3 |
| Intermittent Claudication Questionnaire | 3 |
| Lipid Profile | 3 |
| Target Lesion Thrombosis | 3 |
| Assisted Primary Patency | 3 |
| Association of Paclitaxel Dose and Mortality | 3 |
| Borg's Rating of Perceived Exertion Scale | 3 |
| Clinical Benefit | 3 |
| Composite of Device, procedure and clinical success | 3 |
| Contrast Volume | 3 |
| Cost | 3 |
| Net Clinical Benefit | 3 |
| Non-Cardiovascular Death | 3 |
| Peri-procedural Complications | 3 |
| Repeated Chair Rise | 3 |
| Secondary Sustained Clinical Improvement | 3 |
| Short Physical Performance Battery | 3 |
| Thrombosis | 3 |
| Time Up and Go Test | 3 |
| 30 Seconds Sit to Stand Test | 2 |
| Intima-Media Thickness | 2 |
| Systemic Complications | 2 |
| Unilateral Heel Lift Test | 1 |
| Weight | 2 |
| Acute Kidney Injury | 2 |
| Adherence to Prescribed Medication | 2 |
| Ambulatory Ability | 2 |
| Arrythmias | 2 |
| Arterial Stiffness | 2 |
| Claudication Scale (CLAU-S) | 2 |
| Cutaneous Temperature of the Feet | 2 |
| Daily Physical Activity | 2 |
| Deep Vein Thrombosis | 2 |
| Dose Area Product | 2 |
| Event-free Survival | 2 |
| Fatal Bleeding/Critical Organ Bleeding | 2 |
| Fluoroscopy Time | 2 |
| Immediate Technical Success | 2 |
| In-hospital Mortality | 2 |
| In stent Thrombosis | 2 |
| Ischaemic Stroke/Transient Ischaemic Attack | 2 |
| Local Complications | 2 |
| Lower Extremity Revascularisation | 2 |
| MACCE | 2 |
| Minor Bleeding | 2 |
| Procedure Time | 2 |
| Progression to CLTI | 2 |
| Re-occlusion | 2 |
| Resting Metabolic Rate | 2 |
| Return to Operating Theatre | 2 |
| San Diego Claudication Questionnaire | 2 |
| Severe Limb Ischaemia | 2 |
| SF-12 V2 | 2 |
| Stenting Rate | 2 |
| Surgical Intervention | 2 |
| Systemic Embolic Events | 2 |
| Target Vessel Thrombosis | 2 |
| Theory Planned Behaviour Questionnaire | 2 |
| Tissue Oxygenation | 2 |
| Transient Ischaemic Attack | 2 |
| Upper Body Muscular Strength | 2 |
| Venous Thrombo-embolism | 2 |
| Vessel Dissection | 2 |
| Vessel Perforation | 2 |
| Walking Estimated-Limitation Calculated by History | 2 |
| Acceptability | 2 |
| Access Site Complication | 2 |
| Adherence To Exercise | 2 |
| Bailout Stenting | 2 |
| Embolisation | 2 |
| HLS-EU-Q16 | 2 |
| Major Target Limb Amputation | 2 |
| Perforation | 2 |
| Residual Stenosis | 2 |
| Time to CD-TLR | 2 |
| WHOQOL-Bref | 2 |
| 10 cm Visual Analog Scale of Pain | 1 |
| 10 Metre Walk Test | 1 |
| 30-day Device Related Complications | 1 |
| 4 Metre Walking Velocity | 1 |
| 4 Metres Walk Test | 1 |
| 5 Chair Sit Up Test | 1 |
| 5 Point Patient Safety Questionnaire | 1 |
| Absolute Change in Intermittent Claudication Distance | 1 |
| Acceleration Time | 1 |
| Accumulated Quality-adjusted Life | 1 |
| ACSM Higher Limit Recommendation for Physical Activity | 1 |
| ACSM Lower Limit Recommendation for Physical Activity | 1 |
| Action Planning and Action Control Scale | 1 |
| Active Slice Target to Background Ratio | 1 |
| Activity per Day | 1 |
| Acute Coronary Syndrome | 1 |
| Additional Interventions | 1 |
| Adverse Effects | 1 |
| Adherence to Rehabilitation | 1 |
| Air Kerma | 1 |
| Angiographic Success | 1 |
| Angiographically-documented Primary Patency | 1 |
| Angiography-driven Revascularisation | 1 |
| Antiplatelet Medication Assessments | 1 |
| Any Ipsilateral Intervention | 1 |
| Application Usage | 1 |
| Area Stenosis | 1 |
| Association of DCB Length and Mortality | 1 |
| Association of Patient and Angioplasty-based Variables on Failure of the Procedure | 1 |
| AUSVIQUOL | 1 |
| Average Duration of Stops | 1 |
| Average Hospital Stay | 1 |
| Average Mobility | 1 |
| Average Walking Speed | 1 |
| Brief Pain Inventory Score | 1 |
| Bleeding/Haematoma | 1 |
| Blood Flow and Vasodilator Function | 1 |
| Blood Flow at Rest after Reactive Hyperaemia | 1 |
| Brief Illness Perception Questionnaire | 1 |
| Brief International Physical Activity Questionnaire | 1 |
| By-pass Surgery of the Affected Limb | 1 |
| Calf Circumference | 1 |
| Calf Muscle Biopsy | 1 |
| Calf muscle biopsy measure of citrate synthase | 1 |
| Calf muscle biopsy measure of cytochrome-c oxidase | 1 |
| Calf muscle biopsy measure of peroxisome proliferator–activated receptor γ coactivator 1α | 1 |
| Calf muscle blood flow | 1 |
| Calf muscle haemoglobin oxygen saturation | 1 |
| Calf muscle oxygen extraction | 1 |
| Calf muscle oxygen saturation | 1 |
| Calf muscle oxygenation | 1 |
| Calf Muscle Perfusion | 1 |
| Candidate for Dual Pathway Inhibition | 1 |
| Capillary Perfusion Measured by MRI | 1 |
| Cardiac Death | 1 |
| Cardiac Output | 1 |
| Cause-specific Mortality | 1 |
| CD34+/133+ cell numbers in peripheral blood | 1 |
| CD-TER | 1 |
| Changes to the Treatment Plan caused by IVUS Findings | 1 |
| Chronic Limb Threatening Ischaemia | 1 |
| Cigarettes per Day | 1 |
| Claudication Pain on a Visual analogue Scale | 1 |
| Claudication Symptom Rating Scale | 1 |
| Clinical Failure | 1 |
| Clinical Status | 1 |
| Clinically-driven Stent Placement | 1 |
| Cold Sensation | 1 |
| Collateral Count | 1 |
| Composite of 1-year of Index Limb Amputation, Index Limb Reintervention and Index Limb-related Death | 1 |
| Composite of 30 Days Device and Procedure related Mortality, Major Target Limb Amputation and CD-TLR | 1 |
| Composite of 30-day Device malfunction, Device-related Serious events, and Other Adverse Events | 1 |
| Composite of ALI, Major Amputation, MI, Ischaemic Stroke, and CV Death | 1 |
| Composite of CV Death, Index Limb Amputation, Target Limb Thrombosis and CD-TLR | 1 |
| Composite of Death, TVR, and Amputation | 1 |
| Composite of Index Graft Occlusion/Revascularisation, Above Ankle Amputation and Death | 1 |
| Composite of Limb-related Death and Major Amputation | 1 |
| Composite of MI, Stroke and vessel or Stent Thrombosis | 1 |
| Composite of Patency and Freedom From CD-TLR | 1 |
| Composite of Sustained Decrease of 40% or more of eGFR, New ESRD, Renal or CV Death | 1 |
| Congestive Heart Failure | 1 |
| Coronary or Non-coronary Revascularisation Procedures | 1 |
| Coronary restenosis | 1 |
| Coronary Stent Thrombosis | 1 |
| Coronary, Carotid or Peripheral Revascularisation Procedures | 1 |
| Cost of Minimally Clinically Important Difference | 1 |
| Cost per Walking Metre Gained | 1 |
| Critical Limb Ischaemia | 1 |
| Critical Organ Bleeding | 1 |
| Cutaneous Microvascular Reactivity | 1 |
| Daily Ambulatory Activity | 1 |
| Daily Physical Activity (More than 10 Minutes) | 1 |
| Daily Step Count | 1 |
| Daily Walking Activity | 1 |
| DCB Success | 1 |
| Deep Infection | 1 |
| Dehiscence | 1 |
| Delta/variation of HHb During Occlusion | 1 |
| Delta/Variation of StO2 During Occlusion | 1 |
| Device and Procedure Related Complications | 1 |
| Device Fracture | 1 |
| Difference Between Redox Cycle Parameters | 1 |
| Disagreement in Imaging Findings Between Angiography and IVUS | 1 |
| Disease-specific Self-rated Health and Symptoms | 1 |
| Edinburgh Claudication Questionnaire | 1 |
| Endothelium-independent Dilatation of the Popliteal Artery | 1 |
| Ejection Fraction | 1 |
| Endothelial Cell Culture Bioassay | 1 |
| Exercise Compliance | 1 |
| Exercise Tolerance | 1 |
| Failed Angioplasty | 1 |
| Figure of 8 Walk Test | 1 |
| Final Residual Diameter Stenosis | 1 |
| Flexibility (YMCA sit-and-reach Test) | 1 |
| Flow Mediated Arterial Dilatation of Femoral Artery | 1 |
| Flow Mediated Dilatation of Popliteal Artery | 1 |
| Flow Mediated Vasodilatation and Pulse Wave Velocity | 1 |
| Fluorography | 1 |
| Fluoroscopy Dose | 1 |
| Foot Circumference | 1 |
| Forearm and Calf Blood Flow | 1 |
| Frequency of Analgesia Use | 1 |
| Gait Walking Variables | 1 |
| Gastrocnemius Mitochondrial Capacity and Blood Flow Measured by NIRS | 1 |
| Geographic Miss | 1 |
| Graft Revision | 1 |
| Groin Seroma | 1 |
| Haematoma | 1 |
| Healthcare Resource Use | 1 |
| Heart Rate Variation | 1 |
| High Frequency Power | 1 |
| Hospitalisation | 1 |
| HR, BP and HRV at rest in supine position | 1 |
| HR, BP and HRV during steady-state exercise | 1 |
| HR, BP and HRV during upright standing | 1 |
| Immediate Procedural Result | 1 |
| Immediate Procedural Success | 1 |
| In-hospital Bleeding | 1 |
| In-hospital Myocardial Infarction | 1 |
| International Physical Activity Questionnaire | 1 |
| Intracerebral Bleeding | 1 |
| Ischaemia | 1 |
| Ischaemic Stroke | 1 |
| Ischaemic Ulcer Time Course | 1 |
| Laser Doppler Perfusion Imaging | 1 |
| LDL-C Target | 1 |
| Leg Pain | 1 |
| Length of Stented Segment | 1 |
| Lesion Success | 1 |
| Lesion Diameter | 1 |
| Life Year Gained | 1 |
| Limb Infections | 1 |
| Limb-related Death | 1 |
| Liver Function | 1 |
| Low Frequency Power | 1 |
| Lower Extremity Blood Flow | 1 |
| Lymph Leak | 1 |
| Major Complication | 1 |
| Major Unplanned Amputation | 1 |
| Major Vascular Complications | 1 |
| Maximal Hyperaemic Response to Ischaemia | 1 |
| Maximum Blood Flow Velocity | 1 |
| Maximum Number of Steps per Day | 1 |
| Maximum T2 | 1 |
| McGill Pain Questionnaire | 1 |
| Mean Blood Flow velocity | 1 |
| Mean Cost per Patient | 1 |
| Mean Cumulative Cost per Patient | 1 |
| Mean Daily Step Count | 1 |
| Mean Number of Reinterventions | 1 |
| Mean Steps Walked per Period | 1 |
| Mean Time Spent per Day During Different Activities | 1 |
| Median Walking Distance | 1 |
| Minor Vascular Complication | 1 |
| Minutes of Exercise per Day | 1 |
| Modified Walking Impairment Questionnaire | 1 |
| Muscle Oxygenation | 1 |
| Nitric Oxide | 1 |
| Non-fatal Ischaemic Stroke | 1 |
| Non-fatal Myocardial Infarction | 1 |
| Non-major Bleeding | 1 |
| Non-thrombotic and Non-bleeding Adverse Events | 1 |
| Not Candidate for Dual Pathway Inhibition | 1 |
| Nottingham Extended Activities of Daily Living | 1 |
| Number of DSA | 1 |
| Number of Ischaemic Events Prevented | 1 |
| Number of Stops | 1 |
| OCT Identified Thrombus Burden | 1 |
| Overall Stent Placement | 1 |
| Oxidised LDL | 1 |
| Pain Control | 1 |
| Pain Numerical Scale | 1 |
| Pain Scale | 1 |
| Participant’s Options of Shoes | 1 |
| Peak Blood Flow | 1 |
| Peak Hyperaemic Popliteal Flow | 1 |
| Peak Leg Blood Flow | 1 |
| Peak Perfusion | 1 |
| Peak Reactive Hyperaemic Blood Flow | 1 |
| Perceived Pain Intensity | 1 |
| Percentage Diameter Stenosis | 1 |
| Percentage Plaque Volume | 1 |
| Perioperative Complications | 1 |
| Peripheral Artery Blood Flow and Shear Rate | 1 |
| Peripheral Artery Disease Risk Factors | 1 |
| Peripheral Revascularisation (Contralateral Limb) | 1 |
| Peripheral Revascularisation (Ipsilateral Limb) | 1 |
| Physical Examination | 1 |
| Plaque Modification | 1 |
| Plasma levels of ET-1, interleukin-6 (IL-6) and tumour necrosis factor-alpha (TNF-α) | 1 |
| Plasma Nitrate/Nitrite | 1 |
| Platelet Activation/Markers | 1 |
| Pneumonia | 1 |
| Popliteal Artery Blood Flow | 1 |
| Post-ischaemic Blood Flow | 1 |
| Post-occlusion Reactive Hyperaemia | 1 |
| Post-op Complications | 1 |
| Post-op Ventral Hernia | 1 |
| Power Output by Metabolic Equivalent of Task | 1 |
| Presence of Hypoechogenic (Halo) Surrounding the Stented Segment of the Artery | 1 |
| Procedural Complication | 1 |
| Progression-free Survival | 1 |
| PROMIS | 1 |
| Proportions of Circulating Angiogenic Cells | 1 |
| Proportions of TIE-2 Expressing Monocytes | 1 |
| Pseudoaneurysm | 1 |
| Pulmonary Oxygen Uptake | 1 |
| Pulse Wave Velocity | 1 |
| Puncture Site Complication | 1 |
| Puncture-site Bleeding requiring Intervention | 1 |
| Range of Foot Dorsiflexion | 1 |
| Range of Foot Plantarflexion | 1 |
| Rate of Use of Closure Device | 1 |
| Reactive Hyperaemia | 1 |
| Reactive Hyperaemia Index | 1 |
| Reactive Oxygen Species Production | 1 |
| Readmission | 1 |
| Recovery Time of StO2 After Occlusion | 1 |
| Recurrent Ischaemia-driven Revascularisation | 1 |
| Reduction in Ulcer Size | 1 |
| Reintervention for Thrombosis | 1 |
| Relative and Absolute number and Surface Markers of Mononuclear Cells | 1 |
| Relative Change in Flow | 1 |
| Relative Change in Frequency | 1 |
| Relief of Rest Pain | 1 |
| Renal Function | 1 |
| Reported Physical Activity | 1 |
| Resource Use Cost | 1 |
| Resting Blood Flow | 1 |
| Revascularisation | 1 |
| Revised Illness Perception Questionnaire | 1 |
| Risk Factors Associated with Cardiocerebrovascular Disease | 1 |
| Risk Factors for Progression of Clinical Failure | 1 |
| Run-off Status | 1 |
| Safety and Tolerance Profile of Sildenafil | 1 |
| Satisfaction (Using 0-100 analogue Scale) | 1 |
| Scaffold Thrombosis | 1 |
| Secondary Preventive Pharmacotherapy Intake | 1 |
| Sensitivity | 1 |
| Seroma/Haematoma | 1 |
| Serum ET-1 Concentration | 1 |
| Severe Angiographic Complications | 1 |
| SFA Mean Vessel Wall Area on MRI | 1 |
| Significant Embolic Event | 1 |
| Skeletal Muscle Microvascular Function | 1 |
| Smartphone App and FitBit Usage | 1 |
| Smoking | 1 |
| Smoking Cessation | 1 |
| Specificity | 1 |
| Spontaneous Baroreflex Sensitivity | 1 |
| Standing Balance | 1 |
| Stent Thrombosis | 1 |
| Stent-free DCB Patency | 1 |
| Steps per Day | 1 |
| Subjective Claudication Distance | 1 |
| Subjective Pain Sensation Score | 1 |
| Success of Drug Infusion | 1 |
| Successful Stent Implantation | 1 |
| Superficial Groin Wound Infection | 1 |
| Surgical Site Infection | 1 |
| Surgical Success | 1 |
| Symptomatic Acute Thrombosis | 1 |
| Symptomatic Venous Thrombo-embolism | 1 |
| Target to Background Ratio | 1 |
| Target Lesion Dissection | 1 |
| Thigh Circumference | 1 |
| Time of Pain Release Measured Using A Stopwatch | 1 |
| Time to Peak Perfusion | 1 |
| Time to Peak T2 | 1 |
| Time to Relief of Claudication | 1 |
| Tissue Deoxygenation | 1 |
| Tissue Oxygen Utility Capacity | 1 |
| Tissue Oxygenation Index | 1 |
| Total Blood Flow | 1 |
| Total Healthcare Cost | 1 |
| Total Power | 1 |
| Total Vascular Conductance | 1 |
| Treatment Emergent Adverse Events | 1 |
| Treatment Self-regulation Questionnaire | 1 |
| Ulcer Size | 1 |
| Unplanned Amputation | 1 |
| Unplanned Hospitalisation for a coronary or peripheral Vascular Event of Thrombotic Nature | 1 |
| Unplanned Major Amputation | 1 |
| Unscheduled Endovascular Therapy | 1 |
| Unstable Angina | 1 |
| UTI | 1 |
| Validated 10-Item Questionnaire | 1 |
| Vascular Complications | 1 |
| Vascular Death | 1 |
| Vascular Endothelial Growth Factor | 1 |
| Venous Oxygen Saturation | 1 |
| Vessel Compliance | 1 |
| Virus Shedding | 1 |
| Walking Autonomy | 1 |
| Walking Duration | 1 |
| Walking Speed | 1 |
| WHO/Rose Questionnaire | 1 |
| Whole Vessel Target to Background Ratio | 1 |
| Wound Healing Assessment | 1 |
| Wound Infection | 1 |
| Wound Surface Area | 1 |
| AUDIT-C Questionnaire | 1 |
| Barriers Specific Questionnaire | 1 |
| Barthel Questionnaire | 1 |
| Body Mass Index | 1 |
| Changes in Steps per Day | 1 |
| Chalder Fatigue Scale | 1 |
| Change in Calf Muscle Perfusion | 1 |
| Change in SFA Plaque Volume | 1 |
| Clinically Relevant Stenosis | 1 |
| Clinically Relevant Target Lesion Failure | 1 |
| Completion Rate | 1 |
| Composite of Acute Limb Ischaemia, Major Amputation, Myocardial Infarction, Ischaemic Stroke and Cardiovascular Death | 1 |
| Composite of Amputation and Mortality | 1 |
| Cost Effectiveness Ratio | 1 |
| Dialysis Dependence | 1 |
| Diastolic Blood Pressure | 1 |
| Dissection | 1 |
| eGFR | 1 |
| Endovascular Revascularisation Failure | 1 |
| Estimated Total Cost | 1 |
| Fatal Bleeding | 1 |
| GAD-7 Questionnaire | 1 |
| Gait Parameters | 1 |
| General Self-efficacy Scale | 1 |
| Geriatric Depression Scale (GDS-15) | 1 |
| Grip Strength | 1 |
| HbA1c | 1 |
| Healthcare Cost | 1 |
| Impaired Flow | 1 |
| Incremental Cost per Avoided Stenosis Relapse | 1 |
| Incremental Cost per QALYs Gained | 1 |
| Katz Scale | 1 |
| LDL | 1 |
| MACE-A | 1 |
| Medication Use | 1 |
| Muscle Thickness | 1 |
| NAD+ Abundance on Muscle Biopsy | 1 |
| Nicotine Dependence FTND | 1 |
| Nonfatal Intracranial Bleeding | 1 |
| Nonfatal Ischaemic Stroke | 1 |
| Nonfatal Myocardial Infarction | 1 |
| Number of Sitting Bouts | 1 |
| Number of Transitions from Sitting to Standing | 1 |
| Other Nonfatal Non-Intracranial Bleeding | 1 |
| PAM-13 Questionnaire | 1 |
| Patient-specific Functional Scale | 1 |
| Perioperative Death | 1 |
| PHQ-9 Questionnaire | 1 |
| Prediction of Contrast Associated AKI | 1 |
| Prevention of Contrast Associated AKI | 1 |
| Requiring Dialysis | 1 |
| SARC-F | 1 |
| Sick Pay Days | 1 |
| StO2 | 1 |
| Symptomatic Target Limb DVT | 1 |
| Systolic BP | 1 |
| Time Spent in an Upright Position | 1 |
| Time Spent within Stepping Cadence of More than 100 Steps per Minute | 1 |
| Time to Restenosis | 1 |
| Transverse View Vessel Area Loss Percentage | 1 |
| UACR | 1 |
| VCSS | 1 |
| Villalta Score | 1 |
| Willingness to Pay | 1 |

# **Table 4. Variations in definitions of Major Adverse Events (MAE)**

| **Definition of Major Adverse Events (MAE)** | **Frequency** |
| --- | --- |
| All cause death, target limb major amputation, TLR | 9 |
| Cardiovascular death, amputation above the ankle and CD-TLR | 5 |
| Death and target limb amputation | 4 |
| Major target limb amputation, myocardial infarction, stroke, and death | 4 |
| All-cause death, CD-TVR, major target limb amputation, and lesion thrombosis | 3 |
| Death from any cause, CD-TVR, target limb major amputation and thrombosis | 3 |
| Device and procedure-related mortality, major target limb amputation, CD-TLR | 3 |
| All-cause mortality, target limb major amputation, TLR, and CD-TLR | 2 |
| Cardiovascular death, index limb amputation, CD-TLR | 2 |
| Death from any cause, device or procedure related death, and major target limb amputation | 2 |
| Death, Index limb amputation, and CD-TLR | 2 |
| Death, major amputations and CD-TLR | 2 |
| Non-fatal MI, Non-fatal stroke, Cardiovascular death | 2 |
| Target limb-related death, amputation of the target limb, or reintervention of the target limb | 1 |
| MAE was considered any of the following events: death, clinically driven target lesion revascularization (CD-TLR), major amputation of the treated limb, symptomatic deep vein thrombosis (DVT), pulmonary embolism (PE), or procedure-related bleeding requiring any transfusion or surgery. | 1 |
| All-cause mortality, major amputation of the target extremity, target lesion thrombosis, TVR | 1 |
| All-cause mortality, TVR, TLR, target lesion thrombosis, and procedure- or device-related adverse events | 1 |
| Cardiovascular-related deaths, CD-TLR, perforations/dissections of grade C or greater that required intervention; symptomatic distal emboli and unplanned amputation | 1 |
| CD-TLR, unplanned or unavoidable major amputation of the index limb and death | 1 |
| Major Adverse Events (all-cause mortality, major target limb amputation, and thrombosis at the  target lesion site) | 1 |
| CD-TVR (at least 70% lesion stenosis or at least 50% with attendant symptoms), major unplanned amputation of the treated limb (resulting in a limb prosthesis), or all-cause mortality) | 1 |
| Death, amputation, CD-TLR, target-limb ischemia requiring surgical intervention or surgical vessel repair or the need for prolonged hospitalization | 1 |
| Death, TVR, amputation above the metatarsals | 1 |
| Death, TVR/TLR, and major amputation | 1 |
| Major adverse limb events were a composite of acute limb ischemia, major amputation, or urgent revascularisation (thrombolysis or other interventions for ischemia). | 1 |
| Freedom from SCS-P–related major adverse events | 1 |
| Mortality, unplanned amputation, TLR | 1 |
| Target limb amputation, cardiovascular death, and stroke | 1 |

# **Table 5. Variations in definitions of Primary Patency**

| **Definition of Primary Patency** | **Frequency** |
| --- | --- |
| Primary patency (<50% restenosis) | 16 |
| Primary patency (Absence of binary restenosis and TLR) | 12 |
| Primary patency (<50% stenosis from duplex ultrasonography (PSVR <2.0) or from arteriography when available.) | 11 |
| Primary patency (Absence of binary restenosis determined by a peak systolic velocity ratio ≥2.4 on duplex or >50% stenosis on digital subtraction angiography) | 11 |
| Primary patency (Defined as a PSV ratio ≤2.5 as assessed by DUS in the absence of TLR, amputation, and/or surgical bypass) | 8 |
| Primary patency (Doppler US peak systolic velocity ratio of ≥2.4 and freedom from clinically driven target lesion revascularization after the index procedure) | 8 |
| Primary patency (Composite of freedom from binary restenosis and CD-TLR) | 7 |
| Primary patency (Freedom from >50% restenosis as determined by either duplex ultrasound (PSVR <2.4) or DSA, and freedom from TLR.) | 6 |
| Primary patency (Freedom from clinically-driven TLR and Doppler ultrasound stenosis >50% [PSVR ≥ 2.5]) | 6 |
| Primary patency (No evidence of restenosis ≥50 % or occlusion within the study lesion or TLR) | 5 |
| Primary patency (Peak systolic velocity ratio (PSVR) ≤2.5 in the absence of TLR, bypass of the target lesion, or major amputation of the target limb) | 4 |
| Primary patency (PAD and with de novo, restenotic or reoccluded lesions in the infrainguinal arteries) | 3 |
| No any given definition in the studies (Protocols of the studies are not published or are not available | 3 |
| Primary patency (Freedom from CD-TLR and restenosis as determined by duplex-derived peak systolic velocity ratio (PSVR) ≤2.4) | 2 |
| Primary patency (Peak systolic velocity ratio (PSVR) ≤2.4 on duplex ultrasound in the absence of clinically-driven TLR or bypass of the target lesion) | 2 |
| Primary patency (Uninterrupted patency without TLR or restenosis) | 2 |
| Primary patency was defined as duplex ultrasound peak systolic velocity ratio of 2.0 in the absence of CD-TLR or bypass surgery. | 1 |
| Primary patency defined as the absence of target lesion restenosis (peak systolic velocity ratio ≤ 2.4 on colour-flow duplex ultrasound measuring) | 1 |
| Primary patency (peak systolic velocity ratio <2.4 by duplex ultrasound without clinically driven TLR in the absence of bailout stenting). | 1 |

| **Table 6. Complete list of included studies** | | | | | | | | | |
| --- | --- | --- | --- | --- | --- | --- | --- | --- | --- |
| Author | Type of Study | Sample Size | Male | Female | Age (mean) | Rutherford Stage | Intervention | Type of Intervention | Follow up |
| Yonemitsu 2015(1) | Prospective Cohort study | 12 | 10 | 2 | 65 | 2-6 | medication - phase I FGF-2 recombinant Sendai virus | Medication | 12 Months |
| Gupta 2015(2) | Prospective Randomised controlled trial | 83 | 47 | 36 | 67.5 | 2-3 | caffeine | Medication | 3 Months |
| Leicht 2015(3) | Prospective Cohort study | 31 | NA | NA | NA | 2-3 | supervised exercise | Exercise | 12 Months |
| Markovic 2015(4) | Prospective Randomised controlled trial | 47 | 36 | 11 | 65.7 | 1-2 | Kinesitherapy (exercise) and electrotherapy | Exercise | 3 Months |
| Janas 2020(5) | Retrospective Cohort study | 204 | 134 | 70 | 71.5 | 3-6 | atherectomy devices + acetylsalicylic acid and clopidogrel | Surgical Intervention | 14 Months |
| Cochrane 2020(6) | Prospective Randomised controlled trial | 14 | 12 | 2 | 73.9 | 1-2 | sing session of vibration therapy | Miscellaneous | NA |
| Park 2020(7) | Prospective Randomised controlled trial | 11 | 5 | 6 | 66 | 2-4 | Mitochondrial targeted antioxidant (MitoQ) | Medication | 14 Days |
| Djerf 2020(8) | Prospective Randomised controlled trial | 158 | 83 | 75 | 68 | 2-3 | Revascularisation | Surgical Intervention | 60 Months |
| Angle 2020(9) | Prospective Single-arm trial | 276 | 175 | 101 | 69 | 2-3 | Misago peripheral stent | Surgical Intervention | 36 Months |
| Janas 2020(10) | Retrospective Cohort study | 182 | 106 | 76 | 72 | 3-6 | Directional + rotational atherectomy devices | Surgical Intervention | 9 Months |
| Hageman 2020(11) | Prospective Randomised controlled trial | 78 | 51 | 27 | 68 | 1-3 | Intermittent vacuum therapy (IVT) | Miscellaneous | 2 Months |
| McDermott 2020(12) | Prospective Randomised controlled trial | 44 | 29 | 15 | 72 | 0-3 | Flavanol-rich cocoa beverage | Medication | 6 Months |
| Bjorkman 2018(13) | Prospective Randomised controlled trial | 46 | 29 | 17 | 68 | 2-6 | Paclitaxel eluting stent vs prosthetic graft by-pass | Surgical Intervention | 24 Months |
| Horie 2018(14) | Prospective Randomised controlled trial | 103 | 70 | 33 | 62 | 1-5 | granulocyte colony-stimulating factor (G-CSF)-mobilized PBMNC transplantation | Medication | 12 Months |
| Micari 2018(15) | Prospective Cohort study | 1406 | 953 | 453 | 69 | 2-4 | Drug-coated balloon | Surgical Intervention | 24 Months |
| Baumhakel 2018(16) | Prospective Single-arm trial | 29 | 21 | 8 | 68 | 2-6 | Scoring balloon (VascuTrak) followed by drug-coated balloon | Surgical Intervention | 12 Months |
| Razavi 2018(17) | Prospective Single-arm trial | 262 | NA | NA | NA | 2-4 | Adventitial drug delivery of dexamethasone | Medication | 12 Months |
| Steiner 2018(18) | Prospective Randomised controlled trial | 105 | 76 | 29 | 68 | 2-4 | Ranger Paclitaxel-Coated PTA Balloon | Surgical Intervention | 12 Months |
| Green 2018(19) | Prospective Randomised controlled trial | 30 | 18 | 12 | 66 | 1-3 | Extracorporeal shockwave therapy (ESWT) | Miscellaneous | 12 Months |
| McDermott 2018(20) | Prospective Randomised controlled trial | 200 | 95 | 105 | 70.2 | 0-3 | Home-based exercise intervention | Exercise | 9 Months |
| Soga 2018(21) | Prospective Randomised controlled trial | 200 | 131 | 69 | 73 | 2-4 | Cilostazol treatment | Medication | 36 Months |
| Albrecht 2018(22) | Prospective Randomised controlled trial | 153 | NA | NA | 68 | 2-4 | Paclitaxel-coated balloon catheter (SeQuent Please OTW) | Surgical Intervention | 24 Months |
| Moll 2018(23) | Prospective Randomised controlled trial | 203 | 145 | 58 | 67 | 2-5 | Edoxaban plus aspirin vs dual antiplatelet therapy | Medication | 6 Months |
| Yang 2018(24) | Prospective Randomised controlled trial | 18 | 10 | 8 | 67 | 3-6 | Ticagrelor and aspirin vs clopidogrel and aspirin | Medication | 6 Months |
| Lindgren 2018(25) | Prospective Randomised controlled trial | 95 | 46 | 49 | NA | 2-3 | Primary stenting with nitinol self-expanding stents | Surgical Intervention | 24 Months |
| Schneider 2018(26) | Prospective Randomised controlled trial | 331 | 218 | 113 | 68 | 2-4 | Drug-coated balloon (IN.PACT Admiral Paclitaxel-Coated Balloon Catheter) | Surgical Intervention | 36 Months |
| DavinsRiu 2018(27) | Prospective Randomised controlled trial | 150 | 136 | 14 | NA | 1-3 | CONTECI telehealth program | Exercise | 12 Months |
| Laird 2018(28) | Prospective Randomised controlled trial | 267 | 190 | 77 | 67 | 2-4 | TIGRIS novel nitinol stent | Surgical Intervention | 24 Months |
| Iida 2018(29) | Prospective Randomised controlled trial | 100 | 76 | 24 | 74 | 2-4 | Drug-coated balloon | Surgical Intervention | 12 Months |
| Rastan 2018(30) | Prospective Single-arm trial | 158 | 82 | 76 | 72 | 1-6 | Directional atherectomy | Surgical Intervention | 12 Months |
| Lichtenberg 2018(31) | Prospective Cohort study | 172 | 107 | 65 | 71 | 2-5 | Ranger paclitaxel-coated balloon catheter | Surgical Intervention | 24 Months |
| Harwood 2018(32) | Prospective Randomised controlled trial | 30 | 18 | 12 | 66 | 2-3 | Extracorporeal shockwave therapy | Miscellaneous | 3 Months |
| Deloge 2018(33) | Prospective Cohort study | 53 | 36 | 17 | 68 | 2-3 | Paclitaxel-coated balloon | Surgical Intervention | 12 Months |
| Normahani 2018(34) | Prospective Randomised controlled trial | 37 | 30 | 7 | 69 | 1-3 | Wearable activity monitors (WAM) | Exercise | 12 Months |
| Tew 2017(35) | Prospective Randomised controlled trial | 34 | 27 | 7 | 68 | 2-3 | Unloading ("rocker-soled") shoes | Exercise | 14 Days |
| McDermott 2017(36) | Prospective Randomised controlled trial | 210 | 128 | 82 | 67 | 1-3 | Granulocyte--macrophage colony-stimulating factor with or without supervised exercise | Medication | 6 Months |
| Muller-Hulsbeck 2017(37) | Prospective Cohort study | 57 | 47 | 10 | 69 | 2-4 | Eluvia drug-eluting vascular stent system; acetylsalicylic acid (ASA) after 60 days | Surgical Intervention | 36 Months |
| Park 2020(38) | Prospective Randomised controlled trial | 53 | NA | 53 | 65 | 1-4 | Treadmill walking vs walking in heated water | Exercise | 3 Months |
| Babber 2020(39) | Prospective Randomised controlled trial | 42 | NA | NA | NA | 1-3 | Footplate neuromuscular electrical stimulation | Miscellaneous | 2 Months |
| Park 2019(40) | Prospective Randomised controlled trial | 72 | NA | 72 | 71 | 1-4 | Aquatic walking training | Exercise | 3 Months |
| Brenner 2020(41) | Prospective Randomised controlled trial | 33 | 21 | 12 | 68 | 1-3 | Progressive walking program | Exercise | 3 Months |
| Murrow 2019(42) | Prospective Randomised controlled trial | 18 | 14 | 4 | 72 | 1-3 | Near infrared spectroscopy-guided exercise training | Exercise | 3 Months |
| Teichgraber 2020(43) | Prospective Randomised controlled trial | 171 | 111 | 60 | 69 | 2-5 | Drug-coated balloon | Surgical Intervention | 24 Months |
| Bosiers 2020(44) | Prospective Randomised controlled trial | 220 | 159 | 61 | 69 | 2-5 | ZILVER PTX stent | Surgical Intervention | 24 Months |
| Golzar 2020(45) | Prospective Cohort study | 50 | 32 | 18 | 68 | 2-4 | Paclitaxel-Eluting stent | Surgical Intervention | 12 Months |
| GaleaHolmes 2019(46) | Prospective Randomised controlled trial | 24 | 19 | 5 | 67 | 2-3 | MOSAIC Walking behavior-change intervention | Exercise | 4 Months |
| Hotta 2019(47) | Prospective Randomised controlled trial | 13 | 7 | 6 | 71 | 1-3 | Muscle stretching | Exercise | 30 Days |
| Chen 2019(48) | Prospective Cohort study | 143 | 107 | 36 | 67 | 2-4 | Drug-coated balloon | Surgical Intervention | 12 Months |
| Hammer 2019(49) | Prospective Randomised controlled trial | 70 | 55 | 15 | NA | 2-3 | n-3 PUFA | Medication | 3 Months |
| Dagenais 2019(50) | Prospective Randomised controlled trial | 12090 | 7839 | 4251 | 63.5 | 0-6 | Insulin glargine daily injection or omega-3 supplement | Medication | 74 Months |
| Gardner 2019(51) | Prospective Randomised controlled trial | 114 | 62 | 52 | 65 | 1-3 | Home-based or supervised exercise rehabilitation program | Exercise | 3 Months |
| Liistro 2019(52) | Prospective Randomised controlled trial | 192 | 122 | 70 | 74 | 3-6 | Drug-eluting balloon vs drug-eluting stent | Surgical Intervention | 12 Months |
| Laird 2019(53) | Prospective Randomised controlled trial | 331 | 218 | 113 | 68 | 2-5 | Paclitaxel-coated balloon | Surgical Intervention | 60 Months |
| Imran 2019(54) | Retrospective Cohort study | 70 | 46 | 24 | 71 | 2-6 | Drug-coated balloon | Surgical Intervention | 12 Months |
| Omarjee 2019(55) | Prospective Randomised controlled trial | 12 | 8 | 4 | NA | 1-3 | Single dose of oral sildenafil | Medication | 7 Days |
| Tepe 2019(56) | Prospective Cohort study | 126 | 87 | 39 | 68 | 2-4 | Paclitaxel-coated balloon | Surgical Intervention | 12 Months |
| Stabile 2019(57) | Prospective Cohort study | 139 | 109 | 30 | 67 | 2-5 | Legflow drug-coated balloon | Surgical Intervention | 12 Months |
| SanNorberto 2020(58) | Prospective Single-arm trial | 46 | 42 | 4 | 72 | 2-6 | Supera stent | Surgical Intervention | 36 Months |
| Khalili 2019(59) | Retrospective Cohort study | 518 | 409 | 109 | 67 | 1-6 | Atherectomy | Surgical Intervention | 12 Months |
| Akerman 2019(60) | Prospective Randomised controlled trial | 22 | 15 | 7 | 75 | 1-2 | Heat therapy vs supervised exercise therapy | Exercise | 3 Months |
| Balin 2019(61) | Prospective Randomised controlled trial | 63 | 46 | 17 | 63 | 1-3 | Remote ischemic preconditioning (RIPC) | Miscellaneous | 30 Days |
| Novakovic 2019(62) | Prospective Randomised controlled trial | 29 | 21 | 8 | 64 | 1-3 | Moderate-pain vs pain-free exercise training | Exercise | 3 Months |
| Bausback 2019(63) | Prospective Randomised controlled trial | 150 | 102 | 48 | 69 | 2-5 | Drug-eluting stent vs drug-coated balloon | Surgical Intervention | 36 Months |
| Russell 2019(64) | Prospective Randomised controlled trial | 38 | 27 | 11 | 65 | 1-3 | Subcutaneous canakinumab | Medication | 12 Months |
| Tenore 2019(65) | Prospective Randomised controlled trial | 180 | 130 | 50 | 71 | 1-3 | Annurca apple polyphenolic extract | Medication | 6 Months |
| Pellinger 2019(66) | Prospective Randomised controlled trial | 6 | 5 | 1 | 69 | 1-3 | Acute lower leg heating | Miscellaneous | 7 Days |
| Iida 2019(67) | Prospective Randomised controlled trial | 100 | 76 | 24 | 74 | 2-4 | Drug-coated balloon (IN.PACT Admiral MDT-2113) | Surgical Intervention | 24 Months |
| McDermott 2019(68) | Prospective Randomised controlled trial | 205 | 125 | 80 | 67 | 1-3 | Granulocyte--macrophage colony-stimulating factor with or without supervised exercise | Medication | 6 Months |
| McDermott 2019(69) | Prospective Randomised controlled trial | 156 | 75 | 81 | 71 | 0-3 | Supervised treadmill exercise or supervised lower-extremity resistance training | Exercise | 12 Months |
| Schroe 2018(70) | Prospective Cohort study | 371 | 271 | 100 | 68 | 2-4 | Stellarex drug-coated balloon | Surgical Intervention | 12 Months |
| Gardner 2018(71) | Prospective Randomised controlled trial | 180 | 95 | 85 | 66 | 1-3 | Home-based exercise or supervised exercise rehabilitation program | Exercise | 3 Months |
| Jakubseviciene 2019(72) | Prospective Randomised controlled trial | 160 | 149 | 11 | 68 | 1-4 | Individual long-term exercise program | Exercise | 6 Months |
| Collins 2019(73) | Prospective Randomised controlled trial | 174 | 46 | 128 | 64 | 1-3 | motivational interviewing (MI) | Miscellaneous | 12 Months |
| Perin 2017(74) | Prospective Randomised controlled trial | 78 | 57 | 21 | 66 | 2-3 | ALDH Bright Cell therapy | Medication | 6 Months |
| Jia 2016(75) | Prospective Randomised controlled trial | 200 | 147 | 53 | 66 | 2-5 | Paclitaxel-coated balloon | Surgical Intervention | 12 Months |
| Ellul 2016(76) | Prospective Cohort study | 40 | 30 | 10 | 71 | 1-3 | Transcutaneous calf-muscle stimulation | Miscellaneous | 3 Months |
| Harzand 2020(77) | Prospective Randomised controlled trial | 15 | 6 | 9 | 66 | 1-3 | Smartphone-enabled, home-based exercise program | Exercise | 12 Months |
| Woessner 2018(78) | Prospective Randomised controlled trial | 24 | 15 | 9 | 70 | 1-3 | Exercise rehabilitation program plus inorganic nitrate | Medication | 3 Months |
| Dopheide 2016(79) | Prospective Cohort study | 60 | 42 | 18 | 69 | 1-3 | Supervised vs non-supervised exercise training | Exercise | 7 Months |
| Kohi 2020(80) | Prospective Randomised controlled trial | 331 | 218 | 113 | 68 | 2-4 | IN.PACT drug-coated balloon | Surgical Intervention | 36 Months |
| Bonaca 2020(81) | Prospective Randomised controlled trial | 17160 | NA | NA | NA | 0-6 | Dapaglifozin | Medication | 48 Months |
| Rodriguez-Leyva 2019(82) | Prospective Randomised controlled trial | 83 | 62 | 21 | 66 | 1-3 | 30g of milled flaxseed per day | Medication | 12 Months |
| Steiner 2020(83) | Prospective Randomised controlled trial | 414 | 260 | 154 | 68 | 2-4 | Drug-coated balloon with either high-dose or low-dose paclitaxel coating | Surgical Intervention | 60 Months |
| Zeller 2020(84) | Prospective Single-arm trial | 50 | 29 | 21 | 70 | 2-4 | Drug-eluting balloon | Surgical Intervention | 6 Months |
| Monteiro 2019(85) | Prospective Randomised controlled trial | 40 | 28 | 12 | 64 | 1-3 | Modified vs conventional aerobic training | Exercise | 3 Months |
| Lai 2020(86) | Prospective Single-arm trial | 44 | 33 | 11 | 68 | 1-5 | Drug-coated balloon | Surgical Intervention | 12 Months |
| Atkin 2019(87) | Prospective Single-arm trial | 34 | 30 | 4 | 68 | 1-3 | Cycloidal vibration therapy | Miscellaneous | 3 Months |
| Laird 2019(88) | Prospective Randomised controlled trial | 331 | 218 | 115 | 68 | 2-4 | Drug-coated balloon | Surgical Intervention | 60 Months |
| Kostewicz 2019(89) | Prospective Randomised controlled trial | 30 | 21 | 9 | 67 | 2-3 | March field training | Exercise | 3 Months |
| Bock 2018(90) | Prospective Randomised controlled trial | 21 | 12 | 9 | 72 | 0-1 | Inorganic nitrate | Medication | 2 Months |
| Bunte 2018(91) | Prospective Single-arm trial | 250 | 154 | 96 | 68 | 2-4 | Nitinol self-expanding stent | Surgical Intervention | 36 Months |
| Bronas 2018(92) | Prospective Single-arm trial | 14 | 11 | 3 | 66 | 1-3 | Rhythmic auditory music stimulaton | Miscellaneous | 7 Days |
| Miura 2018(93) | Prospective Randomised controlled trial | 255 | 172 | 83 | 73 | 2-4 | Drug-eluting versus bare-metal stent with or without cilostazol | Surgical Intervention | 12 Months |
| Ali 2017(94) | Prospective Randomised controlled trial | 76 | 64 | 12 | 56 | 1-3 | Prostaglandin E1 | Medication | 30 Days |
| Schroeder 2017(95) | Prospective Cohort study | 80 | 60 | 20 | 68 | 2-4 | Paclitaxel-coated balloon with or without predilatation | Surgical Intervention | 24 Months |
| Garcia 2017(96) | Prospective Single-arm trial | 799 | 436 | 363 | 70 | 1-6 | Directional atherectomy | Surgical Intervention | 12 Months |
| Janas 2017(97) | Retrospective Cohort study | 419 | 256 | 163 | 69 | 3-6 | Arterial interventions with atherectomy vs balloon angioplasty | Surgical Intervention | 24 Months |
| Laird 2015(98) | Prospective Randomised controlled trial | 331 | 218 | 115 | 68 | 2-4 | Drug-coated balloon | Surgical Intervention | 24 Months |
| DeHaro 2016(99) | Prospective Randomised controlled trial | 56 | 56 | NA | 58 | 1-2 | Bosentan | Medication | 6 Months |
| Kinstner 2016(100) | Prospective Randomised controlled trial | 74 | 43 | 31 | 68 | 2-3 | Paclitaxel-eluting balloon | Surgical Intervention | 12 Months |
| Seenan 2016(101) | Prospective Randomised controlled trial | 36 | 29 | 7 | 70 | 1-3 | Transcutaneous electrical nerve stimulation | Miscellaneous | NA |
| Lammer 2016(102) | Prospective Single-arm trial | 35 | 27 | 8 | 65 | 1-3 | Bioresorbable everolimus-eluting vascular scaffold | Surgical Intervention | 24 Months |
| Nordanstig 2016(103) | Prospective Randomised controlled trial | 158 | 83 | 75 | 68 | 1-2 | Revascularization | Surgical Intervention | 24 Months |
| Muller-Hulsbeck 2016(104) | Prospective Single-arm trial | 57 | 47 | 10 | 69 | 2-4 | Paclitaxel-eluting stent | Surgical Intervention | 12 Months |
| Scheinert 2016(105) | Prospective Randomised controlled trial | 126 | 79 | 47 | 67 | 2-4 | Drug-coated balloon | Surgical Intervention | 12 Months |
| DeHaro 2019(106) | Prospective Randomised controlled trial | 56 | 56 | NA | 58 | 1-2 | Bosentan | Medication | 48 Months |
| Ahmed 2019(107) | Prospective Randomised controlled trial | 45 | 33 | 12 | 65 | 1-2 | Remote ischaemic pre-conditionin | Miscellaneous | 30 Days |
| Jordan 2019(108) | Prospective Randomised controlled trial | 31 | 25 | 6 | 69 | 1-2 | Three-curved rocker-soled shoes | Exercise | NA |
| Xu 2018(109) | Prospective Randomised controlled trial | 200 | 147 | 53 | 66 | 0-6 | Drug-coated balloon | Surgical Intervention | 24 Months |
| Scheinert 2018(110) | Prospective Single-arm trial | 157 | 104 | 53 | 70 | 2-4 | Drug-coated balloon | Surgical Intervention | 12 Months |
| Kropielnicka 2018(111) | Prospective Cohort study | 80 | NA | NA | 67 | 1-3 | Walking training | Exercise | 12 Months |
| Ansel 2018(112) | Retrospective Cohort study | 1125 | 775 | 350 | 69 | 2-4 | Drug-coated balloon | Surgical Intervention | 12 Months |
| Jeon-Slaughter 2018(113) | Retrospective Cohort study | 969 | NA | NA | NA | 1-6 | Femoropopliteal stent | Surgical Intervention | 12 Months |
| Gray 2018(114) | Prospective Randomised controlled trial | 465 | 308 | 157 | 68 | 2-4 | Drug-coated balloon | Surgical Intervention | 12 Months |
| Afzelius 2018(115) | Prospective Randomised controlled trial | 48 | 27 | 21 | 70 | 1-2 | Intermittent vacuum therapy (IVT) | Miscellaneous | 3 Months |
| Bock 2018(116) | Prospective Randomised controlled trial | 21 | 12 | 9 | 72 | 0-1 | Sodium nitrate | Medication | 2 Months |
| Duscha 2018(117) | Prospective Randomised controlled trial | 19 | 16 | 3 | 69 | 1-3 | Mobile Health Program (mHealth) | Exercise | 3 Months |
| Lindeman 2018(118) | Prospective Randomised controlled trial | 54 | 30 | 24 | 58 | 2-4 | Bone Marrow-derived Mononuclear Cells | Medication | 12 Months |
| Deev 2018(119) | Prospective Randomised controlled trial | 48 | 32 | 16 | 64 | 1-4 | pl-VEGF165 gene therapy | Medication | 60 Months |
| Zeller 2017(120) | Prospective Randomised controlled trial | 102 | 68 | 34 | 70 | 2-4 | Directional atherectomy followed by paclitaxel-coated balloon | Surgical Intervention | 12 Months |
| Krankenberg 2017(121) | Prospective Randomised controlled trial | 660 | 497 | 163 | 64 | 1-4 | Self-expanding vs balloon-expandable stents | Surgical Intervention | 12 Months |
| deBoer 2017(122) | Prospective Randomised controlled trial | 160 | 102 | 58 | 67 | 2-6 | Paclitaxel-eluting balloon with supera stenting vs supera stenting alone | Surgical Intervention | 12 Months |
| Ott 2017(123) | Prospective Randomised controlled trial | 70 | 48 | 22 | 69 | 2-5 | Paclitaxel-eluting balloon | Surgical Intervention | 24 Months |
| Krishnan 2017(124) | Prospective Randomised controlled trial | 300 | 176 | 124 | 69 | 2-4 | Drug-coated balloon | Surgical Intervention | 12 Months |
| Tepe 2017(125) | Prospective Randomised controlled trial | 153 | 104 | 49 | 68 | 2-4 | Drug-coated balloon | Surgical Intervention | 24 Months |
| Embrey 2017(126) | Prospective Randomised controlled trial | 27 | NA | NA | 68 | 1-3 | Functional electrical stimulation | Miscellaneous | 2 Months |
| vanSchaardenburgh 2017(127) | Prospective Randomised controlled trial | 28 | 15 | 13 | 69 | 1-3 | Exercise program | Exercise | 2 Months |
| Labrunee 2015(128) | Prospective Single-arm trial | 15 | 13 | 2 | 58 | 1-3 | Transcutaneous electrical nerve stimulation | Miscellaneous | NA |
| Rastan 2015(129) | Prospective Randomised controlled trial | 246 | 158 | 88 | 72 | 2-5 | Stent placement vs balloon angioplasty | Surgical Intervention | 24 Months |
| Scheinert 2015(130) | Prospective Randomised controlled trial | 60 | 34 | 26 | 71 | 2-5 | Paclitaxel-releasing balloon using a BTHC excipient | Surgical Intervention | 12 Months |
| Dopheide 2015(131) | Prospective Single-arm trial | 40 | NA | NA | NA | 1-3 | Home-based exercise training | Exercise | 12 Months |
| Murphy 2015(132) | Prospective Randomised controlled trial | 111 | 69 | 42 | 64 | 2-3 | Supervised exercise vs stent revascularization vs optimal medical care | Exercise | 18 Months |
| Mays 2015(133) | Prospective Randomised controlled trial | 20 | 16 | 4 | 66 | 2-3 | Community-based walking exercise | Exercise | 3 Months |
| Schroeder 2015(134) | Prospective Single-arm trial | 50 | 31 | 19 | 69 | 2-4 | Drug-coated balloon | Surgical Intervention | 24 Months |
| Rocha-Singh 2015(135) | Prospective Single-arm trial | 287 | 190 | 97 | 68 | 2-4 | Self-expanding peripheral stent | Surgical Intervention | 36 Months |
| Guirro 2015(136) | Prospective Randomised controlled trial | 15 | NA | 15 | 78 | 1-2 | Electrical stimulation, diathermy, physical exercise | Miscellaneous | 7 Days |
| Arao 2017(137) | Prospective Cohort study | 75 | 58 | 17 | 70 | 1-4 | Pitavastatin | Medication | 6 Months |
| Schroeder 2017(138) | Prospective Randomised controlled trial | 292 | 209 | 83 | 68 | 2-4 | Paclitaxel-coated balloon | Surgical Intervention | 12 Months |
| Ott 2017(139) | Prospective Randomised controlled trial | 155 | 111 | 44 | 69 | 2-6 | Paclitaxel-Eluting Balloon and Stenting Versus Plain Balloon Plus Stenting Versus Directional Atherectomy | Surgical Intervention | 24 Months |
| McDermott 2017(140) | Prospective Randomised controlled trial | 66 | 45 | 21 | 74 | 0-3 | Resveratrol | Medication | 6 Months |
| Chehuen 2017(141) | Prospective Randomised controlled trial | 42 | 42 | NA | 63 | 1-3 | Walking training | Exercise | 3 Months |
| Lindgren 2017(142) | Prospective Randomised controlled trial | 94 | 50 | 44 | 70 | 2-3 | Primary stenting with nitinol self expanding stents | Surgical Intervention | 12 Months |
| Kashyap 2017(143) | Prospective Single-arm trial | 22 | 10 | 12 | 62 | 1-5 | L-arginine | Exercise | 6 Months |
| Oakley 2017(144) | Prospective Cohort study | 29 | 16 | 13 | 67 | 2-3 | Augmented home exercise programme | Exercise | 12 Months |
| VanSchaardenburgh 2017(145) | Prospective Randomised controlled trial | 29 | 16 | 13 | 68 | 1-3 | Calf raise exercise vs traditional walking exercise | Exercise | 2 Months |
| Zen 2017(146) | Prospective Randomised controlled trial | 475 | 334 | 141 | 73 | 1-6 | Paclitaxel-eluting stent | Surgical Intervention | 12 Months |
| Falkowski 2020(147) | Prospective Randomised controlled trial | 256 | 162 | 94 | 66 | 2-5 | Paclitaxel-coated stent vs bare metal stent | Surgical Intervention | 36 Months |
| Branch 2019(148) | Prospective Randomised controlled trial | 27207 | 21244 | 5963 | 68 | 1-6 | Rivaroxaban and aspirin | Medication | 30 Months |
| Villemur 2020(149) | Prospective Randomised controlled trial | 38 | 31 | 7 | 65 | 1-3 | Interval training with active recovery (ITAR) | Exercise | 30 Days |
| Baker 2017(150) | Prospective Randomised controlled trial | 64 | 41 | 23 | 67 | 2-3 | Supervised exercise training | Exercise | 3 Months |
| Davis 2017(151) | Prospective Single-arm trial | 128 | 76 | 52 | 72 | 2-5 | Atherectomy | Surgical Intervention | 6 Months |
| Cavalcante 2017(152) | Prospective Randomised controlled trial | 11 | 11 | NA | NA | 1-3 | Arm crank exercise | Exercise | NA |
| Thomas 2017(153) | Prospective Randomised controlled trial | 21 | 15 | 6 | 71 | 1-2 | Hot-water immersion | Miscellaneous | NA |
| Hiatt 2017(154) | Prospective Randomised controlled trial | 13885 | 9997 | 3888 | 66 | 0-6 | Ticagrelor vs clopidogrel | Medication | 30 Months |
| Cavalcante 2017(155) | Prospective Cohort study | 18 | 14 | 4 | 65 | 1-2 | Graduated compression stocking | Miscellaneous | NA |
| Bague 2017(156) | Prospective Cohort study | 53 | 42 | 11 | 69 | 1-5 | Paclitaxel eluting balloon | Surgical Intervention | 18 Months |
| Mazari 2017(157) | Prospective Randomised controlled trial | 178 | NA | NA | 75 | 2-3 | supervised exercise, percutaneous transluminal angioplasty or combined treatment | Exercise | 12 Months |
| Szymczak 2016(158) | Prospective Randomised controlled trial | 50 | 23 | 27 | 68 | 1-3 | Walking exercise vs resistance training | Exercise | 3 Months |
| Schmidt 2016(159) | Retrospective Cohort study | 260 | 164 | 96 | 68 | 1-6 | Drug-coated balloon | Surgical Intervention | 24 Months |
| Dake 2016(160) | Prospective Randomised controlled trial | 474 | 307 | 167 | 68 | 2-6 | Paclitaxel-eluting stent | Surgical Intervention | 60 Months |
| Yokoi 2016(161) | Prospective Single-arm trial | 907 | 638 | 269 | 73.5 | 2-6 | Paclitaxel eluting stent | Surgical Intervention | 12 Months |
| Chu 2016(162) | Prospective Randomised controlled trial | 72 | 49 | 23 | 59 | 2-3 | Trimetazidine hydrochloride | Medication | 6 Months |
| Katsanos 2016(163) | Prospective Randomised controlled trial | 200 | 142 | 58 | 73 | 3-5 | Infrapopliteal Balloon Angioplasty Versus Sirolimus-Eluting Stenting | Surgical Intervention | 12 Months |
| DeHaro 2016(164) | Prospective Randomised controlled trial | 56 | 56 | NA | 58 | 1-2 | Bosentan | Medication | 12 Months |
| Lamberti 2016(165) | Prospective Randomised controlled trial | 27 | 21 | 6 | 68 | 2-3 | Home-based exercise program | Exercise | 4 Months |
| Han 2016(166) | Prospective Single-arm trial | 287 | 190 | 97 | NA | 2-3 | Femoropopliteal stent | Surgical Intervention | 36 Months |
| Grossman 2016(167) | Prospective Cohort study | 12 | 12 | NA | 60 | 2-3 | Multi-gene cell therapy | Medication | 12 Months |
| Delaney 2016(168) | Prospective Randomised controlled trial | 35 | 25 | 10 | 71 | 1-3 | Supervised exercise training | Exercise | 3 Months |
| Bulinska 2016(169) | Prospective Randomised controlled trial | 52 | 37 | 15 | 68 | 1-2 | Nordic pole walking | Exercise | 3 Months |
| Robertson 2016(170) | Prospective Randomised controlled trial | 50 | 39 | 11 | 68 | 1-3 | Allopurinol | Medication | 6 Months |
| Dipnarine 2016(171) | Prospective Single-arm trial | 26 | 17 | 9 | 67.58 | 1-3 | supervised exercise training | Exercise | 3 Months |
| Mauer 2015(172) | Prospective Randomised controlled trial | 23 | 11 | 12 | 67 | 1-3 | supervised exercise program | Exercise | 3 Months |
| Alvarez 2015(173) | Prospective Randomised controlled trial | 34 | NA | NA | NA | 1-6 | High-pressure, Intermittent Pneumatic Compression | Miscellaneous | 4 Months |
| Fakhry 2015(174) | Prospective Randomised controlled trial | 212 | 132 | 80 | 65 | 1-3 | supervised exercise and endovascualr revascularisation | Exercise | 12 Months |
| Zeller 2015(175) | Prospective Randomised controlled trial | 72 | 57 | 15 | 71 | 2-5 | Paclitaxel coated balloon | Surgical Intervention | 12 Months |
| Babaev 2015(176) | Prospective Single-arm trial | 25 | 19 | 6 | 70.4 | 3 | orbital atherectomy system | Surgical Intervention | 12 Months |
| Rastan 2015(177) | Prospective Single-arm trial | 145 | NA | NA | NA | 1-6 | Directional Atherectomy | Surgical Intervention | 12 Months |
| Gommans 2015(178) | Prospective Randomised controlled trial | 169 | 113 | 56 | 66 | 1-3 | Walking advice vs SET with feedback vs SET without feedback | Exercise | 12 Months |
| Schulte 2015(179) | Prospective Randomised controlled trial | 92 | 62 | 30 | 72.9 | 2-5 | self-expanding nitinol stent | Surgical Intervention | 12 Months |
| daSilva 2015(180) | Prospective Randomised controlled trial | 10 | 10 | NA | 62 | 1-2 | NAC | Medication | 5 Days |
| Rosenfield 2015(181) | Prospective Randomised controlled trial | 476 | 300 | 176 | 68.2 | 2-4 | angioplasty with a paclitaxel-coated balloon | Surgical Intervention | 12 Months |
| Delagarde 2015(182) | Prospective Randomised controlled trial | 20 | 17 | 3 | 63.4 | 1-3 | Remote ischemic preconditioning (RIPC) | Miscellaneous | 7 Days |
| McDermott 2015(183) | Prospective Randomised controlled trial | 178 | 88 | 90 | 70 | 1-3 | unsupervised home based exercise | Exercise | 12 Months |
| Garcia 2015(184) | Prospective Single-arm trial | 264 | 168 | 96 | 68.7 | 2-4 | nitinol stent | Surgical Intervention | 12 Months |
| Gernigon 2015(185) | Prospective Cohort study | 83 | 78 | 5 | 64 | 1-3 | walking activity post-stent vs supervised exericse | Exercise | 6 Months |
| Tew 2015(186) | Prospective Randomised controlled trial | 23 | 16 | 7 | 68 | 1-3 | Structured, home-based exercise programmes that promote self-managed walking | Exercise | 2 Months |
| Dippel 2015(187) | Prospective Randomised controlled trial | 250 | NA | NA | 62 | 1-4 | excimer laser atherectomy + PTA | Surgical Intervention | 12 Months |
| Fokkenrood 2015(188) | Prospective Single-arm trial | 41 | 20 | 21 | 69 | 1-3 | 3 month supervised exercise therapy | Exercise | 3 Months |
| Tepe 2015(189) | Prospective Randomised controlled trial | 331 | 218 | 113 | 68 | 2-4 | paclitaxel-coated balloon | Surgical Intervention | 12 Months |
| Lammer 2015(190) | Prospective Randomised controlled trial | 141 | 100 | 41 | 69 | 2-5 | heparin-bonded covered stents | Surgical Intervention | 24 Months |
| Gray 2015(191) | Prospective Single-arm trial | 250 | 154 | 96 | 68 | 2-4 | SMART nitinol self expanding stent | Surgical Intervention | 12 Months |
| Bo 2015(192) | Prospective Randomised controlled trial | 50 | 24 | 26 | 67 | 1-3 | Percutaneous transluminal angioplasty + 12 week SET | Surgical Intervention | 3 Months |
| Vlajinac 2015(193) | Prospective Cohort study | 78 | 61 | 17 | NA | 2-5 | (aortobifemoral bypass | Surgical Intervention | 12 Months |
| Delaney 2015(194) | Prospective Randomised controlled trial | 35 | 25 | 9 | 71 | 1-3 | Supervised exercise training | Exercise | 3 Months |
| Hammer 2015(195) | Prospective Randomised controlled trial | 21 | 17 | 4 | 66.9 | 1-3 | dark chocolate | Medication | 7 Days |
| Prevost 2015(196) | Prospective Single-arm trial | 46 | 40 | 6 | 60.3 | 1-3 | Therapeutic education and personalised home-based exercise program | Exercise | 12 Months |
| Powell 2017(197) | Prospective Single-arm trial | 299 | 211 | 88 | 67.4 | 2-4 | Innova Vascular Self-Expanding Stent System | Surgical Intervention | 24 Months |
| Rundback 2017(198) | Prospective Single-arm trial | 75 | 46 | 29 | 64.2 | 2-4 | Visi-Pro Balloon-Expandable Peripheral Stent | Surgical Intervention | 9 Months |
| Derosa 2017(199) | Prospective Randomised controlled trial | 64 | 31 | 33 | 64 | 1 | mesogylcan 50mg BD | Medication | 6 Months |
| Girold 2017(200) | Prospective Randomised controlled trial | 42 | 35 | 7 | 57.2 | 2-3 | Nordic walking vs walking without poles. | Exercise | 30 Days |
| Liao 2019(201) | Prospective Randomised controlled trial | 74 | 40 | 34 | 67 | 2-5 | Orchid drug coated balloon | Surgical Intervention | 12 Months |
| Enzmann 2019(202) | Prospective Randomised controlled trial | 103 | 76 | 37 | 69 | 3-6 | Nitinol stent vs by-pass | Surgical Intervention | 12 Months |
| Rundback 2019(203) | Prospective Single-arm trial | 97 | 51 | 46 | 70 | 2-4 | Laser atherectomy | Surgical Intervention | 6 Months |
| Ahmad 2022(204) | Prospective Randomised controlled trial | 30 | 30 | NA | 65 | 1-3 | Laser acupuncture | Miscellaneous | 30 Days |
| Sachar 2021(205) | Prospective Randomised controlled trial | 376 | 240 | 136 | 69.85 | 2-4 | paclitaxel-coated balloon | Surgical Intervention | 12 Months |
| Iida 2021(206) | Prospective Randomised controlled trial | 86 | 64 | 22 | 74 | 2-4 | paclitaxel-coated balloon | Surgical Intervention | 24 Months |
| Monroe 2021(207) | Prospective Randomised controlled trial | 16 | 14 | 2 | 65.7 | 1-3 | Heat therapy vs supervised exercise therapy | Exercise | 3 Days |
| Shishehbor 2022(208) | Prospective Single-arm trial | 500 | 284 | 216 | 70.1 | 1-6 | Atherectomy device | Surgical Intervention | 30 Days |
| Bertges 2021(209) | Prospective Randomised controlled trial | 252 | 171 | 71 | 68 | 2-6 | Negative pressure wound therapy | Surgical Intervention | 30 Days |
| Han 2023(210) | Prospective Randomised controlled trial | 270 | 233 | 37 | 71 | 2-6 | sarpogrelate and aspirin compared to clopidogrel and aspirin | Medication | 6 Months |
| Ni 2022(211) | Prospective Randomised controlled trial | 192 | 138 | 54 | 69 | 3-5 | paclitaxel-coated balloon | Surgical Intervention | 12 Months |
| Therasse 2022(212) | Prospective Single-arm trial | 52 | 39 | 13 | 68 | 2-5 | SoundBite Crossing System‒Peripheral | Surgical Intervention | 30 Days |
| Hiatt 2021(213) | Retrospective Cohort study | 13885 | 9997 | 3888 | 67 | 0-6 | Risk stratification | Miscellaneous | 30 Months |
| Goueffic 2020(214) | Prospective Randomised controlled trial | 181 | 124 | 57 | 69.5 | 2-5 | paclitaxel eluting stent | Surgical Intervention | 24 Months |
| Saaya 2022(215) | Prospective Randomised controlled trial | 238 | 179 | 59 | 63 | 3-6 | Stenting versus endarterectomy | Surgical Intervention | 48 months |
| Hoel 2021(216) | Prospective Randomised controlled trial | 72 | 51 | 21 | 73 | 1-3 | Intermittent negative pressure | Miscellaneous | 3 Months |
| Guo 2021(217) | Retrospective Cohort study | 102 | 61 | 42 | 68 | 2-5 | DCB versus Stenting | Surgical Intervention | 12 Months |
| Ren 2021(218) | Prospective Randomised controlled trial | 144 | 112 | 32 | 66 | 2-5 | Paclitaxel coated balloon | Surgical Intervention | 24 Months |
| Rymer 2020(219) | Retrospective Cohort study | 13801 | NA | NA | NA | 0-6 | Risk assossiation | Miscellaneous | 24 Months |
| Rastan 2021(220) | Prospective Randomised controlled trial | 80 | 61 | 19 | 72 | 0-6 | Atherectomy | Surgical Intervention | 12 Months |
| Shammas 2022(221) | Retrospective Cohort study | 56 | 37 | 19 | 71 | 1-6 | Laser atherectomy | Surgical Intervention | 6 Months |
| DeBeaufort 2021(222) | Prospective Randomised controlled trial | 37 | 28 | 9 | 62 | 2-6 | Image fusion | Miscellaneous | NA |
| Yoshioka 2021(223) | Retrospective Cohort study | 156 | 108 | 48 | 75 | 2-6 | Bleeding risk | Miscellaneous | 12 Months |
| Sami 2021(224) | Prospective Cohort study | 20 | 9 | 11 | 61 | 2-6 | Cardiac rehablitation | Miscellaneous | 3 Months |
| Siercke 2021(225) | Prospective Randomised controlled trial | 118 | 69 | 49 | 70 | 2-3 | Cardiac rehablitation | Miscellaneous | 3 Months |
| Weissler 2021(226) | Prospective Cohort study | 1189 | 770 | 419 | 70 | 2-6 | Endovascular treatment | Surgical Intervention | 12 Months |
| Miura 2022(227) | Prospective Cohort study | 82 | 52 | 30 | 74 | 2-4 | Cilostazol treatment | Medication | 12 Months |
| Lindholt 2021(228) | Retrospective Cohort study | 50168 | 50168 | NA | 70 | 0-6 | Screening | Miscellaneous | 60 Months |
| Spoorendonk 2021(229) | Prospective Cohort study | NA | NA | NA | 68 | 2-6 | Cost-effectiveness | Miscellaneous | NA |
| Yoshioka 2022(230) | Retrospective Cohort study | 151 | 98 | 53 | 75 | 2-6 | Paclitaxel coated stent/balloon | Surgical Intervention | 12 Months |
| Deloose 2020(231) | Prospective Single-arm trial | 120 | 79 | 41 | 71 | 2-4 | Paclitaxel coated balloon and Amorphus silicon carbide coated stent | Surgical Intervention | 24 Months |
| Sun 2021(232) | Prospective Randomised controlled trial | 95 | 75 | 20 | 66 | 2-5 | Paclitaxel coatted balloon | Surgical Intervention | 24 Months |
| VanReijen 2022(233) | Retrospective Cohort study | 240 | 146 | 94 | 62 | 2-3 | Cost-effectiveness | Miscellaneous | NA |
| Djerf 2021(234) | Prospective Randomised controlled trial | 84 | 43 | 41 | 71 | 2-3 | Cost-effectiveness | Miscellaneous | 24 Months |
| Iwai 2023(235) | Prospective Cohort study | 1068 | 766 | 302 | 72 | 2-6 | Endovascular treatment | Surgical Intervention | 24 Months |
| Babaev 2022(236) | Prospective Randomised controlled trial | 60 | 36 | 24 | 71 | 3 | Directional atherectomy | Surgical Intervention | 12 Months |
| Goueffic 2021(237) | Prospective Randomised controlled trial | 153 | 137 | 16 | 63 | 1-3 | Cost-effectiveness | Miscellaneous | 30 Days |
| Starodubtsev 2022(238) | Prospective Randomised controlled trial | 202 | 187 | 15 | 62 | 2-5 | Hybrid vs open surgery | Surgical Intervention | 36 Months |
| Koelemay 2022(239) | Prospective Randomised controlled trial | 240 | 146 | 94 | 62 | 2-3 | Supervised exercise vs endovascualar therapy | Surgical Intervention | 66 Months |
| Sandberg 2022(240) | Prospective Randomised controlled trial | 166 | 98 | 68 | 72.1 | 1-3 | supervised exercise, home-based exercise, or walk advice | Exercise | 12 Months |
| Bearne 2022(241) | Prospective Randomised controlled trial | 190 | 133 | 57 | 68 | 1-3 | Home-Based, Walking Exercise Behavior Change Intervention | Exercise | 3 Months |
| Domingues 2021(242) | Prospective Randomised controlled trial | 29 | 15 | 14 | 64.4 | 1-3 | Creatinine supplementation | Medication | 2 Months |
| McDermott 2021(243) | Prospective Randomised controlled trial | 305 | 159 | 146 | 69 | 0-3 | low intersity vs high intensity walking | Exercise | 12 Months |
| Bohme 2022(244) | Retrospective Cohort study | 576 | 438 | 138 | 75.5 | 1-5 | Paclitaxel coated balloon | Surgical Intervention | 46 Months |
| Singh 2021(245) | Prospective Randomised controlled trial | 79 | NA | NA | NA | 2-3 | Pentoxyfilline | Medication | 3 Months |
| McDermott 2022(246) | Prospective Randomised controlled trial | 114 | 68 | 46 | 67 | 0-3 | Telmisartan | Medication | 6 Months |
| Haile 2022(247) | Prospective Randomised controlled trial | 204 | 108 | 94 | 72 | 2-3 | person-centred, nurse-led follow-up programme on adherence to prescribed medication | Miscellaneous | 12 Months |
| Dias-Santos 2021(248) | Prospective Randomised controlled trial | 12 | 4 | 8 | 66 | 1-3 | exercise mode | Exercise | NA |
| Pasqualini 2021(249) | Prospective Randomised controlled trial | 65 | 45 | 15 | 71 | 0-3 | Home-based exercise | Exercise | 3 Months |
| Slysz 2021(250) | Prospective Randomised controlled trial | 176 | 104 | 72 | 67 | 1-3 | Supervised exercise therapy | Exercise | 6 Months |
| Suchkov 2022(251) | Prospective Randomised controlled trial | 362 | 312 | 50 | 63 | 2-3 | Actovegin | Medication | 6 Months |
| Kalantzi 2020(252) | Prospective Randomised controlled trial | 794 | 508 | 288 | 68 | 1-4 | Cilostazol treatment | Medication | 27 Months |
| Teichgraber 2020(253) | Prospective Randomised controlled trial | 171 | NA | NA | NA | 2-5 | Paclitaxel drug coated balloon | Surgical Intervention | 12 Months |
| Bonaca 2022(254) | Retrospective Cohort study | 2185 | NA | NA | NA | 2-6 | Rivaroxaban with Aspirin | Medication | 36 Months |
| Kook 2022(255) | Prospective Randomised controlled trial | 170 | 145 | 25 | 71 | 1-4 | SID142 | Medication | 3 Months |
| Gilchrist 2021(256) | Retrospective Cohort study | 5845 | 4185 | 1660 | 65 | 1-6 | Vorapaxr and statins | Medication | 36 Months |
| Goueffic 2022(257) | Prospective Randomised controlled trial | 775 | 543 | 232 | 70 | 2-4 | Paclitaxel eluting stent | Surgical Intervention | 12 Months |
| Caradu 2022(258) | Retrospective Cohort study | 255 | 185 | 70 | 77 | 2-6 | EndoNaut fusion imaging | Miscellaneous | NA |
| Baig 2022(259) | Retrospective Cohort study | 50 | 37 | 13 | 75 | 3-6 | Endovascular lithotripsy | Surgical Intervention | 18 Months |
| Fakhry 2021(260) | Retrospective Cohort study | 212 | 132 | 80 | 65 | 1-3 | supervised exercise and endovascular revascularisation | Exercise | 12 Months |
| Peri-Okonny 2021(261) | Retrospective Cohort study | 483 | 265 | 218 | 69 | 1-3 | NA | NA | 6 Months |
| Bohme 2020(262) | Retrospective Cohort study | 1579 | 1023 | 556 | 71 | 1-4 | Drug coated balloon | Surgical Intervention | 60 Months |
| Monroe 2020(263) | Prospective Randomised controlled trial | 30 | 26 | 4 | 69 | 1-3 | Heat therapy | Miscellaneous | 2 Months |
| Bohme 2021(264) | Prospective Single-arm trial | 71 | 50 | 21 | 66.7 | 2-4 | Heparin-Bonded Stent-Graft | Surgical Intervention | 36 Months |
| Kim 2023(265) | Retrospective Cohort study | 4856 | 2906 | 1950 | 67 | 2-4 | Paclitaxel coated devices | Surgical Intervention | 12 Months |
| Parodi 2020(266) | Prospective Cohort study | 132 | 94 | 38 | 72.5 | 3-4 | hydration and albumin supplementation | Medication | 6 Months |
| Busch 2021(267) | Prospective Cohort study | 102 | 73 | 29 | 73 | 2-3 | Platelet assay | Miscellaneous | 6 Months |
| Johnson 2022(268) | Retrospective Cohort study | 287 | 193 | 94 | 69.8 | 2-6 | Blood transfusion | Miscellaneous | 20 Months |
| Englund 2022(269) | Prospective Randomised controlled trial | 148 | 76 | 72 | 67 | 1-3 | Supervised exercise therapy | Exercise | 3 Months |
| McCallum 2021(270) | Prospective Cohort study | 30 | 21 | 9 | 60.5 | 1-3 | supported self-management through low-intensity psychological intervention | Miscellaneous | 6 Months |
| Fukaya 2021(271) | Prospective Randomised controlled trial | 41 | 24 | 17 | 65 | 1-3 | Incentivised exercise monitoring | Exercise | 3 Months |
| Mathlouthi 2021(272) | Retrospective Cohort study | 296 | 187 | 113 | 66 | 2-6 | Paclitaxel eluting stent | Surgical Intervention | 23 Months |
| Kato 2022(273) | Prospective Randomised controlled trial | 37 | 27 | 10 | 73 | 2-4 | Intensive exercise | Exercise | 12 Months |
| Lawall 2021(274) | Prospective Randomised controlled trial | 73 | 55 | 18 | 65 | 2-3 | Sublingual nitroglycerin | Medication | 6 Months |
| Tepe 2021(275) | Prospective Randomised controlled trial | 256 | 226 | 30 | 72 | 2-4 | Intravascular lithotripsy | Surgical Intervention | 30 Days |
| Shammas 2022(276) | Prospective Randomised controlled trial | 47 | 31 | 16 | 71 | 2-5 | Jet stream atherectomy | Surgical Intervention | 12 Months |
| Kokkinidis 2020(277) | Retrospective Cohort study | 117 | 90 | 27 | 70 | 2-6 | Laser atherectomy | Surgical Intervention | 24 Months |
| Schahab 2021(278) | Retrospective Cohort study | 287 | 23 | 54 | 71 | 3-5 | Paclitaxel coated balloon | Surgical Intervention | 24 Months |
| Teichgraber 2022(279) | Prospective Randomised controlled trial | 171 | 130 | 41 | 68 | 2-5 | Paclitaxel coated balloon | Surgical Intervention | 60 Months |
| Klaphake 2022(280) | Prospective Randomised controlled trial | 212 | 132 | 82 | 65 | 1-3 | EVT with SET vs SET alone | Surgical Intervention | 63 Months |
| Gunnarsson 2023(281) | Prospective Randomised controlled trial | 94 | 50 | 40 | 71 | 2-3 | SFA Stenting | Surgical Intervention | 60 Months |
| Kluckner 2022(282) | Prospective Cohort study | 103 | 69 | 44 | 69 | 3-6 | Stenting versus by-pass | Surgical Intervention | 48 months |
| Ohki 2021(283) | Prospective Single-arm trial | 103 | 85 | 18 | 74 | 2-5 | Hepain-bonded stent graft | Surgical Intervention | 60 Months |
| Eikelboom 2022(284) | Prospective Cohort study | 12964 | 10085 | 2879 | 67 | 1-6 | Rivaroxaban with Aspirin | Medication | 18 Months |
| Krantz 2021(285) | Prospective Randomised controlled trial | 1330 | 806 | 524 | 78 | 2-6 | Rivaroxaban with Aspirin | Medication | 36 Months |
| Hoel 2021(286) | Prospective Cohort study | 10 | 7 | 3 | 71 | 1-3 | Intermittent negative pressure | Miscellaneous | 6 Months |
| Nowakowski 2021(287) | Prospective Randomised controlled trial | 66 | 53 | 13 | 65 | 2-5 | paclitaxel coated balloon | Surgical Intervention | 36 Months |
| Shibutani 2020(288) | Prospective Single-arm trial | 113 | 74 | 39 | 73 | 2-6 | Heparin bonded ePTFE graft | Surgical Intervention | 16 Months |
| Pekas 2023(289) | Prospective Cohort study | 10 | 4 | 6 | 68 | 1-3 | dietary nitrate | Miscellaneous | 3 Months |
| Eikelboom 2021(290) | Prospective Randomised control trial | 18274 | 14230 | 4048 | 68 | 1-6 | Rivaroxaban with Aspirin | Medication | 30 Months |
| Tan 2021(291) | Retrospective Cohort study | 481 | 361 | 120 | 73 | 2-5 | Pacilatex coated stent | Surgical Intervention | 24 Months |
| Nordanstig 2020(292) | Prospective Randomised controllled trial | 2289 | 1254 | 1035 | 75 | 2-6 | Drug coated devcies | Surgical Intervention | 48 months |
| Coca-Martinez 2021(293) | Prospective Cohort study | 5 | NA | NA | 76 | 2-4 | Pre-hablitation | Exercise | 3 Months |
| Mietz 2020(294) | Prospective Randomised controlled trial | 171 | 130 | 41 | 68 | 2-5 | Paclitaxel coated balloon | Surgical Intervention | 12 Months |
| Tsai 2022(295) | Prospective Randomised controlled trial | 66 | 63 | 3 | 68 | 1-3 | Vorapaxar | Medication | 3 Months |
| Soga 2022(296) | Prospective Randomised controlled trial | 102 | 78 | 24 | 74 | 2-4 | Paclitaxel coated balloon | Surgical Intervention | 12 Months |
| Brodmann 2020(297) | Prospective Single-arm trial | 201 | 118 | 83 | 65 | 2-3 | Tack endovascular system | Surgical Intervention | 12 Months |
| Zeller 2022(298) | Prospective Randomised controlled trial | 66 | 51 | 14 | 75 | 3-5 | orbital atherectomy system | Surgical Intervention | 12 Months |
| Liao 2022(299) | Prospective Randomised controlled trial | 60 | 38 | 22 | 69 | 2-5 | paclitaxel coated balloon | Surgical Intervention | 12 Months |
| Kozuki 2020(300) | Retrospective Cohort study | 44 | 32 | 12 | 74 | 2-6 | Risk stratification | Miscellaneous | 12 Months |
| Kim 2021(301) | Retrospective Cohort study | 366 | 237 | 129 | 70 | 2-6 | Paclitaxel coated devices | Surgical Intervention | 36 Months |
| Gahide 2021(302) | Retrospective Cohort study | 700 | 508 | 192 | 68 | 2-3 | Paclitaxel coated devices | Surgical Intervention | 60 Months |
| Tepe 2021(303) | Prospective Single-arm trial | 877 | 561 | 316 | 64 | 0-6 | Paclitaxel coated balloons | Surgical Intervention | 24 Months |
| Ribeiro 2022(304) | Retrospective Cohort study | 351 | 268 | 83 | 70 | 1-6 | Paclitaxel coated devices | Surgical Intervention | 55 months |
| Zielinski 2021(305) | Retrospective Cohort study | 153 | 90 | 63 | 73 | 3-6 | Cost | Miscellaneous | 24 Months |
| Fox 2022(306) | Prospective Cohort study | 5532 | 4119 | 1413 | 68 | 1-6 | Rivaroxaban with Aspirin | Medication | 12 Months |
| Bohme 2021(307) | Prospective Randomised controlled study | 61 | 43 | 18 | 68 | 1-5 | Laser atherectomy | Surgical Intervention | 24 Months |
| Pan 2022(308) | Retrospective Cohort study | 327 | 271 | 56 | 72 | 2-6 | Risk prediction | Miscellaneous | 24 Months |
| Moll 2022(309) | Prospective Cohort study | 150 | 95 | 55 | 73 | 3-6 | Risk stratification | Miscellaneous | NA |
| Berkowitz 2021(310) | Prospective Randomised controlled trial | 6564 | 4860 | 1704 | 67 | 2-6 | Rivaroxaban with Aspirin | Medication | 30 Months |
| Brouillet 2018(311) | Retrospective Cohort study | 203 | 145 | 58 | 70 | 2-6 | Stenting | Surgical Intervention | 12 Months |
| Sharma 2021(312) | Prospective Randomised controlled trial | 81 | 77 | 4 | 46 | 3-6 | Angiogenesis Induced by Intraarterial Autologous Bone Marrow–Derived Stem Cells | Surgical Intervention | 6 Months |
| Shishehbor 2022(313) | Prospective Randomised controlled trial | 313 | 180 | 133 | 70 | 2-4 | Paclitaxel coated balloon | Surgical Intervention | 12 Months |
| Jiang 2021(314) | Prospective Randomised controlled trial | 32 | 20 | 12 | 65 | 1-3 | Sonodynamic therapy | Miscellaneous | 30 Days |
| Ye 2020(315) | Prospective Randomised controlled trial | 200 | 145 | 55 | 68 | 2-5 | paclitaxel coated balloon | Surgical Intervention | 24 Months |
| Kaplovitch 2020(316) | Prospective Randomised controlled trial | 4129 | 2932 | 1197 | 67 | 1-6 | Rivaroxaban with Aspirin | Medication | 30 Months |
| Guzik 2021(317) | Prospective Randomised controlled trial | 27207 | 21244 | 5963 | 68 | 1-6 | Rivaroxaban with Aspirin | Medication | 30 Months |
| Imran 2017(318) | Retrospective Cohort study | 70 | 46 | 24 | 71 | 2-6 | Paclitaxel coated balloon | Surgical Intervention | 12 Months |
| Hess 2021(319) | Retrospective Cohort study | 4316 | 3079 | 1237 | 68 | 2-6 | Paclitaxel coated devices | Surgical Intervention | 30 Months |
| Ali 2022(320) | Prospective Randomised controlled trial | 60 | 37 | 23 | 51 | 2 | shock wave therapy | Miscellaneous | 2 Months |
| Bosiers 2020(321) | Prospective Randomised controlled trial | 83 | 61 | 22 | 68 | 2-5 | stents grafts | Surgical Intervention | 24 Months |
| Paldan 2021(322) | Prospective Randomised controlled trial | 39 | 21 | 18 | 65 | 1-3 | SET using mobile app | Exercise | 3 Months |
| Slysz 2021(323) | Prospective Cohort study | 277 | 149 | 128 | 69 | 1-3 | Exercise | Exercise | 12 Months |
| Nasr 2020(324) | Retrosepctive Cohort study | 54 | 46 | 8 | 68 | 3-6 | CFA Stenting | Surgical Intervention | 24 Months |
| Nastasi 2020(325) | Prospective Cohort study | 783 | 561 | 22 | 67 | 1-6 | Intensive lipid lowering | Medication | 26 Months |
| Pettersen 2022(326) | Retrospective Cohort study | 62 | 42 | 20 | 73 | 1-3 | Interittent negative pressure | Miscellaneous | 6 Months |
| Kapusta 2022(327) | Prospective Randomised controlled trial | 100 | 59 | 41 | 60 | 0-3 | Hydrotherapy | Miscellaneous | NA |
| Allan 2022(328) | Prospective Randomised controlled trial | 150 | 96 | 54 | 73 | 3-6 | IVUS during angioplasty | Surgical Intervention | 12 Months |
| Schroe 2022(329) | Retrospective Cohort study | 129 | 78 | 41 | 71 | 2-4 | Paclitaxel coated balloon | Surgical Intervention | 12 Months |
| Tan 2022(330) | Retrospective Cohort study | 250 | 149 | 101 | 75 | 2-6 | self-expanding nitinol stent | Surgical Intervention | 36 Months |
| Giannopoulos 2021(331) | Prospective Cohort study | 1189 | NA | NA | 70 | 2-6 | EVT | Surgical Intervention | 36 Months |
| Soga 2020(332) | Prospective Randmised controlled trial | 100 | 76 | 24 | 74 | 2-4 | Paclitaxel coated balloon | Surgical Intervention | 36 Months |
| Salisbury 2022(333) | Prospective Randmised controlled trial | 19 | 13 | 6 | NA | 2-3 | TBR versus SET | Exercise | 3 Months |
| Szarek 2022(334) | Prospective Cohort study | 13885 | 9997 | 3888 | 66 | 0-6 | Ticagrelor vs clopidogrel | Medication | 30 Months |
| Bauersachs 2021(335) | Prospective Randomised controlled trial | 6564 | 4860 | 1704 | 67 | 2-6 | Rivaroxaban with Aspirin | Medication | 30 Months |
| Saratzis 2021(336) | Retrospective Cohort study | 116 | 88 | 28 | 65 | 1-6 | CERAB | Surgical Intervention | 36 Months |
| Sullivan 2021(337) | Prospective Single-arm trial | 271 | 180 | 91 | 68 | 2-4 | BioMimics 3D Vascular Stent System | Surgical Intervention | 24 Months |
| Nasr 2023(338) | Prospective Single-arm trial | 48 | 39 | 9 | 72 | 2-6 | Supera stent | Surgical Intervention | 24 Months |
| Muller-Hulsbeck 2021(339) | Prospective Randomised controlled trial | 465 | 308 | 157 | 68 | 2-4 | Eluvia stent vs Zilver PTX | Surgical Intervention | 24 Months |
| Shammas 2021(340) | Retrospective Cohort study | 89 | 51 | 48 | 65 | 2-6 | CFA EVT | Surgical Intervention | 24 Months |
| Fukuda 2021(341) | Retrospective Cohort study | 404 | 303 | 101 | 72 | 2-6 | Ultrasound guided puncture in EVT | Surgical Intervention | NA |
| Landry 2021(342) | Prospective Cohort study | 10 | 5 | 5 | 70 | 1-3 | Ultrasound therapy | Miscellaneous | 2 Months |
| Hogan 2022(343) | Prospective Cohort study | 5614 | 4171 | 1443 | 67 | 2-6 | Lower limb revascularisation | Surgical Intervention | 36 Months |
| Manfredini 2024(344) | Prospective Randomised controlled trial | 68 | 50 | 18 | 73 | 2-3 | Home based exersice therapy | Exercise | 6 Months |
| Fang 2024(345) | Prospective Single-arm trial | 160 | 126 | 34 | 67.4 | 2-5 | Paclitaxel coated balloon | Surgical Intervention | 12 Months |
| Iida 2024(346) | Prospective Single-arm trial | 134 | 109 | 25 | 74 | 2-4 | Sirolimus-coated balloons | Surgical Intervention | 12 Months |
| Bertges 2024(347) | Prospective Single-arm trial | 300 | 174 | 126 | 72 | 2-4 | Paclitaxel coated balloon | Surgical Intervention | 36 Months |
| Noory 2024(348) | Prospective Single-arm trial | 955 | 594 | 391 | 69.7 | 2-5 | JetStream Atherectomy device | Surgical Intervention | 24 Months |
| Herrero-Alonso 2024(349) | Prospective Randomised controlled trial | 122 | 112 | 10 | 67.8 | 1-3 | Supervised Exersice | Exercise | 12 Months |
| Rrapo-Kaso 2023(350) | Prospective Randomised controlled trial | 35 | 15 | 20 | 64 | 2-4 | Alicorumab | Medication | 12 Months |
| Liu 2023(351) | Retrospective Cohort study | 1260 | 964 | 256 | 68 | 3-6 | Risk prediction | Miscellaneous | 12 Months |
| Mcbane 2024(352) | Retrospective Cohort study | 10437 | 6209 | 4228 | 66 | 1-6 | Risk prediction | Miscellaneous | 60 Months |
| Bentzen 2023(353) | Prospective Cohort study | 16 | 10 | 6 | 67 | 2-3 | Blood flow restricted walking | Exercise | 3 Months |
| Vrsalovic 2024(354) | Prospective Cohort study | 1898 | 1228 | 670 | 66.7 | 1-6 | Effect of hypertension | Miscellaneous | 72 Months |
| Bohme 2023(355) | Prospective Cohort study | 119 | 68 | 51 | 70 | 2-4 | Chocolate touch balloon | Surgical Intervention | 12 Months |
| Ulfsdottir 2023(356) | Prospective Cohort study | 149 | 88 | 61 | 72 | 1-3 | Supervised exercise | Exercise | 12 Months |
| Lyden 2024(357) | Prospective Single-arm trial | 202 | 149 | 53 | 69 | 3-5 | DETOUR Device | Surgical Intervention | 12 Months |
| Xiong 2024(358) | Prospective Cohort study | 816 | 567 | 249 | 75 | 1-6 | Risk prediction | Miscellaneous | 12 Months |
| Kodama 2024(359) | Retrosepctive Cohort study | 64 | 41 | 23 | 70 | 2-5 | Paclitaxel coated balloons | Surgical Intervention | 12 Months |
| Danisan 2023(360) | Prospective Randomised controlled trial | 85 | 61 | 24 | 63 | 3-6 | N-Acetylcysteine | Medication | 3 Days |
| Silva 2023(361) | Prospective Randomised controlled trial | 73 | 64 | 9 | 64 | 1-3 | Home based exersice therapy | Exercise | 6 Months |
| Garcia 2023(362) | Prospective Randomised controlled trial | 17 | 9 | 8 | 63 | 1-3 | Exercise vs exercise and resistance training | Exercise | 3 Months |
| Gardner 2023(363) | Prospective Randomised controlled trial | 72 | 34 | 38 | 65 | 1-3 | Home based exersice therapy | Exercise | 18 Months |
| Perks 2023(364) | Prospective Cohort study | 29 | 19 | 10 | 69 | 1-3 | Breaking up sitting time | Exercise | 2 Months |
| Arora 2024(365) | Prospective Randomised controlled trial | 133 | 83 | 50 | 60 | 1-3 | Structured exercise program | Exercise | 3 Months |
| Cai 2024(366) | Prospective Randomised controlled trial | 138 | 92 | 46 | 67 | 1-3 | shock wave therapy | Miscellaneous | 3 Months |
| Waddell 2024(367) | Prospective Randomised controlled trial | 30 | 26 | 4 | 68.2 | 1-3 | Home based exersice therapy | Exercise | 3 Months |
| Cerrud-Rodriguez 2024(368) | Prospective Cohort study | 581 | 382 | 199 | 66 | 1-3 | Early intervention for PAD | Surgical Intervention | 12 Months |
| Sandberg 2023(369) | Prospective Randomised controlled trial | 166 | 98 | 68 | 72 | 1-3 | Different types of exercise therapy |  | 12 Months |
| Shammas 2023(370) | Prospective Randomised controlled trial | 40 | NA | NA | NA | NA | JetStream Atherectomy device | Surgical Intervention | 24 Months |
| Bonaca 2024(371) | Prospective Randomised controlled trial | 1687 | 1232 | 455 | 68 | 1-6 | Ticagrelor | Medication | 40 Months |
| Caruso 2024(372) | Prospective Randomised controlled trial | 55 | 43 | 12 | 67.3 | 2-5 | Liraglutide | Medication | 6 Months |
| Katsogridakis 2024(373) | Retrosepctive Cohort study | 2041 | 1379 | 662 | 71.5 | 2-6 | Effect of AKI on outcome | Miscellaneous | 3 Months |
| Nakama 2023(374) | Prospective Single-arm trial | 581 | 381 | 200 | 75 | 2-5 | High dose vs low dose paclitaxel devices | Surgical Intervention | 12 Months |
| Parkington 2023(375) | Prospective Randomised controlled trial | 30 | 24 | 6 | 68 | 1-2 | Low-intensity resistance exercise | Exercise | 3 Months |
| Wargny 2024(376) | Retrosepctive Cohort study | 20083 | 13474 | 6609 | 72 | 2-6 | Drug coated devices | Surgical Intervention | 24 Months |
| Coca-Martinez 2023(377) | Prospective Randomised controlled trial | 24 | 10 | 14 | 70 | 1-3 | Prehablitation | Exercise | 12 Months |
| Fransson 2024(378) | Retrospective Cohort study | 1948 | 1062 | 886 | 71 | 2-6 | Drug coated devices | Surgical Intervention | 36 Months |
| Burgess 2023(379) | Prospective Randomised controlled trial | 190 | 140 | 50 | 67 | 1-3 | Neuromuscular Electrical stimulation | Miscellaneous | 3 Months |
| McDermott 2024(380) | Prospective Randomised controlled trial | 90 | 48 | 42 | 71 | 0-3 | Nicotinamide Riboside | Medication | 6 Months |
| Aragao 2023(381) | Prospective Cohort study | 127 | 69 | 58 | 69 | 1-6 | NA | NA | 3 Months |
| Ducasse 2024(382) | Prospective Randomised controlled trial | 134 | 107 | 27 | 69 | 2-5 | Excimer laser atherectomy | Surgical Intervention | 18 Months |
| Haile 2023(383) | Prospective Randomised controlled trial | 183 | 98 | 85 | 72 | 2-3 | Person Centred Nurse Led Follow Up Programme | Miscellaneous | 12 Months |
| Cleary 2024(384) | Prospective Cohort study | 150 | 99 | 51 | 70.5 | 1-3 | Barriers for SET | Miscellaneous | NA |
| Rahman 2024(385) | Prospective Cohort study | 140 | 104 | 36 | 63 | 0-3 | Qait characteristics in Claudicants | Miscellaneous | NA |
| Donas 2023(386) | Prospective Single-arm trial | 62 | 35 | 27 | 77 | 3-5 | Rotational atherectomy | Surgical Intervention | 24 Hours |
| Gray 2024(387) | Prospective Randomised controlled trial | 465 | 308 | 157 | 68 | 2-4 | Zilver PTX vs Eluvia | Surgical Intervention | 60 Months |
| Bohr 2024(388) | Retrosepctive Cohort study | 163 | 85 | 78 | 67 | 2-5 | Endovascular internvetion | Surgical Intervention | 12 Months |
| Scierka 2024(389) | Prospective Cohort study | 581 | 382 | 199 | 66 | 1-3 | Early intervention for PAD | Surgical Intervention | 12 Months |
| Yuan 2024(390) | Prospective Randomised controlled trial | 6564 | 4860 | 1704 | 67 | 2-6 | Rivaroxaban with Aspirin | Medication | 30 Months |
| Wittig 2024(391) | Prospective Randomised controlled trial | 120 | 94 | 26 | 66 | 2-4 | Femoropopliteal stent | Surgical Intervention | 12 Months |
| Amanvermez Senarslan 2023(392) | Prospective Cohort study | 18 | 14 | 4 | 60 | 3-6 | CERAB | Surgical Intervention | 54 Months |
| Govsyeyev 2023(393) | Prospective Cohort study | 1419 | 1140 | 279 | 66 | 1-5 | Surgical bypass | Surgical Intervention | 36 Months |
| Schofthaler 2024(394) | Prospective Cohort study | 122 | 62 | 60 | 74 | 3-6 | Phoenix atherectomy device | Surgical Intervention | 36 months |
| Nagpal 2024(395) | Prospective Cohort study | 1262 | 885 | 377 | 72 | 1-6 | Intravascular lithotripsy | Surgical Intervention | 36 Months |
| Franzese 2023(396) | Prospective Single-arm trial | 80 | 66 | 14 | 70 | 3-5 | Sirolimus-coated balloons | Surgical Intervention | 12 Months |
| Kronlage 2023(397) | Retrospective Cohort study | 264 | 171 | 93 | 75.6 | 3-6 | Rotational atherectomy | Surgical Intervention | 19 Months |
| Krishnan 2024(398) | Prospective Randomised controlled trial | 300 | 176 | 124 | 69 | 2-4 | Stellarex drug-coated balloon | Surgical Intervention | 60 Months |
| Rezvani 2024(399) | Prospective Randomised controlled trial | 2275 | 1570 | 705 | 66.5 | 1-2 | Telephone health coaching and exercise | Exercise | 24 Months |
| Cawich 2024(400) | Prospective Randomised controlled trial | 53 | 35 | 18 | 72.7 | 3-5 | Temsirolimus | Surgical Intervention | 6 Months |
| Clavijo 2023(401) | Prospective Randomised controlled trial | 70 | 43 | 27 | 68 | 1-4 | Evolucumab | Medication | 6 Months |
| Heider 2024(402) | Prospective Randomised controlled trial | 2275 | 1570 | 705 | 66.5 | 1-2 | Telephone health coaching and exercise | Exercise | 24 Months |
| Sandberg 2023(403) | Prospective Randomised controlled trial | 166 | 98 | 68 | 72.1 | 1-3 | Nordic pole walking | Exercise | 12 Months |
| Hagen 2024(404) | Prospective Cohort study | 11 | 5 | 6 | 62 | 2-3 | Nordic pole waking | Exercise | 3 Months |
| Trorsello 2024(405) | Prospective Single-arm trial | 130 | 82 | 48 | 71 | 2-6 | Eluvia stent | Surgical Intervention | 60 Months |
| Ozpak 2023(406) | Retrospective Cohort study | 118 | 68 | 50 | 66.3 | 2-6 | Paclitaxel-Coated Balloon | Surgical Intervention | 12 Months |
| Enzmann 2023(407) | Prospective Randomised controlled trial | 209 | 147 | 62 | 69 | 3-6 | Vein bypass vs Nitinol stent | Surgical Intervention | 48 Months |
| Bosiers 2023(408) | Prospective Randomised controlled trial | 220 | 159 | 61 | 69 | 2-5 | ZILVER PTX stent | Surgical Intervention | 60 Months |

# **References**

1. Yonemitsu Y, Matsumoto T, Maehara Y. R&D of DVC1-0101 as a new class of RNA gene medicine to treat peripheral arterial disease. Journal of Pharmacological Sciences. 2015;128(3 SUPPL. 1):S55.

2. Gupta P, Momsen AMH, Norager CB, Lindholt JS, Madsen MR, Jensen MB. The Effect of Caffeine in Patients with Intermittent Claudication Was Independent of the Degree of Peripheral Ischemia - A Secondary Analysis of a Randomized Clinical Trial. Journal of Caffeine Research. 2015;5(2):89-93.

3. Leicht A, Crowther R, Golledge J. Influence of regular exercise on body fat and eating patterns of patients with intermittent claudication. International Journal of Molecular Sciences. 2015;16(5):11339-54.

4. Markovic MD, Markovic DM, Dragas MV, Koncar IB, Banzic IL, Ille ME, et al. The role of kinesitherapy and electrotherapeutic procedures in non-operative management of patients with intermittent claudications. Vascular. 2015;24(3):246-53.

5. Janas AJ, Milewski KP, Buszman PP, Trendel W, Kolarczyk-Haczyk A, Hochul M, et al. Long term outcomes in diabetic patients treated with atherectomy for peripheral artery disease. Cardiology journal. 2020;27(5):600-7.

6. Cochrane DJ, Cochrane F, Roake JA. An exploratory study of vibration therapy on muscle function in patients with peripheral artery disease. Journal of vascular surgery. 2020;71(4):1340-5.

7. Park S-Y, Pekas EJ, Headid RJ, 3rd, Son W-M, Wooden TK, Song J, et al. Acute mitochondrial antioxidant intake improves endothelial function, antioxidant enzyme activity, and exercise tolerance in patients with peripheral artery disease. American journal of physiology Heart and circulatory physiology. 2020;319(2):H456-H67.

8. Djerf H, Millinger J, Falkenberg M, Jivegard L, Svensson M, Nordanstig J. Absence of Long-Term Benefit of Revascularization in Patients With Intermittent Claudication: Five-Year Results From the IRONIC Randomized Controlled Trial. Circ Cardiovasc Interv. 2020;13(1):e008450.

9. Angle JF, Gasparetto A, Yokoi H, Jaff MR, Popma JJ, Piegari GN, Jr., et al. Three-Year Efficacy and Safety of the Misago Peripheral Stent for Superficial Femoral Artery Disease: Final Results from the OSPREY Trial. Journal of vascular and interventional radiology : JVIR. 2020;31(6):978-85.

10. Janas A, Milewski K, Buszman P, Kolarczyk-Haczyk A, Trendel W, Pruski M, et al. Comparison of long-term outcomes after directional versus rotational atherectomy in peripheral artery disease. Postepy w kardiologii interwencyjnej = Advances in interventional cardiology. 2020;16(1):76-81.

11. Hageman D, Fokkenrood HJP, van Deursen BAC, Gommans LNM, Cancrinus E, Scheltinga MRM, et al. Randomized controlled trial of vacuum therapy for intermittent claudication. Journal of vascular surgery. 2020;71(5):1692-701.e1.

12. McDermott MM, Criqui MH, Domanchuk K, Ferrucci L, Guralnik JM, Kibbe MR, et al. Cocoa to Improve Walking Performance in Older People With Peripheral Artery Disease: The COCOA-PAD Pilot Randomized Clinical Trial. Circulation research. 2020;126(5):589-99.

13. Bjorkman P, Auvinen T, Hakovirta H, Romsi P, Turtiainen J, Manninen H, et al. Drug-Eluting Stent Shows Similar Patency Results as Prosthetic Bypass in Patients with Femoropopliteal Occlusion in a Randomized Trial. Annals of vascular surgery. 2018;53(avs, 8703941):165-70.

14. Horie T, Yamazaki S, Hanada S, Kobayashi S, Tsukamoto T, Haruna T, et al. Outcome From a Randomized Controlled Clinical Trial - Improvement of Peripheral Arterial Disease by Granulocyte Colony-Stimulating Factor-Mobilized Autologous Peripheral-Blood-Mononuclear Cell Transplantation (IMPACT). Circulation journal : official journal of the Japanese Circulation Society. 2018;82(8):2165-74.

15. Micari A, Brodmann M, Keirse K, Peeters P, Tepe G, Frost M, et al. Drug-Coated Balloon Treatment of Femoropopliteal Lesions for Patients With Intermittent Claudication and Ischemic Rest Pain: 2-Year Results From the IN.PACT Global Study. JACC Cardiovascular interventions. 2018;11(10):945-53.

16. Baumhakel M, Chkhetia S, Kindermann M. Treatment of femoro-popliteal lesions with scoring and drug-coated balloon angioplasty: 12-month results of the DCB-Trak registry. Diagnostic and interventional radiology (Ankara, Turkey). 2018;24(3):153-7.

17. Razavi MK, Donohoe D, D'Agostino RB, Jr., Jaff MR, Adams G, Investigators D. Adventitial Drug Delivery of Dexamethasone to Improve Primary Patency in the Treatment of Superficial Femoral and Popliteal Artery Disease: 12-Month Results From the DANCE Clinical Trial. JACC Cardiovascular interventions. 2018;11(10):921-31.

18. Steiner S, Willfort-Ehringer A, Sievert H, Geist V, Lichtenberg M, Del Giudice C, et al. 12-Month Results From the First-in-Human Randomized Study of the Ranger Paclitaxel-Coated Balloon for Femoropopliteal Treatment. JACC Cardiovascular interventions. 2018;11(10):934-41.

19. Green JL, Harwood AE, Smith GE, Das T, Raza A, Cayton T, et al. Extracorporeal shockwave therapy for intermittent claudication: Medium-term outcomes from a double-blind randomised placebo-controlled pilot trial. Vascular. 2018;26(5):531-9.

20. McDermott MM, Spring B, Berger JS, Treat-Jacobson D, Conte MS, Creager MA, et al. Effect of a Home-Based Exercise Intervention of Wearable Technology and Telephone Coaching on Walking Performance in Peripheral Artery Disease: The HONOR Randomized Clinical Trial. JAMA. 2018;319(16):1665-76.

21. Soga Y, Hamasaki T, Edahiro R, Iida O, Inoue N, Suzuki K, et al. Sustained Effectiveness of Cilostazol After Endovascular Treatment of Femoropopliteal Lesions: Midterm Follow-up From the Sufficient Treatment of Peripheral Intervention by Cilostazol (STOP-IC) Study. Journal of endovascular therapy : an official journal of the International Society of Endovascular Specialists. 2018;25(3):306-12.

22. Albrecht T, Waliszewski M, Roca C, Redlich U, Tautenhahn J, Pech M, et al. Two-Year Clinical Outcomes of the CONSEQUENT Trial: Can Femoropopliteal Lesions be Treated with Sustainable Clinical Results that are Economically Sound? Cardiovascular and interventional radiology. 2018;41(7):1008-14.

23. Moll F, Baumgartner I, Jaff M, Nwachuku C, Tangelder M, Ansel G, et al. Edoxaban Plus Aspirin vs Dual Antiplatelet Therapy in Endovascular Treatment of Patients With Peripheral Artery Disease: Results of the ePAD Trial. Journal of endovascular therapy : an official journal of the International Society of Endovascular Specialists. 2018;25(2):158-68.

24. Yang X, Leesar MA, Ahmed H, Lendel V, Rodriguez G, Mutlu D, et al. Impact of ticagrelor and aspirin versus clopidogrel and aspirin in symptomatic patients with peripheral arterial disease: Thrombus burden assessed by optical coherence tomography. Cardiovascular revascularization medicine : including molecular interventions. 2018;19(7 Pt A):778-84.

25. Lindgren HIV, Qvarfordt P, Bergman S, Gottsater A, Swedish Endovascular Claudication Stenting T. Primary Stenting of the Superficial Femoral Artery in Patients with Intermittent Claudication Has Durable Effects on Health-Related Quality of Life at 24 Months: Results of a Randomized Controlled Trial. Cardiovascular and interventional radiology. 2018;41(6):872-81.

26. Schneider PA, Laird JR, Tepe G, Brodmann M, Zeller T, Scheinert D, et al. Treatment Effect of Drug-Coated Balloons Is Durable to 3 Years in the Femoropopliteal Arteries: Long-Term Results of the IN.PACT SFA Randomized Trial. Circulation Cardiovascular interventions. 2018;11(1):e005891.

27. Davins Riu M, Borras Perez X, Artigas Raventos V, Palomera Fanegas E, Serra Prat M, Alos Villacrosa J. Use of Telehealth as a New Model for Following Intermittent Claudication and Promoting Patient Expertise. Telemedicine journal and e-health : the official journal of the American Telemedicine Association. 2018;24(10):773-81.

28. Laird JR, Zeller T, Loewe C, Chamberlin J, Begg R, Schneider PA, et al. Novel Nitinol Stent for Lesions up to 24 cm in the Superficial Femoral and Proximal Popliteal Arteries: 24-Month Results From the TIGRIS Randomized Trial. Journal of endovascular therapy : an official journal of the International Society of Endovascular Specialists. 2018;25(1):68-78.

29. Iida O, Soga Y, Urasawa K, Saito S, Jaff MR, Wang H, et al. Drug-Coated Balloon vs Standard Percutaneous Transluminal Angioplasty for the Treatment of Atherosclerotic Lesions in the Superficial Femoral and Proximal Popliteal Arteries: One-Year Results of the MDT-2113 SFA Japan Randomized Trial. Journal of endovascular therapy : an official journal of the International Society of Endovascular Specialists. 2018;25(1):109-17.

30. Rastan A, McKinsey JF, Garcia LA, Rocha-Singh KJ, Jaff MR, Noory E, et al. One-Year Outcomes Following Directional Atherectomy of Infrapopliteal Artery Lesions: Subgroup Results of the Prospective, Multicenter DEFINITIVE LE Trial. Journal of endovascular therapy : an official journal of the International Society of Endovascular Specialists. 2015;22(6):839-46.

31. Lichtenberg M, von Bilderling P, Ranft J, Niemoller K, Grell H, Briner L, et al. Treatment of femoropopliteal atherosclerotic lesions using the ranger paclitaxel-coated balloon catheter: 12-month results from an all-comers registry. The Journal of cardiovascular surgery. 2018;59(1):45-50.

32. Harwood AE, Green J, Cayton T, Raza A, Wallace T, Carradice D, et al. A feasibility double-blind randomized placebo-controlled trial of extracorporeal shockwave therapy as a novel treatment for intermittent claudication. Journal of vascular surgery. 2018;67(2):514-21.e2.

33. Deloge C, Boesmans E, Van Damme H, Defraigne J-O. Revascularization of the superficial femoral artery with paclitaxel-coated balloon for claudication. Acta chirurgica Belgica. 2018;118(1):42-7.

34. Normahani P, Kwasnicki R, Bicknell C, Allen L, Jenkins MP, Gibbs R, et al. Wearable Sensor Technology Efficacy in Peripheral Vascular Disease (wSTEP): A Randomized Controlled Trial. Annals of surgery. 2018;268(6):1113-8.

35. Tew GA, Shalan A, Jordan AR, Cook L, Coleman ES, Fairhurst C, et al. Unloading shoes for intermittent claudication: a randomised crossover trial. BMC cardiovascular disorders. 2017;17(1):283.

36. McDermott MM, Ferrucci L, Tian L, Guralnik JM, Lloyd-Jones D, Kibbe MR, et al. Effect of Granulocyte-Macrophage Colony-Stimulating Factor With or Without Supervised Exercise on Walking Performance in Patients With Peripheral Artery Disease: The PROPEL Randomized Clinical Trial. JAMA. 2017;318(21):2089-98.

37. Muller-Hulsbeck S, Keirse K, Zeller T, Schroe H, Diaz-Cartelle J. Long-Term Results from the MAJESTIC Trial of the Eluvia Paclitaxel-Eluting Stent for Femoropopliteal Treatment: 3-Year Follow-up. Cardiovascular and interventional radiology. 2017;40(12):1832-8.

38. Park S-Y, Wong A, Son W-M, Pekas EJ. Effects of heated water-based versus land-based exercise training on vascular function in individuals with peripheral artery disease. Journal of applied physiology (Bethesda, Md : 1985). 2020;128(3):565-75.

39. Babber A, Ravikumar R, Onida S, Lane TRA, Davies AH. Effect of footplate neuromuscular electrical stimulation on functional and quality-of-life parameters in patients with peripheral artery disease: pilot, and subsequent randomized clinical trial. The British journal of surgery. 2020;107(4):355-63.

40. Park S-Y, Kwak Y-S, Pekas EJ. Impacts of aquatic walking on arterial stiffness, exercise tolerance, and physical function in patients with peripheral artery disease: a randomized clinical trial. Journal of applied physiology (Bethesda, Md : 1985). 2019;127(4):940-9.

41. Brenner IKM, Brown CA, Hains SJM, Tranmer J, Zelt DT, Brown PM. Low-Intensity Exercise Training Increases Heart Rate Variability in Patients With Peripheral Artery Disease. Biological research for nursing. 2020;22(1):24-33.

42. Murrow JR, Brizendine JT, Djire B, Young H-J, Rathbun S, Nilsson KR, Jr., et al. Near infrared spectroscopy-guided exercise training for claudication in peripheral arterial disease. European journal of preventive cardiology. 2019;26(5):471-80.

43. Teichgraber U, Lehmann T, Aschenbach R, Scheinert D, Zeller T, Brechtel K, et al. Drug-coated Balloon Angioplasty of Femoropopliteal Lesions Maintained Superior Efficacy over Conventional Balloon: 2-year Results of the Randomized EffPac Trial. Radiology. 2020;295(2):478-87.

44. Bosiers M, Setacci C, De Donato G, Torsello G, Silveira PG, Deloose K, et al. ZILVERPASS Study: ZILVER PTX Stent vs Bypass Surgery in Femoropopliteal Lesions. Journal of endovascular therapy : an official journal of the International Society of Endovascular Specialists. 2020;27(2):287-95.

45. Golzar J, Soga Y, Babaev A, Iida O, Kawasaki D, Bachinsky W, et al. Effectiveness and Safety of a Paclitaxel-Eluting Stent for Superficial Femoral Artery Lesions up to 190 mm: One-Year Outcomes of the Single-Arm IMPERIAL Long Lesion Substudy of the Eluvia Drug-Eluting Stent. Journal of endovascular therapy : an official journal of the International Society of Endovascular Specialists. 2020;27(2):296-303.

46. Galea Holmes MN, Weinman JA, Bearne LM. A randomized controlled feasibility trial of a home-based walking behavior-change intervention for people with intermittent claudication. Journal of vascular nursing : official publication of the Society for Peripheral Vascular Nursing. 2019;37(2):135-43.

47. Hotta K, Batchelor WB, Graven J, Dahya V, Noel TE, Ghai A, et al. Daily Passive Muscle Stretching Improves Flow-Mediated Dilation of Popliteal Artery and 6-minute Walk Test in Elderly Patients with Stable Symptomatic Peripheral Artery Disease. Cardiovascular revascularization medicine : including molecular interventions. 2019;20(8):642-8.

48. Chen Z, Guo W, Jiang W, Wang F, Fu W, Zou Y, et al. IN.PACT SFA Clinical Study Using the IN.PACT Admiral Drug-Coated Balloon in a Chinese Patient Population. Journal of endovascular therapy : an official journal of the International Society of Endovascular Specialists. 2019;26(4):471-8.

49. Hammer A, Moertl D, Schlager O, Matschuck M, Seidinger D, Koppensteiner R, et al. Effects of n-3 PUFA on endothelial function in patients with peripheral arterial disease: a randomised, placebo-controlled, double-blind trial. The British journal of nutrition. 2019;122(6):698-706.

50. Dagenais GR, Jung H, Bogaty P, Bosch J, Yusuf S, Gerstein HC. Effects of basal insulin glargine and omega-3 on lower limb arterial disease outcome in patients with dysglycaemia: An analysis of the Outcome Reduction with an Initial Glargine INtervention (ORIGIN) trial. Diabetes, obesity & metabolism. 2019;21(6):1502-5.

51. Gardner AW, Parker DE, Montgomery PS. Changes in vascular and inflammatory biomarkers after exercise rehabilitation in patients with symptomatic peripheral artery disease. Journal of vascular surgery. 2019;70(4):1280-90.

52. Liistro F, Angioli P, Porto I, Ducci K, Falsini G, Ventoruzzo G, et al. Drug-Eluting Balloon Versus Drug-Eluting Stent for Complex Femoropopliteal Arterial Lesions: The DRASTICO Study. Journal of the American College of Cardiology. 2019;74(2):205-15.

53. Laird JA, Schneider PA, Jaff MR, Brodmann M, Zeller T, Metzger DC, et al. Long-Term Clinical Effectiveness of a Drug-Coated Balloon for the Treatment of Femoropopliteal Lesions: Five-Year Outcomes from the IN.PACT SFA Randomized Trial. Circulation: Cardiovascular Interventions. 2019;12(6):e007702.

54. Imran HM, Hyder ON, Soukas PA. Efficacy and safety of adjunctive drug-coated balloon therapy in endovascular treatment of common femoral artery disease. Cardiovascular revascularization medicine : including molecular interventions. 2019;20(3):210-4.

55. Omarjee L, Le Pabic E, Custaud M-A, Fontaine C, Locher C, Renault A, et al. Effects of sildenafil on maximum walking time in patients with arterial claudication: The ARTERIOFIL study. Vascular pharmacology. 2019;118-119(101130615):106563.

56. Tepe G, Micari A, Keirse K, Zeller T, Scheinert D, Li P, et al. Drug-Coated Balloon Treatment for Femoropopliteal Artery Disease: The Chronic Total Occlusion Cohort in the IN.PACT Global Study. JACC Cardiovascular interventions. 2019;12(5):484-93.

57. Stabile E, Gerardi D, Magliulo F, Zhelev D, Chervenkoff V, Taeymans K, et al. One-Year Clinical Outcomes of the Legflow Drug-Coated Balloon for the Treatment of Femoropopliteal Occlusions Registry. Journal of endovascular therapy : an official journal of the International Society of Endovascular Specialists. 2019;26(1):26-30.

58. San Norberto EM, Flota CM, Fidalgo-Domingos L, Taylor JH, Vaquero C. Real-World Results of Supera Stent Implantation for Popliteal Artery Atherosclerotic Lesions: 3-Year Outcome. Annals of vascular surgery. 2020;62(avs, 8703941):397-405.

59. Khalili H, Jeon-Slaughter H, Armstrong EJ, Baskar A, Tejani I, Shammas NW, et al. Atherectomy in below-the-knee endovascular interventions: One-year outcomes from the XLPAD registry. Catheterization and cardiovascular interventions : official journal of the Society for Cardiac Angiography & Interventions. 2019;93(3):488-93.

60. Akerman AP, Thomas KN, van Rij AM, Body ED, Alfadhel M, Cotter JD. Heat therapy vs. supervised exercise therapy for peripheral arterial disease: a 12-wk randomized, controlled trial. American journal of physiology Heart and circulatory physiology. 2019;316(6):H1495-H506.

61. Balin M, Kivrak T. Effect of Repeated Remote Ischemic Preconditioning on Peripheral Arterial Disease in Patients Suffering from Intermittent Claudication. Cardiovascular therapeutics. 2019;2019(101319630):9592378.

62. Novakovic M, Krevel B, Rajkovic U, Vizintin Cuderman T, Jansa Trontelj K, Fras Z, et al. Moderate-pain versus pain-free exercise, walking capacity, and cardiovascular health in patients with peripheral artery disease. Journal of vascular surgery. 2019;70(1):148-56.

63. Bausback Y, Wittig T, Schmidt A, Zeller T, Bosiers M, Peeters P, et al. Drug-Eluting Stent Versus Drug-Coated Balloon Revascularization in Patients With Femoropopliteal Arterial Disease. Journal of the American College of Cardiology. 2019;73(6):667-79.

64. Russell KS, Yates DP, Kramer CM, Feller A, Mahling P, Colin L, et al. A randomized, placebo-controlled trial of canakinumab in patients with peripheral artery disease. Vascular medicine (London, England). 2019;24(5):414-21.

65. Tenore GC, D'Avino M, Caruso D, Buonomo G, Acampora C, Caruso G, et al. Effect of Annurca Apple Polyphenols on Intermittent Claudication in Patients With Peripheral Artery Disease. The American journal of cardiology. 2019;123(5):847-53.

66. Pellinger TK, Neighbors CB, Simmons GH. Acute Lower Leg Heating Increases Exercise Capacity in Patients With Peripheral Artery Disease. The Journal of cardiovascular nursing. 2019;34(2):130-3.

67. Iida O, Soga Y, Urasawa K, Saito S, Jaff MR, Wang H, et al. Drug-coated balloon versus uncoated percutaneous transluminal angioplasty for the treatment of atherosclerotic lesions in the superficial femoral and proximal popliteal artery: 2-year results of the MDT-2113 SFA Japan randomized trial. Catheterization and cardiovascular interventions : official journal of the Society for Cardiac Angiography & Interventions. 2019;93(4):664-72.

68. McDermott MM, Polonsky TS, Guralnik JM, Ferrucci L, Tian L, Zhao L, et al. Racial Differences in the Effect of Granulocyte Macrophage Colony-Stimulating Factor on Improved Walking Distance in Peripheral Artery Disease: The PROPEL Randomized Clinical Trial. Journal of the American Heart Association. 2019;8(2):e011001.

69. McDermott MM, Kibbe MR, Guralnik JM, Ferrucci L, Criqui MH, Domanchuk K, et al. Durability of Benefits From Supervised Treadmill Exercise in People With Peripheral Artery Disease. Journal of the American Heart Association. 2019;8(1):e009380.

70. Schroe H, Holden AH, Goueffic Y, Jansen SJ, Peeters P, Keirse K, et al. Stellarex drug-coated balloon for treatment of femoropopliteal arterial disease-The ILLUMENATE Global Study: 12-Month results from a prospective, multicenter, single-arm study. Catheterization and cardiovascular interventions : official journal of the Society for Cardiac Angiography & Interventions. 2018;91(3):497-504.

71. Gardner AW, Montgomery PS, Wang M. Minimal clinically important differences in treadmill, 6-minute walk, and patient-based outcomes following supervised and home-based exercise in peripheral artery disease. Vascular medicine (London, England). 2018;23(4):349-57.

72. Jakubseviciene E, Melinyte K, Kubilius R. A Novel, Individualized Exercise Program for Patients with Peripheral Arterial Disease Recovering from Bypass Surgery. International journal of environmental research and public health. 2019;16(12).

73. Collins TC, Lu L, Ahluwalia JS, Nollen NL, Sirard J, Marcotte R, et al. Efficacy of Community-Based Exercise Therapy Among African American Patients With Peripheral Artery Disease: A Randomized Clinical Trial. JAMA network open. 2019;2(2):e187959.

74. Perin EC, Murphy MP, March KL, Bolli R, Loughran J, Yang PC, et al. Evaluation of Cell Therapy on Exercise Performance and Limb Perfusion in Peripheral Artery Disease: The CCTRN PACE Trial (Patients With Intermittent Claudication Injected With ALDH Bright Cells). Circulation. 2017;135(15):1417-28.

75. Jia X, Zhang J, Zhuang B, Fu W, Wu D, Wang F, et al. Acotec Drug-Coated Balloon Catheter: Randomized, Multicenter, Controlled Clinical Study in Femoropopliteal Arteries: Evidence From the AcoArt I Trial. JACC Cardiovascular interventions. 2016;9(18):1941-9.

76. Ellul C, Gatt A. Transcutaneous calf-muscle electro-stimulation: A prospective treatment for diabetic claudicants? Diabetes & vascular disease research. 2016;13(6):442-4.

77. Harzand A, Vakili AA, Alrohaibani A, Abdelhamid SM, Gordon NF, Thiel J, et al. Rationale and design of a smartphone-enabled, home-based exercise program in patients with symptomatic peripheral arterial disease: The smart step randomized trial. Clinical Cardiology. 2020;43(6):537-45.

78. Woessner M, VanBruggen MD, Pieper CF, Sloane R, Kraus WE, Gow AJ, et al. Beet the Best? Circulation research. 2018;123(6):654-9.

79. Dopheide JF, Geissler P, Rubrech J, Trumpp A, Zeller GC, Daiber A, et al. Influence of exercise training on proangiogenic TIE-2 monocytes and circulating angiogenic cells in patients with peripheral arterial disease. Clinical research in cardiology : official journal of the German Cardiac Society. 2016;105(8):666-76.

80. Kohi MP, Brodmann M, Zeller T, Micari A, Baumgartner I, Wang H, et al. Sex-Related Differences in the Long-Term Outcomes of Patients with Femoropopliteal Arterial Disease Treated with the IN.PACT Drug-Coated Balloon in the IN.PACT SFA Randomized Controlled Trial: A Post Hoc Analysis. Journal of Vascular and Interventional Radiology. 2020;31(9):1410.

81. Bonaca MP, Wiviott SD, Zelniker TA, Mosenzon O, Bhatt DL, Leiter LA, et al. Dapagliflozin and Cardiac, Kidney and Limb Outcomes in Patients With and Without Peripheral Artery Disease in DECLARE-TIMI 58. Circulation. 2020((Bonaca) TIMI Study Group, Division of Cardiovascular Medicine, Brigham and Women's Hospital Heart and Harvard Medical School, Boston, MA; University of Colorado School of Medicine, Aurora, CO(Wiviott, Bhatt, Goodrich, Sabatine) TIMI Study Group, Division).

82. Rodriguez-Leyva D, Rodriguez-Portelles A, Weighell W, Guzman R, Maddaford TG, Pierce GN. The effects of dietary flaxseed on cardiac arrhythmias and claudication in patients with peripheral arterial disease. Canadian Journal of Physiology and Pharmacology. 2019;97(6):557-61.

83. Steiner S, Schmidt A, Zeller T, Tepe G, Thieme M, Maiwald L, et al. COMPARE: prospective, randomized, non-inferiority trial of high- vs. low-dose paclitaxel drug-coated balloons for femoropopliteal interventions. European heart journal. 2020;41(27):2541-52.

84. Zeller T, Brechtel K, Meyer DR, Noory E, Beschorner U, Albrecht T. Six-Month Outcomes From the First-in-Human, Single-Arm SELUTION Sustained-Limus-Release Drug-Eluting Balloon Trial in Femoropopliteal Lesions. Journal of Endovascular Therapy. 2020((Zeller, Noory) Department of Angiology, Universitats-Herzzentrum Freiburg-Bad Krozingen, Bad Krozingen, Germany(Brechtel) Ihre-Radiologen Berlin Gemeinschaftspraxis fur Radiologie, Berlin, Germany(Meyer) Department of Diagnostic and Interventional Radiol).

85. Monteiro DP, Ribeiro-Samora GA, Britto RR, Pereira DAG. Effects of modified aerobic training on muscle metabolism in individuals with peripheral arterial disease: a randomized clinical trial. Scientific reports. 2019;9(1):15966.

86. Lai Z, Zhang X, Shao J, Li K, Fang L, Xu L, et al. One-year results of drug-coated balloons for long and occlusive Femoropopliteal artery disease: A single-arm trial. BMC Cardiovascular Disorders. 2020;20(1):65.

87. Atkin L, Stephenson J, Ousey K. Feasibility study to evaluate cycloidal vibration therapy for the symptomatic treatment of intermittent claudication. Pilot and Feasibility Studies. 2019;5(1):133.

88. Laird JA, Schneider PA, Jaff MR, Brodmann M, Zeller T, Metzger DC, et al. Long-Term Clinical Effectiveness of a Drug-Coated Balloon for the Treatment of Femoropopliteal Lesions. Circulation Cardiovascular interventions. 2019;12(6):e007702.

89. Kostewicz MW, Kostewicz W. Impact of twelve weeks March field training on intermittent claudication in patients with peripheral artery disease after endovascular procedures. Postepy Rehabilitacji. 2019;33(1):15-21.

90. Bock J, Treichler D, Ueda K, Hughes W, Casey D. Inorganic nitrate supplementation improves exercise tolerance and calf vascular function in patients with peripheral artery disease. FASEB Journal. 2018;32(1 Supplement 1).

91. Bunte MC, Cohen DJ, Jaff MR, Gray WA, Magnuson EA, Li H, et al. Long-term clinical and quality of life outcomes after stenting of femoropopliteal artery stenosis: 3-year results from the STROLL study. Catheterization and Cardiovascular Interventions. 2018;92(1):106-14.

92. Bronas UG, Everett S, Steffen A, Briller J, Hannan M, Hernandez A, et al. Rhythmic Auditory Music Stimulation Enhances Walking Distance in Patients with Claudication: A Feasibility Study. Journal of Cardiopulmonary Rehabilitation and Prevention. 2018;38(4):E1-E5.

93. Miura T, Miyashita Y, Soga Y, Hozawa K, Doijiri T, Ikeda U, et al. Drug-eluting versus bare-metal stent implantation with or without cilostazol in the treatment of the superficial femoral artery: The debate in SFA study. Circulation: Cardiovascular Interventions. 2018;11(8):e006564.

94. Ali N, Gandotra V. Evaluation of efficacy of prostaglandin E-1 in peripheral arterial disease patients with intermittent claudication: A randomized double blind placebo controlled study. JK Science. 2017;19(2):95-8.

95. Schroeder H, Meyer DR, Lux B, Ruecker F, Martorana M, Miller LE, et al. A Pilot Study of Femoropopliteal Artery Revascularisation with a Low Dose Paclitaxel Coated Balloon: Is Predilatation Necessary? European Journal of Vascular and Endovascular Surgery. 2017;54(3):348-55.

96. Garcia L, Jaff M, Rocha-Singh K, Zeller T, McKinsey J. Acute Results of Directional Atherectomy for the Treatment of Claudication and Critical Limb Ischemia in the DEFINITIVE le Study. Vascular Disease Management. 2017;14(2):E21-E33.

97. Janas A, Buszman PP, Milewski KP, Wiernek S, Janas K, Pruski M, et al. Long-term outcomes after percutaneous lower extremity arterial interventions with atherectomy vs. Balloon angioplasty - Propensity score-matched registry. Circulation Journal. 2017;81(3):376-82.

98. Laird JR, Schneider PA, Tepe G, Brodmann M, Zeller T, Metzger C, et al. Durability of Treatment Effect Using a Drug-Coated Balloon for Femoropopliteal Lesions: 24-Month Results of IN.PACT SFA. Journal of the American College of Cardiology. 2015;66(21):2329-38.

99. De Haro J, Bleda S, Varela C, Esparza L, Acin F. Effect of bosentan on claudication distance and endothelium-dependent vasodilation in hispanic patients with peripheral arterial disease. American Journal of Cardiology. 2016;117(2):295-301.

100. Kinstner CM, Lammer J, Willfort-Ehringer A, Matzek W, Gschwandtner M, Javor D, et al. Paclitaxel-Eluting Balloon Versus Standard Balloon Angioplasty in In-Stent Restenosis of the Superficial Femoral and Proximal Popliteal Artery: 1-Year Results of the PACUBA Trial. JACC Cardiovascular interventions. 2016;9(13):1386-92.

101. Seenan C, McSwiggan S, Roche PA, Tan C-W, Mercer T, Belch JJF. Transcutaneous Electrical Nerve Stimulation Improves Walking Performance in Patients With Intermittent Claudication. The Journal of cardiovascular nursing. 2016;31(4):323-30.

102. Lammer J, Bosiers M, Deloose K, Schmidt A, Zeller T, Wolf F, et al. Bioresorbable Everolimus-Eluting Vascular Scaffold for Patients With Peripheral Artery Disease (ESPRIT I): 2-Year Clinical and Imaging Results. JACC Cardiovascular interventions. 2016;9(11):1178-87.

103. Nordanstig J, Taft C, Hensater M, Perlander A, Osterberg K, Jivegard L. Two-year results from a randomized clinical trial of revascularization in patients with intermittent claudication. The British journal of surgery. 2016;103(10):1290-9.

104. Muller-Hulsbeck S, Keirse K, Zeller T, Schroe H, Diaz-Cartelle J. Twelve-Month Results From the MAJESTIC Trial of the Eluvia Paclitaxel-Eluting Stent for Treatment of Obstructive Femoropopliteal Disease. Journal of endovascular therapy : an official journal of the International Society of Endovascular Specialists. 2016;23(5):701-7.

105. Scheinert D, Schmidt A, Zeller T, Muller-Hulsbeck S, Sixt S, Schroder H, et al. German Center Subanalysis of the LEVANT 2 Global Randomized Study of the Lutonix Drug-Coated Balloon in the Treatment of Femoropopliteal Occlusive Disease. Journal of endovascular therapy : an official journal of the International Society of Endovascular Specialists. 2016;23(3):409-16.

106. De Haro J, Bleda S, Gonzalez-Hidalgo C, Michel I, Acin F. Long-Term Effects of Bosentan on Cardiovascular Events in Hispanic Patients with Intermittent Claudication: Four-Year Follow-up of the CLAU Trial : The CLAU Randomized Trial Long-Term Outcome. American journal of cardiovascular drugs : drugs, devices, and other interventions. 2019;19(2):203-9.

107. Ahmed KMT, Hernon S, Mohamed S, Tubassum M, Newell M, Walsh SR. Remote Ischemic Pre-conditioning in the Management of Intermittent Claudication: A Pilot Randomized Controlled Trial. Annals of vascular surgery. 2019;55(avs, 8703941):122-30.

108. Jordan AR, Tew GA, Hutchins SW, Shalan A, Cook L, Thompson A. Three-curve rocker-soled shoes and gait adaptations to intermittent claudication pain: A randomised crossover trial. Gait & posture. 2019;67(9416830, dcm):31-6.

109. Xu Y, Jia X, Zhang J, Zhuang B, Fu W, Wu D, et al. Drug-Coated Balloon Angioplasty Compared With Uncoated Balloons in the Treatment of 200 Chinese Patients With Severe Femoropopliteal Lesions: 24-Month Results of AcoArt I. JACC Cardiovascular interventions. 2018;11(23):2347-53.

110. Scheinert D, Micari A, Brodmann M, Tepe G, Peeters P, Jaff MR, et al. Drug-Coated Balloon Treatment for Femoropopliteal Artery Disease. Circulation Cardiovascular interventions. 2018;11(10):e005654.

111. Kropielnicka K, Dziubek W, Bulinska K, Stefanska M, Wojcieszczyk-Latos J, Jasinski R, et al. Influence of the Physical Training on Muscle Function and Walking Distance in Symptomatic Peripheral Arterial Disease in Elderly. BioMed research international. 2018;2018(101600173):1937527.

112. Ansel GM, Brodmann M, Keirse K, Micari A, Jaff MR, Rocha-Singh K, et al. Drug-Coated Balloon Treatment of Femoropopliteal Lesions Typically Excluded From Clinical Trials: 12-Month Findings From the IN.PACT Global Study. Journal of endovascular therapy : an official journal of the International Society of Endovascular Specialists. 2018;25(6):673-82.

113. Jeon-Slaughter H, Khalili H, Tsai S, Armstrong EJ, Shammas NW, Jawaid O, et al. Comparative Effectiveness Study of Drug-Eluting and Bare-Metal Peripheral Artery Stents in Endovascular Femoropopliteal Artery Revascularization. The Journal of invasive cardiology. 2018;30(10):373-9.

114. Gray WA, Keirse K, Soga Y, Benko A, Babaev A, Yokoi Y, et al. A polymer-coated, paclitaxel-eluting stent (Eluvia) versus a polymer-free, paclitaxel-coated stent (Zilver PTX) for endovascular femoropopliteal intervention (IMPERIAL): a randomised, non-inferiority trial. Lancet (London, England). 2018;392(10157):1541-51.

115. Afzelius P, Molsted S, Tarnow L. Intermittent vacuum treatment with VacuMed does not improve peripheral artery disease or walking capacity in patients with intermittent claudication. Scandinavian journal of clinical and laboratory investigation. 2018;78(6):456-63.

116. Bock JM, Treichler DP, Norton SL, Ueda K, Hughes WE, Casey DP. Inorganic nitrate supplementation enhances functional capacity and lower-limb microvascular reactivity in patients with peripheral artery disease. Nitric oxide : biology and chemistry. 2018;80(c5f, 9709307):45-51.

117. Duscha BD, Piner LW, Patel MP, Crawford LE, Jones WS, Patel MR, et al. Effects of a 12-Week mHealth Program on FunctionalCapacity and Physical Activity in Patients With PeripheralArtery Disease. The American journal of cardiology. 2018;122(5):879-84.

118. Lindeman JHN, Zwaginga JJ, Kallenberg-Lantrua G, van Wissen RC, Schepers A, van Bockel HJ, et al. No Clinical Benefit of Intramuscular Delivery of Bone Marrow-derived Mononuclear Cells in Nonreconstructable Peripheral Arterial Disease: Results of a Phase-III Randomized-controlled Trial. Annals of surgery. 2018;268(5):756-61.

119. Deev R, Plaksa I, Bozo I, Mzhavanadze N, Suchkov I, Chervyakov Y, et al. Results of 5-year follow-up study in patients with peripheral artery disease treated with PL-VEGF165 for intermittent claudication. Therapeutic advances in cardiovascular disease. 2018;12(9):237-46.

120. Zeller T, Langhoff R, Rocha-Singh KJ, Jaff MR, Blessing E, Amann-Vesti B, et al. Directional Atherectomy Followed by a Paclitaxel-Coated Balloon to Inhibit Restenosis and Maintain Vessel Patency: Twelve-Month Results of the DEFINITIVE AR Study. Circulation Cardiovascular interventions. 2017;10(9).

121. Krankenberg H, Zeller T, Ingwersen M, Schmalstieg J, Gissler HM, Nikol S, et al. Self-Expanding Versus Balloon-Expandable Stents for Iliac Artery Occlusive Disease: The Randomized ICE Trial. JACC Cardiovascular interventions. 2017;10(16):1694-704.

122. de Boer SW, van den Heuvel DAF, de Vries-Werson DAB, Vos JA, Fioole B, Vroegindeweij D, et al. Short-term Results of the RAPID Randomized Trial of the Legflow Paclitaxel-Eluting Balloon With Supera Stenting vs Supera Stenting Alone for the Treatment of Intermediate and Long Superficial Femoral Artery Lesions. Journal of endovascular therapy : an official journal of the International Society of Endovascular Specialists. 2017;24(6):783-92.

123. Ott I, Cassese S, Groha P, Steppich B, Voll F, Hadamitzky M, et al. ISAR-PEBIS (Paclitaxel-Eluting Balloon Versus Conventional Balloon Angioplasty for In-Stent Restenosis of Superficial Femoral Artery): A Randomized Trial. Journal of the American Heart Association. 2017;6(7).

124. Krishnan P, Faries P, Niazi K, Jain A, Sachar R, Bachinsky WB, et al. Stellarex Drug-Coated Balloon for Treatment of Femoropopliteal Disease: Twelve-Month Outcomes From the Randomized ILLUMENATE Pivotal and Pharmacokinetic Studies. Circulation. 2017;136(12):1102-13.

125. Tepe G, Gogebakan O, Redlich U, Tautenhahn J, Ricke J, Halloul Z, et al. Angiographic and Clinical Outcomes After Treatment of Femoro-Popliteal Lesions with a Novel Paclitaxel-Matrix-Coated Balloon Catheter. Cardiovascular and interventional radiology. 2017;40(10):1535-44.

126. Embrey DG, Alon G, Brandsma BA, Vladimir F, Silva A, Pflugeisen BM, et al. Functional electrical stimulation improves quality of life by reducing intermittent claudication. International journal of cardiology. 2017;243(gqw, 8200291):454-9.

127. van Schaardenburgh M, Wohlwend M, Rognmo O, Mattsson EJR. Exercise in claudicants increase or decrease walking ability and the response relates to mitochondrial function. Journal of translational medicine. 2017;15(1):130.

128. Labrunee M, Boned A, Granger R, Bousquet M, Jordan C, Richard L, et al. Improved Walking Claudication Distance with Transcutaneous Electrical Nerve Stimulation: An Old Treatment with a New Indication in Patients with Peripheral Artery Disease. American journal of physical medicine & rehabilitation. 2015;94(11):941-9.

129. Rastan A, Krankenberg H, Baumgartner I, Blessing E, Muller-Hulsbeck S, Pilger E, et al. Stent placement vs. balloon angioplasty for popliteal artery treatment: two-year results of a prospective, multicenter, randomized trial. Journal of endovascular therapy : an official journal of the International Society of Endovascular Specialists. 2015;22(1):22-7.

130. Scheinert D, Schulte K-L, Zeller T, Lammer J, Tepe G. Paclitaxel-releasing balloon in femoropopliteal lesions using a BTHC excipient: twelve-month results from the BIOLUX P-I randomized trial. Journal of endovascular therapy : an official journal of the International Society of Endovascular Specialists. 2015;22(1):14-21.

131. Dopheide JF, Scheer M, Doppler C, Obst V, Stein P, Vosseler M, et al. Change of walking distance in intermittent claudication: impact on inflammation, oxidative stress and mononuclear cells: a pilot study. Clinical research in cardiology : official journal of the German Cardiac Society. 2015;104(9):751-63.

132. Murphy TP, Cutlip DE, Regensteiner JG, Mohler ER, 3rd, Cohen DJ, Reynolds MR, et al. Supervised exercise, stent revascularization, or medical therapy for claudication due to aortoiliac peripheral artery disease: the CLEVER study. Journal of the American College of Cardiology. 2015;65(10):999-1009.

133. Mays RJ, Hiatt WR, Casserly IP, Rogers RK, Main DS, Kohrt WM, et al. Community-based walking exercise for peripheral artery disease: An exploratory pilot study. Vascular medicine (London, England). 2015;20(4):339-47.

134. Schroeder H, Meyer D-R, Lux B, Ruecker F, Martorana M, Duda S. Two-year results of a low-dose drug-coated balloon for revascularization of the femoropopliteal artery: outcomes from the ILLUMENATE first-in-human study. Catheterization and cardiovascular interventions : official journal of the Society for Cardiac Angiography & Interventions. 2015;86(2):278-86.

135. Rocha-Singh KJ, Bosiers M, Schultz G, Jaff MR, Mehta M, Matsumura JS, et al. A single stent strategy in patients with lifestyle limiting claudication: 3-year results from the Durability II trial. Catheterization and cardiovascular interventions : official journal of the Society for Cardiac Angiography & Interventions. 2015;86(1):164-70.

136. Guirro ECdO, Guirro RRdJ, Dibai-Filho AV, Pascote SCS, Rodrigues-Bigaton D. Immediate effects of electrical stimulation, diathermy, and physical exercise on lower limb arterial blood flow in diabetic women with peripheral arterial disease: a randomized crossover trial. Journal of manipulative and physiological therapeutics. 2015;38(3):195-202.

137. Arao K, Yasu T, Endo Y, Funazaki T, Ota Y, Shimada K, et al. Effects of pitavastatin on walking capacity and CD34+/133+ cell number in patients with peripheral artery disease. Heart and vessels. 2017;32(10):1186-94.

138. Schroeder H, Werner M, Meyer D-R, Reimer P, Kruger K, Jaff MR, et al. Low-Dose Paclitaxel-Coated Versus Uncoated Percutaneous Transluminal Balloon Angioplasty for Femoropopliteal Peripheral Artery Disease: One-Year Results of the ILLUMENATE European Randomized Clinical Trial (Randomized Trial of a Novel Paclitaxel-Coated Pe. Circulation. 2017;135(23):2227-36.

139. Ott I, Cassese S, Groha P, Steppich B, Hadamitzky M, Ibrahim T, et al. Randomized Comparison of Paclitaxel-Eluting Balloon and Stenting Versus Plain Balloon Plus Stenting Versus Directional Atherectomy for Femoral Artery Disease (ISAR-STATH). Circulation. 2017;135(23):2218-26.

140. McDermott MM, Leeuwenburgh C, Guralnik JM, Tian L, Sufit R, Zhao L, et al. Effect of Resveratrol on Walking Performance in Older People With Peripheral Artery Disease: The RESTORE Randomized Clinical Trial. JAMA cardiology. 2017;2(8):902-7.

141. Chehuen M, Cucato GG, Carvalho CRF, Ritti-Dias RM, Wolosker N, Leicht AS, et al. Walking training at the heart rate of pain threshold improves cardiovascular function and autonomic regulation in intermittent claudication: A randomized controlled trial. Journal of science and medicine in sport. 2017;20(10):886-92.

142. Lindgren H. Primary stenting of the superficial femoral artery in intermittent claudication improves health-related quality of life, ABI, and walking distance: 12-month results of a randomized controlled multicenter trial. CardioVascular and Interventional Radiology. 2017;40(2 Supplement 1):S151-S2.

143. Kashyap VS, Lakin RO, Campos P, Allemang M, Kim A, Sarac TP, et al. The LargPAD Trial: Phase IIA evaluation of l-arginine infusion in patients with peripheral arterial disease. Journal of vascular surgery. 2017;66(1):187-94.

144. Oakley C, Spafford C, Beard JD. A Three Month Home Exercise Programme Augmented with Nordic Poles for Patients with Intermittent Claudication Enhances Quality of Life and Continues to Improve Walking Distance and Compliance After One Year. European journal of vascular and endovascular surgery : the official journal of the European Society for Vascular Surgery. 2017;53(5):704-9.

145. Van Schaardenburgh M, Wohlwend M, Rognmo O, Mattsson E. Calf raise exercise increases walking performance in patients with intermittent claudication. Journal of vascular surgery. 2017;65(5):1473-82.

146. Zen K, Takahara M, Iida O, Soga Y, Kawasaki D, Nanto S, et al. Drug-eluting stenting for femoropopliteal lesions, followed by cilostazol treatment, reduces stent restenosis in patients with symptomatic peripheral artery disease. Journal of vascular surgery. 2017;65(3):720-5.

147. Falkowski A, Bogacki H, Szemitko M. Assessment of mortality and factors affecting outcome of use of paclitaxel-coated stents and bare metal stents in femoropopliteal pad. Journal of Clinical Medicine. 2020;9(7):1-11.

148. Branch KR, Probstfield JL, Eikelboom JW, Bosch J, Maggioni AP, Cheng RK, et al. Rivaroxaban with or without aspirin in patients with heart failure and chronic coronary or peripheral artery disease the COMPASS trial. Circulation. 2019;140(7):529-37.

149. Villemur B, Thoreau V, Guinot M, Gailledrat E, Evra V, Vermorel C, et al. Short interval or continuous training programs to improve walking distance for intermittent claudication: Pilot study. Annals of Physical and Rehabilitation Medicine. 2020((Villemur, Thoreau, Evra) Department of Vascular Rehabilitation, Grenoble Alpes University Hospital, Grenoble 38433, France(Guinot, Gailledrat) Sports Medicine Department, Grenoble Alpes University Hospital, Grenoble 38433, France(Guinot) INSERM U1042, La).

150. Baker WB, Li Z, Schenkel SS, Chandra M, Busch DR, Englund EK, et al. Effects of exercise training on calf muscle oxygen extraction and blood flow in patients with peripheral artery disease. Journal of applied physiology (Bethesda, Md : 1985). 2017;123(6):1599-609.

151. Davis T, Ramaiah V, Niazi K, Martin Gissler H, Crabtree T. Safety and effectiveness of the Phoenix Atherectomy System in lower extremity arteries: Early and midterm outcomes from the prospective multicenter EASE study. Vascular. 2017;25(6):563-75.

152. Cavalcante BR, Ritti-Dias RM, Soares AHG, Lima AH, Correia MA, De Matos LD, et al. A Single Bout of Arm-crank Exercise Promotes Positive Emotions and Post-Exercise Hypotension in Patients with Symptomatic Peripheral Artery Disease. European journal of vascular and endovascular surgery : the official journal of the European Society for Vascular Surgery. 2017;53(2):223-8.

153. Thomas KN, van Rij AM, Lucas SJE, Cotter JD. Lower-limb hot-water immersion acutely induces beneficial hemodynamic and cardiovascular responses in peripheral arterial disease and healthy, elderly controls. American journal of physiology Regulatory, integrative and comparative physiology. 2017;312(3):R281-R91.

154. Hiatt WR, Fowkes FGR, Heizer G, Berger JS, Baumgartner I, Held P, et al. Ticagrelor versus Clopidogrel in Symptomatic Peripheral Artery Disease. The New England journal of medicine. 2017;376(1):32-40.

155. Cavalcante BR, Ritti-Dias RM, Germano Soares AH, Domingues WJR, Saes GF, Duarte FH, et al. Graduated Compression Stockings Does Not Decrease Walking Capacity and Muscle Oxygen Saturation during 6-Minute Walk Test in Intermittent Claudication Patients. Annals of vascular surgery. 2017;40(avs, 8703941):239-42.

156. Bague N, Julia P, Sauguet A, Pernes JM, Chatelard P, Garbe JF, et al. Femoropopliteal In-stent Restenosis Repair: Midterm Outcomes After Paclitaxel Eluting Balloon Use (PLAISIR Trial). European journal of vascular and endovascular surgery : the official journal of the European Society for Vascular Surgery. 2017;53(1):106-13.

157. Mazari FAK, Khan JA, Samuel N, Smith G, Carradice D, McCollum PC, et al. Long-term outcomes of a randomized clinical trial of supervised exercise, percutaneous transluminal angioplasty or combined treatment for patients with intermittent claudication due to femoropopliteal disease. The British journal of surgery. 2017;104(1):76-83.

158. Szymczak M, Oszkinis G, Majchrzycki M. The Impact of Walking Exercises and Resistance Training upon the Walking Distance in Patients with Chronic Lower Limb Ischaemia. BioMed research international. 2016;2016(101600173):7515238.

159. Schmidt A, Piorkowski M, Gorner H, Steiner S, Bausback Y, Scheinert S, et al. Drug-Coated Balloons for Complex Femoropopliteal Lesions: 2-Year Results of a Real-World Registry. JACC Cardiovascular interventions. 2016;9(7):715-24.

160. Dake MD, Ansel GM, Jaff MR, Ohki T, Saxon RR, Smouse HB, et al. Durable Clinical Effectiveness With Paclitaxel-Eluting Stents in the Femoropopliteal Artery: 5-Year Results of the Zilver PTX Randomized Trial. Circulation. 2016;133(15):1472-83.

161. Yokoi H, Ohki T, Kichikawa K, Nakamura M, Komori K, Nanto S, et al. Zilver PTX Post-Market Surveillance Study of Paclitaxel-Eluting Stents for Treating Femoropopliteal Artery Disease in Japan: 12-Month Results. JACC Cardiovascular interventions. 2016;9(3):271-7.

162. Chu YS, Li DX, Zhang M, Jiang TM. Trimetazidine hydrochloride as a new treatment for patients with peripheral vascular disease--an exploratory trial. European review for medical and pharmacological sciences. 2016;20(1):188-93.

163. Katsanos K, Spiliopoulos S, Diamantopoulos A, Siablis D, Karnabatidis D, Scheinert D. Wound Healing Outcomes and Health-Related Quality-of-Life Changes in the ACHILLES Trial: 1-Year Results From a Prospective Randomized Controlled Trial of Infrapopliteal Balloon Angioplasty Versus Sirolimus-Eluting Stenting in Patients With Ischemic Periph. JACC Cardiovascular interventions. 2016;9(3):259-67.

164. De Haro J, Bleda S, Varela C, Esparza L, Acin F, Bosentan Population-Based Randomized Trial for C, et al. Effect of Bosentan on Claudication Distance and Endothelium-Dependent Vasodilation in Hispanic Patients With Peripheral Arterial Disease. Am J Cardiol. 2016;117(2):295-301.

165. Lamberti N, Malagoni AM, Ficarra V, Basaglia N, Manfredini R, Zamboni P, et al. Structured Home-Based Exercise Versus Invasive Treatment: A Mission Impossible? A Pilot Randomized Study in Elderly Patients With Intermittent Claudication. Angiology. 2016;67(8):772-80.

166. Han DK, Faries PL, Chung C, Weaver MV, Tadros RO, Ting W, et al. Intermediate Outcomes of Femoropopliteal Stenting in Women: 3-Year Results of the DURABILITY II Trial. Annals of vascular surgery. 2016;30(avs, 8703941):110-7.

167. Grossman PM, Mohler ER, 3rd, Roessler BJ, Wilensky RL, Levine BL, Woo EY, et al. Phase I study of multi-gene cell therapy in patients with peripheral artery disease. Vascular medicine (London, England). 2016;21(1):21-32.

168. Delaney CL, Spark JI. A randomised controlled trial of two supervised exercise regimens and their impact on inflammatory burden in patients with intermittent claudication. Vascular. 2016;24(3):264-72.

169. Bulinska K, Kropielnicka K, Jasinski T, Wojcieszczyk-Latos J, Pilch U, Dabrowska G, et al. Nordic pole walking improves walking capacity in patients with intermittent claudication: a randomized controlled trial. Disability and rehabilitation. 2016;38(13):1318-24.

170. Robertson AJ, Struthers AD. A Randomized Controlled Trial of Allopurinol in Patients With Peripheral Arterial Disease. The Canadian journal of cardiology. 2016;32(2):190-6.

171. Dipnarine K, Barak S, Martinez CA, Carmeli E, Stopka CB. Pain-free treadmill exercise for patients with intermittent claudication: Are there gender differences? Vascular. 2016;24(3):304-14.

172. Mauer K, Exaire JE, Stoner JA, Saucedo JF, Montgomery PS, Gardner AW. Effect of exercise training on clot strength in patients with peripheral artery disease and intermittent claudication: An ancillary study. SAGE open medicine. 2015;3(101624744):2050312115575938.

173. Alvarez OM, Wendelken ME, Markowitz L, Comfort C. Effect of High-pressure, Intermittent Pneumatic Compression for the Treatment of Peripheral Arterial Disease and Critical Limb Ischemia in Patients Without a Surgical Option. Wounds : a compendium of clinical research and practice. 2015;27(11):293-301.

174. Fakhry F, Spronk S, van der Laan L, Wever JJ, Teijink JAW, Hoffmann WH, et al. Endovascular Revascularization and Supervised Exercise for Peripheral Artery Disease and Intermittent Claudication: A Randomized Clinical Trial. JAMA. 2015;314(18):1936-44.

175. Zeller T, Beschorner U, Pilger E, Bosiers M, Deloose K, Peeters P, et al. Paclitaxel-Coated Balloon in Infrapopliteal Arteries: 12-Month Results From the BIOLUX P-II Randomized Trial (BIOTRONIK'S-First in Man study of the Passeo-18 LUX drug releasing PTA Balloon Catheter vs. the uncoated Passeo-18 PTA balloon catheter in subjec. JACC Cardiovascular interventions. 2015;8(12):1614-22.

176. Babaev A, Zavlunova S, Attubato MJ, Martinsen BJ, Mintz GS, Maehara A. Orbital Atherectomy Plaque Modification Assessment of the Femoropopliteal Artery Via Intravascular Ultrasound (TRUTH Study). Vascular and endovascular surgery. 2015;49(7):188-94.

177. Rastan A. One-year outcomes following directional atherectomy of infrapopliteal artery lesions: Sub-group results of the prospective, multi-center definitive le trial. Vasa - European Journal of Vascular Medicine. 2015;44(Supplement 88):46.

178. Gommans LNM, Scheltinga MRM, van Sambeek MRHM, Maas AHEM, Bendermacher BLW, Teijink JAW. Gender differences following supervised exercise therapy in patients with intermittent claudication. Journal of vascular surgery. 2015;62(3):681-8.

179. Schulte K-L, Pilger E, Schellong S, Tan KT, Baumann F, Langhoff R, et al. Primary Self-EXPANDing Nitinol Stenting vs Balloon Angioplasty With Optional Bailout Stenting for the Treatment of Infrapopliteal Artery Disease in Patients With Severe Intermittent Claudication or Critical Limb Ischemia (EXPAND Study). Journal of endovascular therapy : an official journal of the International Society of Endovascular Specialists. 2015;22(5):690-7.

180. da Silva ND, Jr., Roseguini BT, Chehuen M, Fernandes T, Mota GF, Martin PKM, et al. Effects of oral N-acetylcysteine on walking capacity, leg reactive hyperemia, and inflammatory and angiogenic mediators in patients with intermittent claudication. American journal of physiology Heart and circulatory physiology. 2015;309(5):H897-905.

181. Rosenfield K, Jaff MR, White CJ, Rocha-Singh K, Mena-Hurtado C, Metzger DC, et al. Trial of a Paclitaxel-Coated Balloon for Femoropopliteal Artery Disease. The New England journal of medicine. 2015;373(2):145-53.

182. Delagarde H, Ouadraougo N, Grall S, Macchi L, Roy P-M, Abraham P, et al. Remote ischaemic preconditioning in intermittent claudication. Archives of cardiovascular diseases. 2015;108(10):472-9.

183. McDermott MM, Guralnik JM, Criqui MH, Ferrucci L, Liu K, Spring B, et al. Unsupervised exercise and mobility loss in peripheral artery disease: a randomized controlled trial. Journal of the American Heart Association. 2015;4(5).

184. Garcia L, Jaff MR, Metzger C, Sedillo G, Pershad A, Zidar F, et al. Wire-Interwoven Nitinol Stent Outcome in the Superficial Femoral and Proximal Popliteal Arteries: Twelve-Month Results of the SUPERB Trial. Circulation Cardiovascular interventions. 2015;8(5).

185. Gernigon M, Le Faucheur A, Fradin D, Noury-Desvaux B, Landron C, Mahe G, et al. Global positioning system use in the community to evaluate improvements in walking after revascularization: a prospective multicenter study with 6-month follow-up in patients with peripheral arterial disease. Medicine. 2015;94(18):e838.

186. Tew GA, Humphreys L, Crank H, Hewitt C, Nawaz S, Al-Jundi W, et al. The development and pilot randomised controlled trial of a group education programme for promoting walking in people with intermittent claudication. Vascular medicine (London, England). 2015;20(4):348-57.

187. Dippel EJ, Makam P, Kovach R, George JC, Patlola R, Metzger DC, et al. Randomized controlled study of excimer laser atherectomy for treatment of femoropopliteal in-stent restenosis: initial results from the EXCITE ISR trial (EXCImer Laser Randomized Controlled Study for Treatment of FemoropopliTEal In-Stent Restenosis). JACC Cardiovascular interventions. 2015;8(1 Pt A):92-101.

188. Fokkenrood HJP, Lauret GJ, Verhofstad N, Bendermacher BLW, Scheltinga MRM, Teijink JAW. The effect of supervised exercise therapy on physical activity and ambulatory activities in patients with intermittent claudication. European journal of vascular and endovascular surgery : the official journal of the European Society for Vascular Surgery. 2015;49(2):184-91.

189. Tepe G, Laird J, Schneider P, Brodmann M, Krishnan P, Micari A, et al. Drug-coated balloon versus standard percutaneous transluminal angioplasty for the treatment of superficial femoral and popliteal peripheral artery disease: 12-month results from the IN.PACT SFA randomized trial. Circulation. 2015;131(5):495-502.

190. Lammer J, Zeller T, Hausegger KA, Schaefer PJ, Gschwendtner M, Mueller-Huelsbeck S, et al. Sustained benefit at 2 years for covered stents versus bare-metal stents in long SFA lesions: the VIASTAR trial. Cardiovascular and interventional radiology. 2015;38(1):25-32.

191. Gray WA, Feiring A, Cioppi M, Hibbard R, Gray B, Khatib Y, et al. S.M.A.R.T. self-expanding nitinol stent for the treatment of atherosclerotic lesions in the superficial femoral artery (STROLL): 1-year outcomes. Journal of vascular and interventional radiology : JVIR. 2015;26(1):21-8.

192. Bo E, Bergland A, Stranden E, Jorgensen JJ, Sandbaek G, Grotta OJ, et al. Effects of 12 Weeks of Supervised Exercise After Endovascular Treatment: A Randomized Clinical Trial. Physiotherapy research international : the journal for researchers and clinicians in physical therapy. 2015;20(3):147-57.

193. Vlajinac H, Marinkovic J, Tanaskovic S, Kocev N, Radak D, Davidovic D, et al. Quality of life after peripheral bypass surgery: a 1 year follow-up. Wiener klinische Wochenschrift. 2015;127(5-6):210-7.

194. Delaney CL, Miller MD, Allan RB, Spark JI. The impact of different supervised exercise regimens on endothelial function in patients with intermittent claudication. Vascular. 2015;23(6):561-9.

195. Hammer A, Koppensteiner R, Steiner S, Niessner A, Goliasch G, Gschwandtner M, et al. Dark chocolate and vascular function in patients with peripheral artery disease: a randomized, controlled cross-over trial. Clinical hemorheology and microcirculation. 2015;59(2):145-53.

196. Prevost A, Lafitte M, Pucheu Y, Couffinhal T, on behalf the Cet. Education and home based training for intermittent claudication: functional effects and quality of life. European journal of preventive cardiology. 2015;22(3):373-9.

197. Powell RJ, Jaff MR, Schroe H, Benko A, Diaz-Cartelle J, Muller-Hulsbeck S. Stent placement in the superficial femoral and proximal popliteal arteries with the innova self-expanding bare metal stent system. Catheterization and cardiovascular interventions : official journal of the Society for Cardiac Angiography & Interventions. 2017;89(6):1069-77.

198. Rundback JH, Peeters P, George JC, Jaff MR, Faries PL. Results From the VISIBILITY Iliac Study: Primary and Cohort Outcomes at 9 Months. Journal of endovascular therapy : an official journal of the International Society of Endovascular Specialists. 2017;24(3):342-8.

199. Derosa G, D'Angelo A, Romano D, Maffioli P. Evaluation of the Effects of Mesoglycan on Some Markers of Endothelial Damage and Walking Distance in Diabetic Patients with Peripheral Arterial Disease. International journal of molecular sciences. 2017;18(3).

200. Girold S, Rousseau J, Le Gal M, Coudeyre E, Le Henaff J. Nordic walking versus walking without poles for rehabilitation with cardiovascular disease: Randomized controlled trial. Annals of physical and rehabilitation medicine. 2017;60(4):223-9.

201. Liao C-J, Song S-H, Li T, Zhang Y, Zhang W-d. Randomized controlled trial of orchid drug-coated balloon versus standard percutaneous transluminal angioplasty for treatment of femoropopliteal artery in-stent restenosis. International angiology : a journal of the International Union of Angiology. 2019;38(5):365-71.

202. Enzmann FK, Nierlich P, Aspalter M, Hitzl W, Dabernig W, Holzenbein T, et al. Nitinol Stent Versus Bypass in Long Femoropopliteal Lesions: 2-Year Results of a Randomized Controlled Trial. JACC Cardiovascular interventions. 2019;12(24):2541-9.

203. Rundback J, Chandra P, Brodmann M, Weinstock B, Sedillo G, Cawich I, et al. Novel laser-based catheter for peripheral atherectomy: 6-month results from the Eximo Medical B-Laser TM IDE study. Catheterization and cardiovascular interventions : official journal of the Society for Cardiac Angiography & Interventions. 2019;94(7):1010-7.

204. Ahmad AM, Abdel-Aziz HA. Laser acupuncture for claudication symptoms in peripheral artery disease - Does it work? A randomized trial. Hong Kong physiotherapy journal : official publication of the Hong Kong Physiotherapy Association Limited = Wu li chih liao. 2022;42(1):31-40.

205. Sachar R, Soga Y, Ansari MM, Kozuki A, Lopez L, Brodmann M, et al. 1-Year Results From the RANGER II SFA Randomized Trial of the Ranger Drug-Coated Balloon. JACC Cardiovascular interventions. 2021;14(10):1123-33.

206. Iida O, Fujihara M, Kawasaki D, Mori S, Yokoi H, Miyamoto A, et al. 24-Month Efficacy and Safety Results from Japanese Patients in the IMPERIAL Randomized Study of the Eluvia Drug-Eluting Stent and the Zilver PTX Drug-Coated Stent. Cardiovascular and interventional radiology. 2021;44(9):1367-74.

207. Monroe JC, Song Q, Emery MS, Hirai DM, Motaganahalli RL, Roseguini BT. Acute effects of leg heat therapy on walking performance and cardiovascular and inflammatory responses to exercise in patients with peripheral artery disease. Physiological reports. 2021;8(24):e14650.

208. Shishehbor MH, Griffin J, Crowder Wt, Kucher T, Leon LR, Jr., McClure JM, 2nd, et al. Acute Real-World Outcomes From the Phoenix Post-Approval Registry. The Journal of invasive cardiology. 2022;34(1):E1-E7.

209. Bertges DJ, Smith L, Scully RE, Wyers M, Eldrup-Jorgensen J, Suckow B, et al. A multicenter, prospective randomized trial of negative pressure wound therapy for infrainguinal revascularization with a groin incision. Journal of vascular surgery. 2021;74(1):257-67.e1.

210. Han A, Lee T, Lee J, Song S-W, Lee S-S, Jung IM, et al. A multicenter, randomized, open-labelled, non-inferiority trial of sustained-release sarpogrelate versus clopidogrel after femoropopliteal artery intervention. Scientific reports. 2023;13(1):2502.

211. Ni L, Ye W, Zhang L, Jin X, Shu C, Jiang JS, et al. A Multicenter Randomized Trial Assessing ZENFlow Carrier-Free Drug-Coated Balloon for the Treatment of Femoropopliteal Artery Lesions. Frontiers in Cardiovascular Medicine. 2022;9((Ni, Ye, Liu) Department of Vascular Surgery, Peking Union Medical College Hospital, Beijing, China(Zhang) Department of Vascular Surgery, Renji Hospital Affiliated to Shanghai Jiaotong University School of Medicine, Shanghai, China(Jin) Department of Vas):821672.

212. Therasse E, Benko A, Brodmann M, Hadziomerovic A. A Multinational, Single-Arm Pivotal Study Assessing the Performance of the SoundBite Crossing System for Peripheral Chronic Total Occlusions (The PROSPECTOR Study). Journal of vascular and interventional radiology : JVIR. 2022;33(1):50-9.

213. Hiatt WR, Hess CN, Bonaca MP, Kavanagh S, Patel MR, Baumgartner I, et al. Ankle-Brachial Index for Risk Stratification in Patients With Symptomatic Peripheral Artery Disease With and Without Prior Lower Extremity Revascularization: Observations From the EUCLID Trial. Circulation Cardiovascular interventions. 2021;14(7):e009871.

214. Goueffic Y, Sauguet A, Desgranges P, Feugier P, Rosset E, Ducasse E, et al. A Polymer-Free Paclitaxel-Eluting Stent Versus a Bare-Metal Stent for De Novo Femoropopliteal Lesions: The BATTLE Trial. JACC Cardiovascular interventions. 2020;13(4):447-57.

215. Saaya S, Osipova O, Gostev A, Rabtsun A, Starodubtsev V, Cheban A, et al. A prospective randomized trial on endovascular recanalization with stenting versus remote endarterectomy for the superficial femoral artery total occlusive lesions. Journal of vascular surgery. 2022;76(1):158-64.

216. Hoel H, Pettersen EM, Hoiseth LO, Mathiesen I, Seternes A, Hisdal J. A randomized controlled trial of treatment with intermittent negative pressure for intermittent claudication. Journal of vascular surgery. 2021;73(5):1750-8.e1.

217. Guo J, Guo L, Cui S, Dardik A, Liu Y, Tong Z, et al. A Retrospective Comparative Study of Twelve-Month Clinical Outcomes for Drug-Coating Balloon Angioplasty and Stent Implantation in Treating Patients with Popliteal Obstructive Lesions. Cardiovascular and interventional radiology. 2021;44(3):361-7.

218. Ren H, Liu J, Zhang J, Zhuang B, Fu W, Wu D, et al. Association between post-balloon angioplasty dissection and primary patency in complex femoropopliteal artery disease: 2-year clinical outcomes of the AcoArt I trial. The Journal of international medical research. 2021;49(4):3000605211006546.

219. Rymer JA, Mulder H, Smolderen KG, Hiatt WR, Conte MS, Berger JS, et al. Association of Health Status Scores With Cardiovascular and Limb Outcomes in Patients With Symptomatic Peripheral Artery Disease: Insights From the EUCLID (Examining Use of Ticagrelor in Symptomatic Peripheral Artery Disease) Trial. Journal of the American Heart Association. 2020;9(19):e016573.

220. Rastan A, Brodmann M, Bohme T, Macharzina R, Noory E, Beschorner U, et al. Atherectomy and Drug-Coated Balloon Angioplasty for the Treatment of Long Infrapopliteal Lesions: A Randomized Controlled Trial. Circulation Cardiovascular interventions. 2021;14(6):e010280.

221. Shammas NW, Shammas GA, Halupnik G, Fedele N, Comp K, Taleb E-M, et al. Auryon Laser in Peripheral Arterial Interventions: A Single-Center Experience (Auryon-SCE). The Journal of invasive cardiology. 2022;34(6):E428-E32.

222. De Beaufort LM, Nasr B, Corvec TL, Brisard L, Guyomarc'h B, Fellah I, et al. Automated Image Fusion Guidance during Endovascular Aorto-Iliac Procedures: A Randomized Controlled Pilot Study. Annals of vascular surgery. 2021;75(avs, 8703941):86-93.

223. Yoshioka N, Takagi K, Morita Y, Kanzaki Y, Nagai H, Watanabe N, et al. Bleeding events and mid-term mortality in the patients undergoing endovascular interventions for peripheral artery disease of the lower limbs based on the academic research consortium high bleeding risk criteria. Heart and vessels. 2021;36(9):1336-49.

224. Sami F, Ranka S, Lippmann M, Weiford B, Hance K, Whitman B, et al. Cardiac rehabilitation in patients with peripheral arterial disease after revascularization. Vascular. 2021;29(3):350-4.

225. Siercke M, Jorgensen LP, Missel M, Thygesen LC, Moller SP, Sillesen H, et al. Cardiovascular Rehabilitation Increases Walking Distance in Patients With Intermittent Claudication. Results of the CIPIC Rehab Study: A Randomised Controlled Trial. Journal of Vascular Surgery. 2021;74(6):2118.

226. Weissler EH, Narcisse DI, Rymer JA, Armstrong EJ, Secemsky E, Gray WA, et al. Characteristics and Outcomes of Patients With Diabetes Mellitus Undergoing Peripheral Vascular Intervention for Infrainguinal Symptomatic Peripheral Artery Disease. Vascular and endovascular surgery. 2021;55(2):124-34.

227. Miura T, Miyashita Y, Hozawa K, Doijiri T, Kato T, Hayakawa N, et al. Cilostazol effectiveness in reducing drug-coated stent restenosis in the superficial femoral artery: The ZERO study. PloS one. 2022;17(7):e0270992.

228. Lindholt JS, Sogaard R. Clinical Benefit, Harm, and Cost Effectiveness of Screening Men for Peripheral Artery Disease: A Markov Model Based on the VIVA Trial. European journal of vascular and endovascular surgery : the official journal of the European Society for Vascular Surgery. 2021;61(6):971-9.

229. Spoorendonk JA, Briere JB, Bowrin K, Millier A, Coppens M, Tempelaar S, et al. Clinical implications and cost-effectiveness analysis of rivaroxaban in patients with coronary artery disease or peripheral arterial disease in the Netherlands. Journal of medical economics. 2021;24(1):1231-9.

230. Yoshioka N, Tokuda T, Koyama A, Yamada T, Nishikawa R, Shimamura K, et al. Clinical outcomes and predictors of restenosis in patients with femoropopliteal artery disease treated using polymer-coated paclitaxel-eluting stents or drug-coated balloons. Heart and Vessels. 2022;37(4):555-66.

231. Deloose K, Bosiers M, Peeters P, Verbist J, Maene L, Beelen R, et al. Combining the Passeo-18 Lux Drug-Coated Balloon and the Pulsar-18 Bare Metal Stent: 12- and 24-Month Outcomes of the BIOLUX 4EVER Investigator-Initiated Trial. Journal of endovascular therapy : an official journal of the International Society of Endovascular Specialists. 2020;27(6):936-45.

232. Sun G, Liu J, Jia S, Zhang J, Zhuang B, Jia X, et al. Comparison of drug-coated balloon angioplasty versus uncoated balloon angioplasty in treatment of total occlusions with severe femoropopliteal lesions: An additional analysis from the AcoArt I study. Vascular. 2021;29(3):340-9.

233. van Reijen NS, van Dieren S, Frans FA, Reekers JA, Metz R, Buscher HCJL, et al. Cost Effectiveness of Endovascular Revascularisation vs. Exercise Therapy for Intermittent Claudication Due to Iliac Artery Obstruction. European journal of vascular and endovascular surgery : the official journal of the European Society for Vascular Surgery. 2022;63(3):430-7.

234. Djerf H, Svensson M, Nordanstig J, Gottsater A, Falkenberg M, Lindgren H. Cost Effectiveness of Primary Stenting in the Superficial Femoral Artery for Intermittent Claudication: Two Year Results of a Randomised Multicentre Trial. Journal of Vascular Surgery. 2021;74(5):1767.

235. Iwai T, Yamaguchi T, Ueshima D, Tobita K, Mizuno A, Fujimoto Y, et al. Differences in major limb outcomes by indication for lower extremity endovascular revascularization in patients receiving hemodialysis. Heart and vessels. 2023;38(4):488-96.

236. Babaev A, Halista M, Bakirova Z, Avtushka V, Matsumura M, Maehara A. Directional versus orbital atherectomy of femoropopliteal artery lesions: Angiographic and intravascular ultrasound outcomes. Catheterization and cardiovascular interventions : official journal of the Society for Cardiac Angiography & Interventions. 2022;100(4):687-95.

237. Goueffic Y, Pin JL, Sabatier J, Alimi Y, Steinmetz E, Magnan P-E, et al. Editor's Choice - A Cost Effectiveness Analysis of Outpatient versus Inpatient Hospitalisation for Lower Extremity Arterial Disease Endovascular Revascularisation in France: A Randomised Controlled Trial. European journal of vascular and endovascular surgery : the official journal of the European Society for Vascular Surgery. 2021;61(3):447-55.

238. Starodubtsev V, Mitrofanov V, Ignatenko P, Gostev A, Preece R, Rabtsun A, et al. Editor's Choice - Hybrid vs. Open Surgical Reconstruction for Iliofemoral Occlusive Disease: A Prospective Randomised Trial. European journal of vascular and endovascular surgery : the official journal of the European Society for Vascular Surgery. 2022;63(4):557-65.

239. Koelemay MJW, van Reijen NS, van Dieren S, Frans FA, Vermeulen EJG, Buscher HCJL, et al. Editor's Choice - Randomised Clinical Trial of Supervised Exercise Therapy vs. Endovascular Revascularisation for Intermittent Claudication Caused by Iliac Artery Obstruction: The SUPER study. European journal of vascular and endovascular surgery : the official journal of the European Society for Vascular Surgery. 2022;63(3):421-9.

240. Sandberg A, Back M, Cider A, Jivegard L, Sigvant B, Wittboldt S, et al. Effectiveness of supervised exercise, home-based exercise or walk advice strategies on walking performance and muscle endurance in patients with intermittent claudication (SUNFIT trial)-a randomized clinical trial. European journal of cardiovascular nursing. 2022(101128793).

241. Bearne LM, Volkmer B, Peacock J, Sekhon M, Fisher G, Galea Holmes MN, et al. Effect of a Home-Based, Walking Exercise Behavior Change Intervention vs Usual Care on Walking in Adults With Peripheral Artery Disease: The MOSAIC Randomized Clinical Trial. JAMA. 2022;327(14):1344-55.

242. Domingues WJR, Ritti-Dias RM, Cucato GG, Wolosker N, Zerati AE, Puech-Leao P, et al. Effect of Creatine Supplementation on Functional Capacity and Muscle Oxygen Saturation in Patients with Symptomatic Peripheral Arterial Disease: A Pilot Study of a Randomized, Double-Blind Placebo-Controlled Clinical Trial. Nutrients. 2021;13(1).

243. McDermott MM, Spring B, Tian L, Treat-Jacobson D, Ferrucci L, Lloyd-Jones D, et al. Effect of Low-Intensity vs High-Intensity Home-Based Walking Exercise on Walk Distance in Patients With Peripheral Artery Disease: The LITE Randomized Clinical Trial. JAMA. 2021;325(13):1266-76.

244. Bohme T, Beschorner U, Noory E, Molitor M, Nuhrenberg T, Neumann F-J, et al. Effect of Paclitaxel Drug-Coated Balloon Angioplasty of Infrapopliteal Lesions on Mortality. Texas Heart Institute journal. 2022;49(6).

245. Singh S, Singh G. Effect of Pentoxifylline on Claudication Distances And Lipid Profile In Patients With Occlusive Peripheral Arterial Disease. JK Practitioner. 2021;26(2):44-51.

246. McDermott MM, Bazzano L, Peterson CA, Sufit R, Ferrucci L, Domanchuk K, et al. Effect of Telmisartan on Walking Performance in Patients With Lower Extremity Peripheral Artery Disease: The TELEX Randomized Clinical Trial. JAMA. 2022;328(13):1315-25.

247. Haile ST, Joelsson-Alm E, Johansson UB, Loof H, Palmer-Kazen U, Gillgren P, et al. Effects of a person-centred, nurse-led follow-up programme on adherence to prescribed medication among patients surgically treated for intermittent claudication: randomized clinical trial. The British journal of surgery. 2022;109(9):846-56.

248. Dias-Santos EG, Farah BQ, Germano-Soares AH, Correia MdA, Souza AA, Hora JEJ, et al. Effects of Exercise Mode on Arterial Stiffness in Symptomatic Peripheral Artery Disease Patients: A Randomized Crossover Clinical Trial. Annals of vascular surgery. 2021;74(avs, 8703941):382-8.

249. Pasqualini L, Bagaglia F, Ministrini S, Frangione MR, Leli C, Siepi D, et al. Effects of structured home-based exercise training on circulating endothelial progenitor cells and endothelial function in patients with intermittent claudication. Vascular medicine (London, England). 2021;26(6):633-40.

250. Slysz JT, Tian L, Zhao L, Zhang D, McDermott MM. Effects of supervised exercise therapy on blood pressure and heart rate during exercise, and associations with improved walking performance in peripheral artery disease: Results of a randomized clinical trial. Journal of vascular surgery. 2021;74(5):1589-600.e4.

251. Suchkov IA, Mzhavanadze ND, Bogachev VY, Bokuchava M, Kuznetsov MR, Lukyanov YV, et al. Efficacy and safety of Actovegin in the treatment of intermittent claudication: results of an international, multicenter, placebo-controlled, randomized, phase IIIb clinical trial (APOLLO). International angiology : a journal of the International Union of Angiology. 2022;41(5):405-12.

252. Kalantzi K, Tentolouris N, Melidonis AJ, Papadaki S, Peroulis M, Amantos KA, et al. Efficacy and Safety of Adjunctive Cilostazol to Clopidogrel-Treated Diabetic Patients With Symptomatic Lower Extremity Artery Disease in the Prevention of Ischemic Vascular Events. Journal of the American Heart Association. 2020((Kalantzi, Papadaki, Peroulis, Dimitsikoglou, Doupis, Mandalaki, Triantafyllidis, Tsouka, Tselepis) Atherothrombosis Research Center Laboratory of Biochemistry Department of Chemistry University of Ioannina Greece(Tentolouris) 1st Department of Propaedeut):e018184.

253. Teichgraber U, Lehmann T, Aschenbach R, Scheinert D, Zeller T, Brechtel K, et al. Efficacy and safety of a novel paclitaxel-nano-coated balloo for femoropopliteal angioplasty: One-year results of the EffPac trial. EuroIntervention. 2020;15(18):E1633-E40.

254. Bonaca MP, Szarek M, Debus ES, Nehler MR, Patel MR, Anand SS, et al. Efficacy and safety of rivaroxaban versus placebo after lower extremity bypass surgery: A post hoc analysis of a "CASPAR like" outcome from VOYAGER PAD. Clinical cardiology. 2022;45(12):1143-6.

255. Kook H, Yu CW, Choi D, Ahn TH, Chang K, Cho J-M, et al. Efficacy and Safety of SID142 in Patients With Peripheral Arterial Disease: A Multicenter, Randomized, Double-Blind, Active-Controlled, Parallel-Group, Phase III Clinical Trial. Clinical therapeutics. 2022;44(4):508-28.

256. Gilchrist IC, Jr., Morrow DA, Creager MA, Olin JW, Scirica BM, Goodrich EL, et al. Efficacy and Safety of Vorapaxar by Intensity of Background Lipid-Lowering Therapy in Patients With Peripheral Artery Disease: Insights From the TRA2P-TIMI 50 Trial. Journal of the American Heart Association. 2021;10(20):e021412.

257. Goueffic Y, Torsello G, Zeller T, Esposito G, Vermassen F, Hausegger KA, et al. Efficacy of a Drug-Eluting Stent Versus Bare Metal Stents for Symptomatic Femoropopliteal Peripheral Artery Disease: Primary Results of the EMINENT Randomized Trial. Circulation. 2022;146(21):1564-76.

258. Caradu C, Stenson K, Houmaida H, Le Ny J, Lalys F, Ducasse E, et al. EndoNaut two-dimensional fusion imaging with a mobile C-arm for endovascular treatment of occlusive peripheral arterial disease. Journal of vascular surgery. 2022;75(2):651-9.e1.

259. Baig M, Kwok M, Aldairi A, Imran HM, Khan MS, Moustafa A, et al. Endovascular Intravascular Lithotripsy in the Treatment of Calcific Common Femoral Artery Disease: A Case Series With an 18-Month Follow-Up. Cardiovascular revascularization medicine : including molecular interventions. 2022;43(101238551):80-4.

260. Fakhry F, Rouwet EV, Spillenaar Bilgen R, Van Der Laan L, Wever JJ, Teijink JAW, et al. Endovascular Revascularization Plus Supervised Exercise Versus Supervised Exercise only for Intermittent Claudication: A Cost-Effectiveness Analysis. Circulation: Cardiovascular Interventions. 2021;14(7):E010703.

261. Peri-Okonny PA, Wang J, Gosch KL, Patel MR, Shishehbor MH, Safley DL, et al. Establishing Thresholds for Minimal Clinically Important Differences for the Peripheral Artery Disease Questionnaire. Circulation Cardiovascular quality and outcomes. 2021;14(5):e007232.

262. Bohme T, Noory E, Beschorner U, Jacques B, Burgelin K, Macharzina R, et al. Evaluation of Mortality Following Paclitaxel Drug-Coated Balloon Angioplasty of Femoropopliteal Lesions in the Real World. JACC Cardiovascular interventions. 2020;13(17):2052-61.

263. Monroe JC, Lin C, Perkins SM, Han Y, Motaganahalli RL, Roseguini BT. Heat therapy reduces blood pressure and circulating endothelin-1 levels, but does not improve walking performance or vascular function in patients with symptomatic peripheral artery disease. FASEB Journal. 2020;34(SUPPL 1).

264. Bohme T, Noory E, Brechtel K, Scheinert D, Bosiers M, Beschorner U, et al. Heparin-Bonded Stent-Graft for the Treatment of TASC II C and D Femoropopliteal Lesions: 36-Month Results of the Viabahn 25 cm Trial. Journal of endovascular therapy : an official journal of the International Society of Endovascular Specialists. 2021;28(2):222-8.

265. Kim T, Iannuzzi J, Schneider P, Ochoa Chaar CI. High Risk Characteristics for Clinical Failure After Isolated Femoropopliteal Peripheral Vascular Interventions. Journal of Vascular Surgery. 2021;74(4):e324.

266. Parodi JC, Fernandez S, Moscovich F, Pulmaria C. Hydration may reverse most symptoms of lower extremity intermittent claudication or rest pain. Journal of vascular surgery. 2020;72(4):1459-63.

267. Busch L, Stern M, Dannenberg L, Mourikis P, Grone M, Ozaslan G, et al. Impact of high on-treatment platelet reactivity after angioplasty in patients with peripheral arterial disease. Platelets. 2021;32(3):391-7.

268. Johnson CE, Manzur MF, Potter HA, Ortega AJ, Ding L, Rowe VL, et al. Impact of Perioperative Blood Transfusion in Anemic Patients Undergoing Infra Inguinal Bypass. Annals of vascular surgery. 2022;79(avs, 8703941):72-80.

269. Englund EK, Langham MC, Wehrli FW, Fanning MJ, Khan Z, Schmitz KH, et al. Impact of supervised exercise on skeletal muscle blood flow and vascular function measured with MRI in patients with peripheral artery disease. American journal of physiology Heart and circulatory physiology. 2022;323(3):H388-H96.

270. McCallum M, Cooper B, Matson S, Renwick B, Messeder SJ. Improving health behaviors in patients with peripheral arterial disease - A pilot study of supported self-management. Journal of vascular nursing : official publication of the Society for Peripheral Vascular Nursing. 2021;39(1):11-6.

271. Fukaya E, Welden S, Bukari A, Khan Z, Leeper N, Mohler E. Incentivizing physical activity through activity monitoring interventions in PAD - a pilot study. VASA Zeitschrift fur Gefasskrankheiten. 2021;50(2):145-50.

272. Mathlouthi A, Yei KS, Naazie I, Bertges DJ, Malas MB. Increased mortality with paclitaxel-eluting stents is driven by lesion length. Journal of vascular surgery. 2021;73(2):548-53.e2.

273. Kato T, Miura T, Yamamoto S, Miyashita Y, Hashizume N, Shoin K, et al. Intensive exercise therapy for restenosis after superficial femoral artery stenting: the REASON randomized clinical trial. Heart and vessels. 2022;37(9):1596-603.

274. Lawall H, Bramlage P, Gorath M, Wittig T. Intermittent Administration of Nitroglycerin Sublingual Powder Compared with Placebo in Outpatients with Peripheral Artery Disease: Results of a Randomised Proof of Concept Study. European journal of vascular and endovascular surgery : the official journal of the European Society for Vascular Surgery. 2021;61(3):457-65.

275. Tepe G, Brodmann M, Werner M, Bachinsky W, Holden A, Zeller T, et al. Intravascular Lithotripsy for Peripheral Artery Calcification: 30-Day Outcomes From the Randomized Disrupt PAD III Trial. JACC Cardiovascular interventions. 2021;14(12):1352-61.

276. Shammas NW, Purushottam B, Shammas WJ, Christensen L, Shammas G, Weakley D, et al. Jetstream Atherectomy Followed by Paclitaxel-Coated Balloons versus Balloon Angioplasty Followed by Paclitaxel-Coated Balloons: Twelve-Month Exploratory Results of the Prospective Randomized JET-RANGER Study. Vascular health and risk management. 2022;18(101273479):603-15.

277. Kokkinidis DG, Behan S, Jawaid O, Hossain P, Giannopoulos S, Singh GD, et al. Laser atherectomy and drug-coated balloons for the treatment of femoropopliteal in-stent restenosis: 2-Year outcomes. Catheterization and cardiovascular interventions : official journal of the Society for Cardiac Angiography & Interventions. 2020;95(3):439-46.

278. Schahab N, Prengel AK, Mahn T, Schaefer C, Fimmers R, Nickenig G, et al. Long-term clinical outcome and mortality risks after paclitaxel-coated balloon angioplasty in patients with peripheral artery disease: An observational clinical study. Health Science Reports. 2021;4(1):e236.

279. Teichgraber U, Lehmann T, Ingwersen M, Aschenbach R, Zeller T, Brechtel K, et al. Long-Term Effectiveness and Safety of Femoropopliteal Drug-Coated Balloon Angioplasty : 5-Year Results of the Randomized Controlled EffPac Trial. Cardiovascular and interventional radiology. 2022;45(12):1774-83.

280. Klaphake S, Fakhry F, Rouwet EV, van der Laan L, Wever JJ, Teijink JA, et al. Long-term Follow-up of a Randomized Clinical Trial Comparing Endovascular Revascularization Plus Supervised Exercise With Supervised Exercise Only for Intermittent Claudication. Annals of surgery. 2022;276(6):e1035-e43.

281. Gunnarsson T, Bergman S, Parsson H, Gottsater A, Lindgren H. Long Term Results of a Randomised Trial of Stenting of the Superficial Femoral Artery for Intermittent Claudication. European journal of vascular and endovascular surgery : the official journal of the European Society for Vascular Surgery. 2023(b8n, 9512728).

282. Kluckner M, Nierlich P, Hitzl W, Aschacher T, Gratl A, Wipper S, et al. Long-Term Results of Endovascular Treatment with Nitinol Stents for Femoropopliteal TASC II C and D Lesions. Medicina (Kaunas, Lithuania). 2022;58(9).

283. Ohki T, Kichikawa K, Yokoi H, Iida O, Yamaoka T, Maeda K, et al. Long-term results of the Japanese multicenter Viabahn trial of heparin bonded endovascular stent grafts for long and complex lesions in the superficial femoral artery. Journal of vascular surgery. 2021;74(6):1958-67.e2.

284. Eikelboom JW, Bosch J, Connolly SJ, Tyrwitt J, Fox KAA, Muehlhofer E, et al. Long-Term Treatment with the Combination of Rivaroxaban and Aspirin in Patients with Chronic Coronary or Peripheral Artery Disease: Outcomes During the Open Label Extension of the COMPASS trial. European heart journal Cardiovascular pharmacotherapy. 2022;8(8):786-95.

285. Krantz MJ, Debus SE, Hsia J, Patel MR, Anand SS, Nehler MR, et al. Low-dose rivaroxaban plus aspirin in older patients with peripheral artery disease undergoing acute limb revascularization: insights from the VOYAGER PAD trial. European heart journal. 2021;42(39):4040-8.

286. Hoel H, Pettersen EM, Hoiseth LO, Mathiesen I, Seternes A, Hisdal J. Lower Extremity Intermittent Negative Pressure for Intermittent Claudication. Follow-Up after 24 Weeks of Treatment. Annals of vascular surgery. 2021;75(avs, 8703941):253-8.

287. Nowakowski P, Uchto W, Hrycek E, Kachel M, Ludyga T, Polczyk F, et al. Microcrystalline paclitaxel-coated balloon for revascularization of femoropopliteal artery disease: Three-year outcomes of the randomized BIOPAC trial. Vascular medicine (London, England). 2021;26(4):401-8.

288. Shibutani S, Obara H, Matsubara K, Toya N, Isogai N, Ogino H, et al. Midterm Results of a Japanese Prospective Multicenter Registry of Heparin-Bonded Expanded Polytetrafluoroethylene Grafts for Above-the-Knee Femoropopliteal Bypass. Circulation journal : official journal of the Japanese Circulation Society. 2020;84(3):501-8.

289. Pekas EJ, Anderson CP, Park S-Y. Moderate dose of dietary nitrate improves skeletal muscle microvascular function in patients with peripheral artery disease. Microvascular research. 2023;146(mxw, 0165035):104469.

290. Eikelboom JW, Bhatt DL, Fox KAA, Bosch J, Connolly SJ, Anand SS, et al. Mortality Benefit of Rivaroxaban Plus Aspirin in Patients With Chronic Coronary or Peripheral Artery Disease. Journal of the American College of Cardiology. 2021;78(1):14-23.

291. Tan M, Urasawa K, Haraguchi T, Ando H, Tsubakimoto Y, Kamoi D, et al. Mortality risk after use of a paclitaxel-coated stent in femoropopliteal peripheral artery disease. Cardiovascular intervention and therapeutics. 2022;37(1):136-44.

292. Nordanstig J, James S, Andersson M, Andersson M, Danielsson P, Gillgren P, et al. Mortality with Paclitaxel-Coated Devices in Peripheral Artery Disease. The New England journal of medicine. 2020;383(26):2538-46.

293. Coca-Martinez M, Vitagliano M, Girsowicz EE, Obrand DI, Steinmetz OK, Bayne JP, et al. Multimodal Prehabilitation for Peripheral Arterial Disease: Results of an In-Trial Pilot Randomized Controlled Trial. Journal of Vascular Surgery. 2021;74(5):e426-e7.

294. Mietz S, Lehmann T, Teichgraber U. Multivariable Regression Analysis of Clinical Data from the Randomized-Controlled EffPac Trial: Efficacy of Femoropopliteal Drug-Coated Balloon Angioplasty. Cardiovascular and interventional radiology. 2020;43(6):840-9.

295. Tsai S, Liu Y, Alaiti MA, Gutierrez JA, Brilakis ES, Banerjee S. No benefit of vorapaxar on walking performance in patients with intermittent claudication. Vascular medicine (London, England). 2022;27(1):33-8.

296. Soga Y, Fujihara M, Yamamoto Y, Nakamura S, Iida O, Kawasaki D, et al. One-year results for Japanese patients in RANGER II SFA. Heart and vessels. 2022;37(4):568-73.

297. Brodmann M, Wissgott C, Brechtel K, Nikol S, Zeller T, Lichtenberg M, et al. Optimized drug-coated balloon angioplasty of the superficial femoral and proximal popliteal arteries using the Tack Endovascular System: TOBA III 12-month results. Journal of vascular surgery. 2020;72(5):1636-47.e1.

298. Zeller T, Giannopoulos S, Brodmann M, Werner M, Andrassy M, Schmidt A, et al. Orbital Atherectomy Prior to Drug-Coated Balloon Angioplasty in Calcified Infrapopliteal Lesions: A Randomized, Multicenter Pilot Study. Journal of endovascular therapy : an official journal of the International Society of Endovascular Specialists. 2022;29(6):874-84.

299. Liao C-J, Song S-H, Li T, Zhang YZ, Wang d. Orchid drug-coated balloon versus standard percutaneous transluminal angioplasty for the treatment of femoropopliteal artery disease: 12-month result of the randomized controlled trial. Vascular. 2022;30(3):448-54.

300. Kozuki A, Takahara M, Shimizu M, Kijima Y, Nagoshi R, Fujiwara R, et al. Outcomes of Dissection Angles as Predictor of Restenosis after Drug-Coated Balloon Treatment. Journal of atherosclerosis and thrombosis. 2020((Kozuki, Shimizu, Kijima, Nagoshi, Fujiwara, Shibata, Suzuki, Soga, Miyata, Sakamoto, Seo, Asada, Isawa, Higuchi, Shite) Osaka Saiseikai Nakatsu Hospital, Division of Cardiology(Takahara) Department of Diabetes Care Medicine, Osaka University Graduate Sch).

301. Kim TI, Kiwan G, Mohamedali A, Zhang Y, Mena-Hurtado C, Mojibian H, et al. Outcomes of treatment with paclitaxel-coated devices for peripheral arterial disease. Journal of vascular surgery. 2021;73(3):911-7.

302. Gahide G, Phaneuf SC, Cossette M, Banine A, Budimir M, Maghsoudloo K, et al. Paclitaxel and mortality in patients with claudication and de novo femoropopliteal lesions: a historical cohort study. CVIR endovascular. 2021;4(1):65.

303. Tepe G, Zeller T, Moscovic M, Corpataux J-M, Christensen JK, Keirse K, et al. Paclitaxel-Coated Balloon Angioplasty for the Treatment of Infrainguinal Arteries: 24-Month Outcomes in the Full Cohort of BIOLUX P-III Global Registry. Cardiovascular and interventional radiology. 2021;44(2):207-17.

304. Ribeiro TF, Ferreira RS, Correia R, Amaral C, Goncalves FB, Ferreira ME. Paclitaxel in real-life data is not associated with reduced survival but has limited benefit in preventing amputation. International angiology : a journal of the International Union of Angiology. 2022;41(3):205-11.

305. Zielinski LP, Chowdhury MM, Coughlin PA. Patient and Institutional Costs of Failure of Angioplasty of the Superficial Femoral Artery. Annals of vascular surgery. 2021;72(avs, 8703941):218-26.

306. Fox KAA, Aboyans V, Debus ES, Zeymer U, Cowie MR, Patel M, et al. Patients selected for dual pathway inhibition in clinical practice have similar characteristics and outcomes to those included in the COMPASS randomized trial: The XATOA Registry. European heart journal Cardiovascular pharmacotherapy. 2022;8(8):825-36.

307. Bohme T, Noory E, Beschorner U, Lerke F, Schmidt A, Scheinert D, et al. Photoablative atherectomy followed by a paclitaxel-coated balloon to inhibit restenosis in instent femoro-popliteal obstructions (PHOTOPAC): A randomized multicentre pilot study. Vasa - European Journal of Vascular Medicine. 2021;50(5):387-93.

308. Pan T, Jiang X, Liu H, Liu Y, Fu W, Dong Z. Prediction of 2-Year Major Adverse Limb Event-Free Survival After Percutaneous Transluminal Angioplasty and Stenting for Lower Limb Atherosclerosis Obliterans: A Machine Learning-Based Study. Frontiers in Cardiovascular Medicine. 2022;9((Pan, Jiang, Liu, Liu, Fu, Dong) Department of Vascular Surgery, Institute of Vascular Surgery, Zhongshan Hospital, Fudan University, Shanghai, China(Pan, Jiang, Liu, Liu, Fu, Dong) National Clinical Research Center for Interventional Medicine, Shanghai):783336.

309. Moll MA, Zwerger D, Grassl KJ, Westreicher W, Neururer SB, Moll CW, et al. Prevalence of VOYAGER PAD trial exclusion criteria in unselected patients undergoing lower limb revascularization. International angiology : a journal of the International Union of Angiology. 2022;41(1):56-62.

310. Berkowitz SD, Bauersachs RM, Szarek M, Nehler MR, Debus ES, Patel MR, et al. Prevention of arterial and venous vascular events in symptomatic peripheral arterial disease patients after lower extremityrevascularization in the VOYAGER PAD trial: Dual anticoagulant/antiplatelet regimen vs antiplatelet therapy alone. Research and Practice in Thrombosis and Haemostasis. 2021;5(SUPPL 2).

311. Brouillet J, Deloose K, Goueffic Y, Poirier M, Midy D, Caradu C, et al. Primary stenting for TASC C and D femoropopliteal lesions: one-year results from a multicentric trial on 203 patients. The Journal of cardiovascular surgery. 2018;59(3):392-404.

312. Sharma S, Pandey NN, Sinha M, Kumar S, Jagia P, Gulati GS, et al. Randomized, Double-Blind, Placebo-Controlled Trial to Evaluate Safety and Therapeutic Efficacy of Angiogenesis Induced by Intraarterial Autologous Bone Marrow-Derived Stem Cells in Patients with Severe Peripheral Arterial Disease. Journal of vascular and interventional radiology : JVIR. 2021;32(2):157-63.

313. Shishehbor MH, Zeller T, Werner M, Brodmann M, Parise H, Holden A, et al. Randomized Trial of Chocolate Touch Compared With Lutonix Drug-Coated Balloon in Femoropopliteal Lesions (Chocolate Touch Study). Circulation. 2022;145(22):1645-54.

314. Jiang Y, Fan J, Li Y, Wu G, Wang Y, Yang J, et al. Rapid reduction in plaque inflammation by sonodynamic therapy inpatients with symptomatic femoropopliteal peripheral artery disease:A randomized controlled trial. International journal of cardiology. 2021;325(gqw, 8200291):132-9.

315. Ye W, Zhang X, Dai X, Huang X, Liu Z, Jiang M, et al. ReewarmTM PTX drug-coated balloon in the treatment of femoropopliteal artery disease: A multi-center, randomized controlled trial in China. International journal of cardiology. 2020((Ye, Liu) Department of Vascular Surgery, Chinese Academy of Medical Science, Peking Union Medical College Hospital, Beijing, China(Zhang) Department of Vascular Surgery, Peking University People's Hospital, Beijing, China(Dai) Department of Vascular Surg).

316. Kaplovitch E, Eikelboom JW, Dyal L, Aboyans V, Abola MT, Verhamme P, et al. Rivaroxaban and Aspirin in Patients With Symptomatic Lower Extremity Peripheral Artery Disease: A Subanalysis of the COMPASS Randomized Clinical Trial. JAMA cardiology. 2020((Kaplovitch, Eikelboom, Dyal, Bangdiwala, Yusuf, Anand) Population Health Research Institute, McMaster University, Hamilton Health Sciences, Hamilton, Ontario, Canada(Kaplovitch) Department of Medicine, University of Toronto, Toronto, ON, Canada(Eikelboom).

317. Guzik TJ, Ramasundarahettige C, Pogosova N, Lopez-Jaramillo P, Dyal L, Berkowitz SD, et al. Rivaroxaban Plus Aspirin in Obese and Overweight Patients With Vascular Disease in the COMPASS Trial. Journal of the American College of Cardiology. 2021;77(5):511-25.

318. Imran H, Hyder O, Soukas P. Role of adjunctive drug coated balloon therapy in endovascular treatment of common femoral artery disease. European Heart Journal. 2017;38(Supplement 1):1086.

319. Hess CN, Patel MR, Bauersachs RM, Anand SS, Debus ES, Nehler MR, et al. Safety and Effectiveness of Paclitaxel Drug-Coated Devices in Peripheral Artery Revascularization: Insights From VOYAGER PAD. Journal of the American College of Cardiology. 2021;78(18):1768-78.

320. Ali AAS, Elhady AAEA, Elaskry N, Elnahhas NG. SHOCK WAVE VERSUS DYNAMIC TRAINING FOR INTERMITTENT CLAUDICATION IN DIABETIC PATIENTS TYPE II. Journal of Pharmaceutical Negative Results. 2022;13((Ali, Elhady, Elnahhas) Department of Physical Therapy for Cardiovascular/Respiratory Disorders and Geriatrics, Faculty of Physical Therapy, Cairo University, Egypt(Elaskry) Department of Vascular Surgery, Faculty of Medicine, Alexandria University, Egypt):5234-41.

321. Bosiers M, Deloose K, Callaert J, Verbist J, Hendriks J, Lauwers P, et al. Stent-grafts are the best way to treat complex in-stent restenosis lesions in the superficial femoral artery: 24-month results from a multicenter randomized trial. The Journal of cardiovascular surgery. 2020;61(5):617-25.

322. Paldan K, Ullrich G, Rammos C, Steinmetz M, Janosi A, Moebus S, et al. Supervised exercise therapy using mobile health technology in patients with peripheral arterial disease-a pilot randomized controlled trial. Vasa - European Journal of Vascular Medicine. 2021;50(SUPPL 106):29.

323. Slysz JT, Rejeski WJ, Treat-Jacobson D, Bazzano LA, Forman DE, Manini TM, et al. Sustained physical activity in peripheral artery disease: Associations with disease severity, functional performance, health-related quality of life, and subsequent serious adverse events in the LITE randomized clinical trial. Vascular medicine (London, England). 2021;26(5):497-506.

324. Nasr B, Della Schiava N, Thaveau F, Rosset E, Favre J-P, Salomon du Mont L, et al. The Common Femoral Artery Bifurcation Lesions: Clinical Outcome of Simple Versus Complex Stenting Techniques - An Analysis Based on the TECCO Trial. Annals of vascular surgery. 2020;64(avs, 8703941):2-10.

325. Nastasi DR, Moxon JV, Norman R, Trollope AF, Rowbotham S, Quigley F, et al. The cost effectiveness of intensive low density lipoprotein-cholesterol lowering in people with peripheral artery disease. Journal of vascular surgery. 2020((Nastasi) Queensland Research Centre for Peripheral Vascular Disease, College of Medicine and Dentistry, James Cook University, Townsville, QLD, Australia(Moxon, Trollope) Queensland Research Centre for Peripheral Vascular Disease, College of Medicine and).

326. Pettersen EM, Hoel H, Torp H, Hisdal J, Seternes A. The Effect of 12-Week Treatment with Intermittent Negative Pressure on Blood Flow Velocity and Flowmotion, Measured with a Novel Doppler Device (Earlybird). Secondary Outcomes from a Randomized Sham-Controlled Trial in Patients with Peripheral Arterial Di. Annals of vascular surgery. 2022;86(avs, 8703941):144-57.

327. Kapusta J, Irzmanski R. The Impact of Controlled Physical Training with Hydrotherapy on Changes in Swelling and Claudication Distance in Patients with Atherosclerotic Ischemia of the Lower Limbs. International journal of environmental research and public health. 2022;19(23).

328. Allan RB, Puckridge PJ, Spark JI, Delaney CL. The Impact of Intravascular Ultrasound on Femoropopliteal Artery Endovascular Interventions: A Randomized Controlled Trial. JACC Cardiovascular interventions. 2022;15(5):536-46.

329. Schroe H, Sachar R, Keirse K, Soga Y, Brodmann M, Rao V, et al. The RANGER II superficial femoral artery trial: 1-year results of the long lesion cohort. Vascular medicine (London, England). 2022;27(5):457-65.

330. Tan M, Takahara M, Soga Y, Mori S, Tsuchiya T, Mazaki T, et al. Three-Year Clinical Outcomes Following Implantation of LifeStent Self-Expanding Nitinol Stents in Patients With Femoropopliteal Artery Lesions. Angiology. 2022;73(3):244-51.

331. Giannopoulos S, Mustapha J, Gray WA, Ansel G, Adams G, Secemsky EA, et al. Three-Year Outcomes From the LIBERTY 360 Study of Endovascular Interventions for Peripheral Artery Disease Stratified by Rutherford Category. Journal of Endovascular Therapy. 2021;28(2):262-74.

332. Soga Y, Iida O, Urasawa K, Saito S, Jaff MR, Wang H, et al. Three-Year Results of the IN.PACT SFA Japan Trial Comparing Drug-Coated Balloons With Percutaneous Transluminal Angioplasty. Journal of endovascular therapy : an official journal of the International Society of Endovascular Specialists. 2020;27(6):946-55.

333. Salisbury DL, Swanson K, Brown RJ, Treat-Jacobson D. Total body recumbent stepping vs treadmill walking in supervised exercise therapy: A pilot study. Vascular medicine (London, England). 2022;27(2):150-7.

334. Szarek M, Hess C, Patel MR, Jones WS, Berger JS, Baumgartner I, et al. Total Cardiovascular and Limb Events and the Impact of Polyvascular Disease in Chronic Symptomatic Peripheral Artery Disease. Journal of the American Heart Association. 2022;11(11):e025504.

335. Bauersachs RM, Szarek M, Brodmann M, Gudz I, Debus ES, Nehler MR, et al. Total Ischemic Event Reduction With Rivaroxaban After Peripheral Arterial Revascularization in the VOYAGER PAD Trial. Journal of the American College of Cardiology. 2021;78(4):317-26.

336. Saratzis A, Salem M, Sabbagh C, Abisi S, Huasen B, Egun A, et al. Treatment of Aortoiliac Occlusive Disease With the Covered Endovascular Reconstruction of the Aortic Bifurcation (CERAB) Technique: Results of a UK Multicenter Study. Journal of endovascular therapy : an official journal of the International Society of Endovascular Specialists. 2021;28(5):737-45.

337. Sullivan TM, Zeller T, Nakamura M, Gaines PA. Treatment of Femoropopliteal Lesions With the BioMimics 3D Vascular Stent System: Two-Year Results From the MIMICS-2 Trial. Journal of endovascular therapy : an official journal of the International Society of Endovascular Specialists. 2021;28(2):236-45.

338. Nasr B, Gouailler F, Marret O, Guillou M, Chaillou P, Guyomarc'h B, et al. Treatment of Long Femoropopliteal Lesions With Self-Expanding Interwoven Nitinol Stent: 24 Month Outcomes of the STELLA-SUPERA Trial. Journal of endovascular therapy : an official journal of the International Society of Endovascular Specialists. 2023;30(1):98-105.

339. Muller-Hulsbeck S, Benko A, Soga Y, Fujihara M, Iida O, Babaev A, et al. Two-Year Efficacy and Safety Results from the IMPERIAL Randomized Study of the Eluvia Polymer-Coated Drug-Eluting Stent and the Zilver PTX Polymer-free Drug-Coated Stent. Cardiovascular and interventional radiology. 2021;44(3):368-75.

340. Shammas NW, Shammas GA, Karia R, Khalafallah R, Jones-Miller S, Shammas AN. Two-Year Outcomes of Endovascular Interventions of the Common Femoral Artery: A Retrospective Analysis From Two Medical Centers. Cardiovascular revascularization medicine : including molecular interventions. 2021;24(101238551):72-6.

341. Fukuda K, Okazaki S, Shiozaki M, Okai I, Nishino A, Tamura H, et al. Ultrasound-guided puncture reduces bleeding-associated complications, regardless of calcified plaque, after endovascular treatment of femoropopliteal lesions, especially using the antegrade procedure: A single-center study. PloS one. 2021;16(3):e0248416.

342. Landry GJ, Louie D, Giraud D, Ammi AY, Kaul S. Ultrasound therapy for treatment of lower extremity intermittent claudication. American journal of surgery. 2021;221(6):1271-5.

343. Hogan SE, Nehler M, Anand S, Patel MR, Debus ES, Jackson MT, et al. WALKING IMPAIRMENT IN PATIENTS WITH SYMPTOMATIC PERIPHERAL ARTERY DISEASE AFTER LOWER EXTREMITY REVASCULARIZATION. Journal of the American College of Cardiology. 2022;79(9 Supplement):1774.

344. Manfredini F, Traina L, Ficarra V, Gandolfi G, Argentoni A, Straudi S, et al. A "test in-train out" program versus a "go home and walk" intervention for home-based exercise therapy in patients with peripheral artery disease: A randomized controlled trial. Scandinavian journal of medicine & science in sports. 2024;34(2):e14584.

345. Fang Y, Kan Y, Guo W, Hong B, Shu C, Wang F, et al. A New Drug-Coated Balloon for the Treatment of Superficial Femoropopliteal Artery Disease: 12-Month Results from the IN-DEPT SFA Trial. Journal of vascular and interventional radiology : JVIR. 2024;35(2):251-8.

346. Iida O, Soga Y, Saito S, Mano T, Hayakawa N, Ichihashi S, et al. A Novel Sirolimus-Coated Balloon for the Treatment of Femoropopliteal Lesions: The SELUTION SFA Japan Trial. JACC Cardiovascular interventions. 2024;17(13):1547-56.

347. Bertges DJ, Eldrup-Jorgensen J, Chaer RA, Stoner MC, Marone LK, Giles KA, et al. A registry-based study of paclitaxel drug-coated balloon angioplasty for the treatment of in-stent restenosis of the femoral-popliteal artery. Journal of vascular surgery. 2024;79(5):1142-50.e2.

348. Noory E, Bohme T, Steinhauser Y, Salm J, Beschorner U, de Forest A, et al. Acute and Mid-Term Results of Atherectomy in Femoropopliteal Lesions. Journal of Endovascular Therapy. 2024((Noory, Bohme, Steinhauser, Salm, Beschorner, de Forest, Bollenbacher, Westermann, Zeller) Department of Cardiology and Angiology, Medical Center, University of Freiburg, Bad Krozingen, Germany).

349. Herrero-Alonso C, Lopez-Lifante V-M, Costa-Garrido A, Pera G, Alzamora M, Fores R, et al. Adherence to Supervised and Unsupervised Exercise Programmes in Ageing Population with Intermittent Claudication: A Randomized Controlled Trial. Journal of clinical medicine. 2024;13(13).

350. Rrapo-Kaso E, Loffler AI, Petroni GR, Meyer CH, Walker M, Kay JR, et al. Alirocumab and plaque volume, calf muscle blood flow, and walking performance in peripheral artery disease: A randomized clinical trial. Vascular medicine (London, England). 2023;28(4):282-9.

351. Liu Y, Xue J, Jiang J. Application of machine learning algorithms in electronic medical records to predict amputation-free survival after first revascularization in patients with peripheral artery disease. International journal of cardiology. 2023;383(gqw, 8200291):175-84.

352. McBane RD, 2nd, Murphree DH, Liedl D, Lopez-Jimenez F, Attia IZ, Arruda-Olson AM, et al. Artificial Intelligence of Arterial Doppler Waveforms to Predict Major Adverse Outcomes Among Patients Evaluated for Peripheral Artery Disease. Journal of the American Heart Association. 2024;13(3):e031880.

353. Bentzen A, Nisgaard LB, Mikkelsen RBL, Hogh A, Mechlenburg I, Jorgensen SL. Blood flow restricted walking in patients suffering from intermittent claudication: a case series feasibility and safety study. Annals of Medicine and Surgery. 2023;85(5):1430-5.

354. Vrsalovic M, Heimark S, Soraas CL, Mehlum MH, Kjeldsen SE, Mancia G, et al. Cardiovascular Outcomes in Hypertension-Treated Patients With Peripheral Artery Disease: The VALUE Trial. Hypertension (Dallas, Tex : 1979). 2024;81(7):1628-36.

355. Bohme T, Zeller T, Shishehbor MH, Werner M, Brodmann M, Parise H, et al. Chocolate Touch Versus Lutonix Drug-Coated Balloon for Femoropopliteal Lesions in Diabetes: The Chocolate Touch Study. Journal of Endovascular Therapy. 2023((Bohme, Zeller, Beschorner) Department of Cardiology and Angiology, Medical Center, University of Freiburg, Bad Krozingen, Germany(Shishehbor) University Hospitals Harrington Heart & Vascular Institute, Cleveland, OH, United States(Werner) Department of A).

356. Ulfsdottir H, Back M, Cider A, Jivegard L, Sandberg A, Nordanstig J, et al. Cost-Effectiveness of Exercise Therapy in Patients with Intermittent Claudication-A Comparison of Supervised Exercise, Home-Based Structured Exercise, and Walk Advice from the SUNFIT Trial. Journal of clinical medicine. 2023;12(16).

357. Lyden SP, Soukas PA, De A, Tedder B, Bowman J, Mustapha JA, et al. DETOUR2 trial outcomes demonstrate clinical utility of percutaneous transmural bypass for the treatment of long segment, complex femoropopliteal disease. Journal of vascular surgery. 2024;79(6):1420-7.e2.

358. Xiong H, Wang D, Song P, Quan X, Zhang M, Huang S, et al. Development and validation of a major adverse limb events prediction model for peripheral arterial disease with frailty. Journal of Vascular Surgery. 2024;80(1):177-87.e2.

359. Kodama K, Soga Y, Tomoi Y, Sakai N, Imada K, Katsuki T, et al. Difference in one-year late lumen loss between high- and low-dose paclitaxel-coated balloons for femoropopliteal disease. Heart and vessels. 2024;39(7):582-8.

360. Danisan G, Taydas O, Ozdemir M, Ates OF, Kupeli A, Oguslu U, et al. Dynamic thiol-disulphide homeostasis as a biomarker for predicting the development of contrast medium-associated acute kidney injury in the endovascular treatment of peripheral arterial disease: should intravenous N-acetylcysteine be given before the proc. Clinical radiology. 2023;78(6):466-72.

361. Silva I, Moreira CS, Pedras S, Oliveira R, Veiga C, Moreira L, et al. Effect of a monitored home-based exercise program combined with a behavior change intervention and a smartphone app on walking distances and quality of life in adults with peripheral arterial disease: the WalkingPad randomized clinical trial. Frontiers in cardiovascular medicine. 2023;10(101653388):1272897.

362. Garcia EL, Pereira AH, Menezes MG, Pereira AA, Stein R, Franzoni LT, et al. Effects of aerobic and combined training on pain-free walking distance and health-related quality of life in patients with peripheral artery disease: a randomized clinical trial. Jornal vascular brasileiro. 2023;22(101262256):e20230024.

363. Gardner AW, Montgomery PS, Wang M, Liang M. Effects of Long-Term Home Exercise in Participants With Peripheral Artery Disease. Journal of the American Heart Association. 2023;12(21):e029755.

364. Perks J, McBride P, Rayt H, Payne T, Edwardson C, Rowlands AV, et al. Efficacy of a personalised activity plan for BREAKing UP sitting time in patients with intermittent claudication (the BREAK UP study). Diabetes Research and Clinical Practice. 2023;204((Perks, Rayt, Payne, Messeder, Sayers) Department of Cardiovascular Sciences, University of Leicester, On-Call Suite OC9, Glenfield General Hospital, Groby Road, Leicester LE3 9QP, United Kingdom(Mcbride, Edwardson, Rowlands, Yates) Leicester Diabetes Cen):110925.

365. Arora E, Maiya GA, Devasia T, Bhat R, Kamath G. Efficacy of comprehensive structured exercise program on claudication pain and quality of life in type 2 diabetes mellitus with peripheral arterial disease. Journal of Diabetes and Metabolic Disorders. 2024;23(1):1305-13.

366. Cai P, Pymer S, Ibeggazene S, Raza A, Hitchman L, Chetter I, et al. Extracorporeal Shockwave for Intermittent Claudication and Quality of Life: A Randomized Clinical Trial. JAMA surgery. 2024;159(6):625-32.

367. Waddell A, Denton F, Powell R, Broom DR, Birkett ST, McGregor G, et al. Home-based Circuit Training and Community Walking for Intermittent Claudication. Annals of vascular surgery. 2024;105(avs, 8703941):38-47.

368. Cerrud-Rodriguez RC, Romain G, Hussain Y, Cleman J, Callegari S, Scierka L, et al. Impact of early intervention on health status outcomes in peripheral artery disease patients with chronic total occlusion lesions using the PORTRAIT registry. Journal of Vascular Surgery. 2024((Cerrud-Rodriguez, Romain, Hussain, Cleman, Callegari, Scierka, Smolderen, Mena-Hurtado) Vascular Medicine Outcomes (VAMOS) Program, Section of Cardiovascular Medicine, Department of Internal Medicine, Yale University School of Medicine, New Haven, CT, Un).

369. Sandberg A, Back M, Cider A, Jivegard L, Sigvant B, Nordanstig J. Impact of walk advice alone or in combination with supervised or home-based structured exercise on patient-reported physical function and generic and disease-specific health related quality of life in patients with intermittent claudication, a secondary a. Health and quality of life outcomes. 2023;21(1):114.

370. Shammas NW, Shammas G, Christensen L, Jones-Miller S. Jetstream Atherectomy with Paclitaxel-Coated Balloons: Two-Year Outcome of the Prospective Randomized JET-RANGER Study. Vascular health and risk management. 2023;19(101273479):133-7.

371. Bonaca MP, Bhatt DL, Simon T, Fox KM, Mehta S, Harrington RA, et al. Limb Outcomes With Ticagrelor Plus Aspirin in Patients With Diabetes Mellitus and Atherosclerosis. Journal of the American College of Cardiology. 2024;83(17):1627-36.

372. Caruso P, Maiorino MI, Longo M, Porcellini C, Matrone R, Digitale Selvaggio L, et al. Liraglutide for Lower Limb Perfusion in People With Type 2 Diabetes and Peripheral Artery Disease: The STARDUST Randomized Clinical Trial. JAMA network open. 2024;7(3):e241545.

373. Katsogridakis E, Saha P, Diamantopoulos A, Saratzis N, Davies R, Zayed H, et al. Long-Term Effects of Acute Kidney Injury Following Endovascular Femoropopliteal Intervention: Insights From a Multicenter Trial. Journal of endovascular therapy : an official journal of the International Society of Endovascular Specialists. 2024;31(4):634-42.

374. Nakama T, Takahara M, Iwata Y, Suzuki K, Tobita K, Hayakawa N, et al. Low-Dose vs High-Dose Drug-Coated Balloon for Symptomatic Femoropopliteal Artery Disease: The PROSPECT MONSTER Study Outcomes. JACC Cardiovascular interventions. 2023;16(21):2655-65.

375. Parkington T, Broom D, Maden-Wilkinson T, Nawaz S, Klonizakis M. Low-intensity resistance exercise with blood flow restriction for patients with claudication: A randomized controlled feasibility trial. Vascular medicine (London, England). 2023;28(6):554-63.

376. Wargny M, Leux C, Chatellier G, Coudol S, Gourraud P-A, Goueffic Y. Mortality in a Nationwide Practice-Based Cohort Receiving Paclitaxel-Coated Devices for Lower Limb Peripheral Artery Disease. Journal of the American College of Cardiology. 2024;83(13):1207-21.

377. Coca-Martinez M, Girsowicz E, Doonan RJ, Obrand DI, Bayne JP, Steinmetz OK, et al. Multimodal Prehabilitation for Peripheral Arterial Disease Patients with Intermittent Claudication-A Pilot Randomized Controlled Trial. Annals of vascular surgery. 2023(avs, 8703941).

378. Fransson T, Sturedahl AD, Resch T, Bjorn E, Gottsater A. Nationwide Study of the Outcome of Treatment of Lower Extremity Atherosclerotic Lesions With Endovascular Surgery With or Without Drug Eluting Methods in Patients With Diabetes. Journal of Endovascular Therapy. 2024((Fransson, Resch, Gottsater) Department of Clinical Sciences, Lund University, Malmo, Sweden(Fransson) Vascular Center, Department of Thoracic and Vascular Surgery, Skane University Hospital, Malmo, Sweden(Sturedahl, Bjorn) National Diabetes Register, Dep).

379. Burgess L, Babber A, Shalhoub J, Smith S, de la Rosa CN, Fiorentino F, et al. Neuromuscular Electrical Stimulation for Intermittent Claudication (NESIC): multicentre, randomized controlled trial. The British journal of surgery. 2023;110(12):1785-92.

380. McDermott MM, Martens CR, Domanchuk KJ, Zhang D, Peek CB, Criqui MH, et al. Nicotinamide riboside for peripheral artery disease: the NICE randomized clinical trial. Nature communications. 2024;15(1):5046.

381. Aragao JA, Neves OMG, Aragao ICS, Aragao FMS, Lourenco BC, Porto LC, et al. Occurrence of depression and assessment of functional capacity in patients with vascular diseases admitted to a Vascular Surgery Service. Jornal Vascular Brasileiro. 2023;22((Aragao) Universidade Federal de Sergipe - UFS, SE, Aracaju, Brazil(Neves) Fundacao Beneficencia Hospital Cirurgia - FBHC, Servico de Cirurgia Vascular, SE, Aracaju, Brazil(Aragao) Hospital Municipal Munir Rafful - HMMR, RJ, Volta Redonda, Brazil(Aragao)):e20230082.

382. Ducasse E, Sapoval M, Brunet J, Commeau P, Goueffic Y, Sabatier J, et al. Outcomes and Comparative Analysis of the Initial Results of Standard Balloon Angioplasty Versus Drug-Coated Balloons Alone Versus in Association With Laser-Excimer Atherectomy in the Treatment of Femoropopliteal Artery In-Stent Restenosis (INTACT). Journal of Endovascular Therapy. 2024((Ducasse, Caradu) Department of Vascular Surgery, Centre Hospitalier Universitaire de Bordeaux, Bordeaux, France(Sapoval) Hopital Europeen Georges-Pompidou, Assistance Publique-Hopitaux de Paris, Paris, France(Brunet) Clinique Rhone Durance, Avignon, Fran).

383. Haile ST, Johansson U-B, Loof H, Linne A, Joelsson-Alm E. Patient Related Outcomes After Receiving a Person Centred Nurse Led Follow Up Programme Among Patients Undergoing Revascularisation for Intermittent Claudication: A Secondary Analysis of a Randomised Clinical Trial. European journal of vascular and endovascular surgery : the official journal of the European Society for Vascular Surgery. 2023;66(3):371-9.

384. Cleary CM, Adajian A, Gifford ED, Healy L, Li YH, Dawiczyk S, et al. Patient Reported Barriers for Participation in Supervised Exercise Therapy for Symptomatic Peripheral Artery Disease. Annals of Vascular Surgery. 2024;106((Cleary, Healy) University of Connecticut School of Medicine, Farmington, CT, United States(Adajian, Gifford, Dawiczyk, Bozeman, Guerin, Farrell, Shah) Hartford Hospital Division of Vascular and Endovascular Surgery, Hartford, CT, United States(Li) Hartfo):124-31.

385. Rahman H, Leutzinger T, Hassan M, Schieber M, Koutakis P, Fuglestad MA, et al. Peripheral artery disease causes consistent gait irregularities regardless of the location of leg claudication pain. Annals of physical and rehabilitation medicine. 2024;67(3):101793.

386. Donas KP, Psyllas A, Pitoulias AG, Kazemtash M, Dahi F, Abu Bakr N, et al. Periprocedural Outcomes of Rotational Atherectomy-Assisted Balloon Angioplasty in Isolated Atherosclerotic Popliteal Artery Lesions: The ISO-POP Trial. Journal of clinical medicine. 2023;12(8).

387. Gray WA, Soga Y, Fujihara M, Iida O, Babaev A, Kawasaki D, et al. Polymer-based drug-eluting stent treatment extends the time to reintervention for patients with symptomatic femoropopliteal artery disease: clinical evidence and potential economic value. Journal of comparative effectiveness research. 2024;13(6):e240025.

388. Bohr NL, Brown G, Rakel B, Babrowski T, Dorsey C, Skelly C. Predictive Modeling for One-Year Lower Extremity Endovascular Revascularization Failure in Black Persons. Journal of Surgical Research. 2024;300((Bohr) Department of Nursing Research, UChicago Medicine, Chicago, Illinois, United States(Bohr, Babrowski, Dorsey, Skelly) Department of Surgery, Section of Vascular and Endovascular Surgery, University of Chicago, Chicago, Illinois, United States(Brown)):117-26.

389. Scierka LE, Peri-Okonny PA, Romain G, Cleman J, Spertus JA, Fitridge R, et al. Psychosocial and socioeconomic factors are most predictive of health status in patients with claudication. Journal of Vascular Surgery. 2024;79(6):1473-82.e5.

390. Yuan Z, Levitan B, Deng H, Szarek M, Bauersachs RM, Berkowitz SD, et al. Quantitative Benefit-Risk Evaluation of Rivaroxaban in Patients After Peripheral Arterial Revascularization: The VOYAGER PAD Trial. Journal of the American Heart Association. 2024;13(8):e032782.

391. Wittig T, Schmidt A, Fus T, Thieme M, Maiwald L, Dusing S, et al. Randomized Trial Comparing a Stent-Avoiding With a Stent-Preferred Strategy in Complex Femoropopliteal Lesions. JACC Cardiovascular interventions. 2024;17(9):1134-44.

392. Amanvermez Senarslan D, Yildirim F, Bayram B, Kurdal AT, Tetik O. Results of endovascular treatments of Trans-Atlantic Inter-Society Consensus C or D aortoiliac occlusive disease involving the aortic bifurcation. SAGE open medicine. 2023;11(101624744):20503121231179836.

393. Govsyeyev N, Nehler M, Conte MS, Debus S, Chung J, Dorigo W, et al. Rivaroxaban in patients with symptomatic peripheral artery disease after lower extremity bypass surgery with venous and prosthetic conduits. Journal of vascular surgery. 2023;77(4):1107-18.e2.

394. Schofthaler C, Troisi N, Torsello G, Jehn A, Lichtenberg M, Karcher JC, et al. Safety and effectiveness of the phoenix atherectomy device for endovascular treatment of common femoral and popliteal arteries: Results of the EN-MOBILE trial. Vascular medicine (London, England). 2024(c19, 9610930):1358863X241231943.

395. Nagpal S, Altin SE, McGinigle K, Mangalmurti SS, Adams G, Shammas NW, et al. Sex-specific analysis of intravascular lithotripsy for peripheral artery disease from the Disrupt PAD III observational study. Journal of Vascular Surgery. 2024;79(2):358-65.

396. Franzese M, Pucciarelli A, Spione F, Salemme L, Popusoi G, Ferrone M, et al. Sirolimus-Coated Balloon in Femoropopliteal Steno-Occlusive Disease: Efficacy, Safety, and 1-Year Outcomes. An All-Comers Registry. Journal of endovascular therapy : an official journal of the International Society of Endovascular Specialists. 2023(dmk, 100896915):15266028231217657.

397. Kronlage M, Bertele M, Linden F, Frey N, Erbel C. Stand-Alone Rotational Atherectomy Versus Combination With Drug-Coated Balloon Angioplasty for the Endovascular Treatment of Heavily-Calcified Femoropopliteal and Popliteal Lesions. Journal of Endovascular Therapy. 2023((Kronlage, Bertele, Linden, Frey, Erbel) Department of Cardiology, Angiology and Pneumology, Heidelberg University Hospital, Heidelberg, Germany).

398. Krishnan P, Faries P, Niazi K, Sachar R, Jain A, Brodmann M, et al. Stellarex Drug-Coated Balloon for the Treatment of Peripheral Artery Disease: Five-Year Results from the ILLUMENATE Pivotal Randomized Controlled Trial. American Journal of Cardiology. 2024;227((Krishnan, Tarricone) Department of Cardiology, Cardiovascular Institute, Mount Sinai Hospital and Icahn School of Medicine at Mount Sinai, NewYork, New York, United States(Faries) Division of Vascular Surgery, Mount Sinai Hospital, NewYork, New York, Uni):83-90.

399. Rezvani F, Heider D, Konig HH, Herbarth L, Steinisch P, Schuhmann F, et al. Telephone Health Coaching and Remote Exercise Monitoring (TeGeCoach) in Peripheral Arterial Occlusive Disease A Randomized Controlled Trial. Deutsches Arzteblatt International. 2024;121(10):323-30.

400. Cawich I, Armstrong EJ, George JC, Golzar J, Shishehbor MH, Razavi M, et al. Temsirolimus Adventitial Delivery to Improve ANGiographic Outcomes Below the Knee. Journal of endovascular therapy : an official journal of the International Society of Endovascular Specialists. 2024;31(4):562-75.

401. Clavijo LC, Caro J, Choi J, Caro JA, Tun H, Rowe V, et al. The addition of evolocumab to maximal tolerated statin therapy improves walking performance in patients with peripheral arterial disease and intermittent claudication (Evol-PAD study). Cardiovascular revascularization medicine : including molecular interventions. 2023;55(101238551):1-5.

402. Heider D, Rezvani F, Matschinger H, Dirmaier J, Harter M, Herbarth L, et al. The effect of telephone health coaching and remote exercise monitoring for peripheral artery disease (TeGeCoach) on health care cost and utilization: results of a randomized controlled trial. The European journal of health economics : HEPAC : health economics in prevention and care. 2024;25(4):615-29.

403. Sandberg A, Nordanstig J, Cider A, Jivegard L, Hagstromer M, Back M. The Impact of Nordic Pole Walk Advice Alone or in Combination With Exercise Strategies on Daily Physical Activity in Patients With Intermittent Claudication: A Randomized Clinical Trial. Physical therapy. 2023;103(11).

404. Hagen P, Malatesta D, Calanca L, Mazzolai L, Lanzi S. The PAD-adapted 30-20-10 during Nordic walking: A new exercise training session in patients with symptomatic peripheral artery disease. Medicine. 2024;103(25):e38601.

405. Torsello GF, Stavroulakis K, Bisdas T, Cardona Y, Wichmann K, Torsello GB. Treatment of Femoropopliteal Artery Disease with Polymer-Coated Drug-Eluting Stent: 5-Year Results of a Prospective, Non-Randomized Study Including the Halo Phenomenon. Cardiovascular and interventional radiology. 2024;47(2):177-85.

406. Ozpak HB, Aydin C, Demirkiran A. Twelve-Month Outcomes of a Novel Iopromide-Based Paclitaxel-Coated Balloon for the Treatment of Chronic Total Occlusion of Femoropopliteal Arteries. Turk Kardiyoloji Dernegi arsivi : Turk Kardiyoloji Derneginin yayin organidir. 2023;51(2):112-8.

407. Enzmann FK, Nierlich P, Holzenbein T, Aspalter M, Kluckner M, Hitzl W, et al. Vein Bypass Versus Nitinol Stent in Long Femoropopliteal Lesions: 4-Year Results of a Randomized Controlled Trial. Annals of surgery. 2023;277(6):e1208-e14.

408. Bosiers MJ, De Donato G, Torsello G, Silveira PG, Scheinert D, Veroux P, et al. ZILVERPASS Study: ZILVER PTX Stent versus Prosthetic Above-the-Knee Bypass Surgery in Femoropopliteal Lesions, 5-year Results. Cardiovascular and interventional radiology. 2023;46(10):1348-58.
